# Effects on groundwater storage of restoring, constructing or draining wetlands in temperate and boreal climates: a systematic review

**DOI:** 10.1186/s13750-022-00289-5

**Published:** 2022-12-08

**Authors:** Arvid Bring, Josefin Thorslund, Lars Rosén, Karin Tonderski, Charlotte Åberg, Ida Envall, Hjalmar Laudon

**Affiliations:** 1 The Swedish Research Council for Environment, Agricultural Sciences and Spatial Planning (Formas), P.O. Box 1206, 111 82 Stockholm, Sweden; 2 Department of Physical Geography, Stockholm University, 106 91 Stockholm, Sweden; 3 Department of Architecture and Civil Engineering, Chalmers University of Technology, 412 96 Gothenburg, Sweden; 4 Department of Management and Engineering, Linköping University, 581 83 Linköping, Sweden; 5 Department of Forest Ecology and Management, Swedish University of Agricultural Sciences, 901 83 Umeå, Sweden

**Keywords:** Hydrology, Hydrogeology, Bog, Fen, Mire, Peat, Environmental management, Sweden, Ditching, Ditch blocking

## Abstract

**Background:**

Drainage activities have caused widespread wetland loss, groundwater drawdown and impairment of ecosystem services. There are now several national programs for wetland restoration, primarily focused on reintroducing ecosystem services such as habitats and nutrient retention. In Sweden, recent dry summers have also reinforced interest in hydrological functions such as the potential for enhanced groundwater storage, both in and around the wetland. However, there are several knowledge gaps regarding groundwater storage effects of restoration, including if they extend beyond the wetland and how they vary with local conditions. Therefore, we have systematically reviewed groundwater storage effects from the interventions of restoring, constructing or draining boreo-temperate wetlands. Drainage was included primarily to evaluate to what degree restoration can reverse drainage effects.

**Methods:**

We searched 8 databases for scientific journal publications in English, Swedish, Norwegian, Danish, French, German and Polish. Gray literature was searched in English and Swedish. Articles were included based on their relevance for Swedish conditions, i.e., in previously glaciated areas with boreal or temperate climate. Extracted outcome data were groundwater level changes, along with other variables including type of wetland and intervention and, when reported, distance between sampling point and intervention. Meta-analyses were conducted separately for studies that reported groundwater levels at different distances and studies that reported overall effects. Included studies were subject to critical appraisal to evaluate their susceptibility to bias, primarily selection bias, performance bias, and detection bias. Critical appraisal results were used in sensitivity analysis.

**Review findings:**

Out of 11,288 screened records, 224 articles fulfilled the criteria, and from these, 146 studies were included in meta-analysis. Most studies (89%) investigated peatlands, primarily from Finland, the UK and Canada. Restoration and drainage studies were equally common. Only nine studies reported measurements beyond the wetland area. Our synthesis is therefore primarily focused on effects within wetlands. In peatland restoration, the observed groundwater level rise decreased exponentially with distance from the restored ditch and was reduced to 50% after 9 [95% confidence interval: 5, 26] m. Drainage reached somewhat farther, with 50% of the groundwater drawdown remaining at 21 [11, 64] m. On average, restoration increased groundwater levels by 22 [16, 28] cm near the intervention, whereas drainage caused a drawdown of 19 [10, 27] cm. Assuming that sampling was unbiased, effects were similar for bogs, fens and mires. Restricting the meta-analysis to the 58% of studies that were of high validity did not alter conclusions.

**Conclusions:**

Effects of peatland restoration and drainage were of similar magnitudes but opposite directions. This indicates that, on average, rewetting of drained peatlands can be expected to restore groundwater levels near the ditch. However, restoration may not reach all the area affected by drainage, and there was a strong dependence on local context. For managers of wetland projects, it is thus important to follow up and monitor restoration effects and reinforce the intervention if necessary. Our results also point to a need for better impact evaluation if increased storage beyond the restored wetland area is desired.

**Supplementary Information:**

The online version contains supplementary material available at 10.1186/s13750-022-00289-5.

## Background

Wetlands provide many ecosystem functions in the landscape, including habitats for various species, water quality improvements, climate regulation, flood control and carbon sequestration [1, 2]. They occur in diverse physiographic settings, with hydrological processes governing both where they occur and how they function and provide ecosystem services [3]. For example, wetland water storage capacity and dynamics impact nutrient and pollutant removal, e.g., by increasing water residence times and sedimentation rates [4]. The predominantly oxygen-free conditions in wetlands also slow down biogeochemical processes, resulting in carbon sequestration in most wetlands as CO_2_ taken up by plants is transferred to long-term soil organic C storage [5].

Despite these benefits, drainage of wetlands to increase land availability for agriculture, forestry and infrastructure has been common for centuries. Human alterations of the hydrological landscape, in combination with hydroclimatic stressors, have reduced the areal wetland cover globally [6]. Estimates suggest that about 87% of all wetlands have been degraded, and that half of this change has taken place during the last 100 years [7]. In high latitudes, most wetlands are peatlands with important carbon sequestration functions. Across this region, and especially in Fennoscandia, wetland drainage started for agricultural purposes in the 17th to nineteenth century and peaked with forest productivity enhancement in the twentieth century [8]. After Finland and Russia, Sweden has the most drained peatlands in the world, covering several million hectares of land [9]. Many drainage ditches have resulted in new areas of productive forests, while others have only led to large-scale peatland degradation.

With ongoing climate change and biodiversity losses, there is however an accelerating attention to restore degraded wetlands. For instance, with increasing water stress in a warmer climate, wetland restoration interventions to keep water in the landscape is likely to become more common. In Sweden, recent dry summers have reinforced interest in the hydrological functions of wetlands, and if restoration can lead to increased groundwater storage, both in and around drained wetland areas. This can be of particular importance in sandy soils in the relatively dry areas in southeastern Sweden. In response to recent extreme droughts, the Swedish government allocated over 300 million SEK for wetland restoration [10]. Yet, the science underpinning desired outcomes of such restoration is somewhat limited, at least when it comes to effects on groundwater. For instance, although it is well established that wetland drainage can negatively impact local wetland surface water and groundwater storage [11], most knowledge has been derived from studies performed at individual wetland scales [12]. There are still knowledge gaps on how far such effects reach beyond the wetland, and how effects vary with local-regional hydro-climatic and environmental conditions [13].

**Fig. 1:**
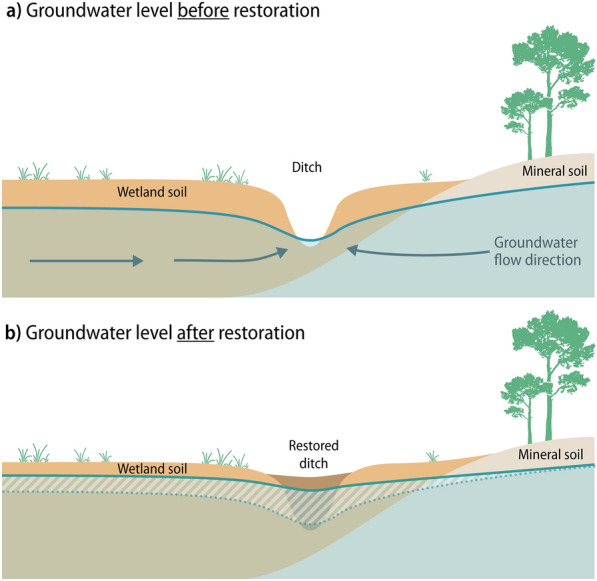
Illustration of groundwater storage change after wetland restoration. In a drained wetland (**a**), a ditch causes drawdown of the groundwater in the vicinity of the ditch. After restoration of the ditch (**b**), the groundwater level rises, possibly also in the adjacent soil if it is permeable and in hydraulic contact with the ditch

In Sweden, 14% of the productive forest area consists of drained peatlands (National Forest Inventory, unpublished). Both peatlands and other types of wetlands are typically located in topographic depressions, where the groundwater level is close to the surface and discharged from the subsurface. In terms of groundwater effects, ditching a peatland drains the soil near the ditch, but the lateral extent of the effect is uncertain (Fig. 1). Typically, peatlands have a surface layer of poorly degraded plant residues where water flows rapidly. In this layer, the drainage effect can be noticed relatively far from a ditch, even if the absolute changes are small [14]. Effects at distances of hundreds of meters, or even farther, are mentioned in the literature [14–16]. In deeper layers with more degraded organic material, the hydraulic conductivity is lower and groundwater level effects are mostly restricted to the vicinity of the ditch. However, if more conductive layers are present effects can extend farther away also at depth [17]. Conversely, effects may be restricted even at the surface if the entire soil profile has low hydraulic conductivity [17]. Thus, even though the principal mechanism of groundwater effects from wetland drainage and restoration is relatively straightforward, the size of the effect varies in complex ways, and is less well known for areas outside the actual wetland.

The Swedish Geological Survey (SGU) has investigated the conceptual foundations for increased groundwater storage from wetland restoration in Sweden [17]. For Swedish peatlands, drainage effects on groundwater is treated in [18], and environmental consequences of drainage on both forested and farmed lands in Sweden are reviewed in [19]. Model studies investigating surface water effects of wetland restoration or drainage in Sweden also exist (e.g., [20]). A recent report investigated the same question we ask here, although in a case study with limited data [21]. Furthermore, there are restoration guidelines that describe the hydrological processes in some detail, e.g., a report summarizing 25 years of experiences from peatland restoration in Finland (chapter 3 in [22]). Despite these reports, an overview is missing of the research literature on groundwater storage effects from restoring or draining wetlands, particularly for evidence of effects outside the wetland soil.

This study aims to address the above-mentioned knowledge gaps on how wetland groundwater storage is affected by restoration and by the historical legacy of wetland drainage, in the boreo-temperate landscape, we have systematically reviewed both peer-reviewed and gray literature (e.g., reports from government agencies). Using the evidence identified we provide a meta-analysis of effects on groundwater storage. The analysis also provides an indication of uncertainty ranges and weaknesses in the available literature.

### Stakeholder engagement

The principal stakeholder is the Swedish Geological Survey (SGU), who suggested the topic be investigated. During the writing of the protocol [23], consultations were held with SGU and several additional stakeholders, primarily government agencies with a mandate that involves wetland restoration, construction, and drainage. These include the Swedish Environmental Protection Agency, the Swedish Meteorological and Hydrological Institute, the Swedish Food Agency, and regional County Administrative Boards who oversee wetland restoration projects. Additional consultations were held with municipalities and non-government associations, including forest and farm owners, as well as nature conservation societies. During the review process, an additional number of consultations were held with the core stakeholders SGU and the Swedish Environmental Protection Agency. Several researchers were also contacted with specific questions. The review team, consisting of the authors of the review, was however independent and made all decisions on the scope and conduct of the review. A broader group of stakeholders, including all the ones listed above, were invited to comment on both the protocol and on this systematic review. Minor comments were submitted but did not alter the review.

### Objective of the review

The purpose of this review was to provide information that can support groundwater-related decisions about wetland restoration and drainage in previously glaciated terrain in temperate and boreal climates. Such decisions can be related to the efficacy of wetland intervention for groundwater storage change in the wetland itself, but also in the surrounding landscape. The results of the review are needed for assessing the possible effects of wetland restoration, creation and drainage in various hydrogeological settings relevant in a Swedish context.

The primary question is: What is the effect of wetland restoration, construction, and drainage on groundwater storage in temperate and boreal climates? Of particular interest are effects in areas that are adjacent to the wetland, in addition to within the treated wetland. We expected, however, that studies on such effects were few, and therefore also included studies that have reported effects within the affected wetland only. Effects in soils adjacent to the wetland was not a separate question, but we report such effects separately as they were of primary interest to the stakeholders. Lacking direct information on effects outside the wetland, stakeholders communicated that it can be useful to know how far effects extend from the intervention within the wetland. Therefore, we formulated a secondary question, not restricted to effects outside the wetland: What is the size of the overall effect on groundwater storage, and how far does it extend from the intervention? Further secondary questions are related to how various factors modify the effect, such as wetland type, soil type, and geographical settings. The questions can be briefly defined by the following PICO elements:

Population: Groundwater in temperate and boreal climates in previously glaciated areas.

Intervention: Restoration, construction or drainage of wetlands.

Comparator: No intervention.

Outcome: Groundwater level, storage or amount.

These elements are further defined in the section on article screening and study inclusion criteria below.

## Methods

The protocol for this review was published by Bring et al. [23]. In the protocol, methods and criteria for relevance and validity were described prior to the study, in accordance with guidelines from the Collaboration for Environmental Evidence [24]. The reporting of this systematic review follows the ROSES reporting standards [25] (see Additional file 1).

### Deviations from the protocol

As described above, a secondary question on how far effects extend from the intervention was added to address the specific interest of stakeholders outlined in the protocol. Full text screening was double blinded, in accordance with the protocol, but for some articles, blinding may have been unintentionally compromised by reviewer notes that were accessible to other screeners in the system. For blinding to be compromised in practice, however, it required both that there was a note revealing the decision, implicitly or directly, and that the note was read by the other reviewer. At most, this could have affected up to 41% of articles (92 out of 224 included articles have comments), but the real number is probably lower since not all comments discussed eligibility nor were read by the other reviewer.

Articles with conflicting decisions were first reviewed for misunderstandings by the two reviewers, thereafter among all co-authors in case of remaining disagreement. This was a deviation from the protocol, which states that any disagreements were to be reconciled through discussion with all co-authors.

#### Search for articles

An extensive search for peer-reviewed scientific articles and gray literature was conducted in bibliographic databases, search engines, websites of relevant organizations and through stakeholder contacts.

#### Bibliographic databases

An initial search was performed in September 2020 in the eight bibliographic databases listed in Table 1. An updated search was performed in December 2021 in five of the bibliographic databases (Scopus, Web of Science Core Collection, Academic Search Premier, CAB Abstracts and ProQuest Natural Science Collection).

**Table 1:** Bibliographic databases used to search for articles

Database/platform	Search field	Language of search terms	Subscription information
Scopus	Title, Abstract, Keywords	English	Swedish research council formas subscription
Web of science core collection	Topic (search the fields: title, abstract and keywords)	English	Swedish Research Council Formas subscription includes: science citation index expanded; Social sciences citation index; Arts & humanities citation index; Conference proceedings citation index-science; Conference proceedings citation index-social science & humanities; Emerging sources citation index
Academic search premier	Title, Abstract, Subject Terms, Author-Supplied Keywords	English	Swedish research council formas subscription on Ebsco platform
CAB abstracts	Title, Abstract, Heading Words	English	Swedish research council formas subscription on Ovid platform
Directory of open access journals	All fields	English	Free, does not require a subscription
DiVA	All fields	English and Swedish	Free, does not require a subscription
ProQuest natural science collection	Title, Abstract, All subjects & indexing	English	Swedish Research Council Formas subscription includes: AGRICOLA; Agricultural Science database; Aquatic sciences and fisheries abstracts; Biological science database; Biological science index; Earth, atmosphere & aquatic science database; Environmental science database; Environmental science index; Meteorological & geoastrophysical abstracts
SwePub	All fields	English and Swedish	Free, does not require a subscription

Following the protocol [23], we developed a search string that consisted of two search blocks, the first with intervention terms (restoration, construction or drainage of wetlands) and the second with outcome terms (change in groundwater level, storage or amount). All information about the searches is provided in Additional file 2. This file includes dates of searches, database and platform information, how the search strings were adapted to the search capabilities of each database, limits to the search, and the number of hits found in each search. Searches were performed with English search terms, except for DiVA and SwePub where Swedish search terms were also used. Since non-English articles often have a title and abstract in English, the use of English search terms retrieved articles written in several different languages. The searches were set to include articles written in English, Danish, French, German, Norwegian, Polish, and Swedish. The searches were not limited by publication date or document type.

#### Search engine

A search was performed using the search engine Google Scholar in September 2020. Because Google scholar does not allow long search strings, we used seven simple search strings, four in English and three in Swedish (see Additional file 2). Results were ranked by relevance and the first 200 records for every search were exported from Google Scholar using Publish or Perish version 6 software [26].

#### Websites of relevant organizations

To find gray literature, we searched the websites of 52 organizations. The search capabilities differed between the websites; sometimes Boolean operators could be used and sometimes the website required browsing through its pages. We used search terms in Swedish or English and sometimes both, depending on the website. All search terms used and the number of matching results are provided in Additional file 2.

#### Supplementary searches

We received studies and reports from four Swedish stakeholders and experts in the field that were contacted during the project. Furthermore, during the article screening, we identified five relevant reviews [27–31], the bibliographies of which we screened to find additional studies. We also screened the bibliographies of the articles included in this systematic review.

#### Assembling and managing search results

The results of all searches were collated using the reference management software EndNote. Duplicates were removed using the de-duplication method described in [32] and [33], the latter for the updated searches.

### Article screening and study eligibility criteria

Articles were screened in two stages. First, all articles were screened on title and abstract using the online platform Rayyan (https://www.rayyan.ai/). At this stage, articles were classified into three categories: (1) include, (2) exclude, and (3) probably exclude. Before commencing full screening, consistency of screening decisions at the title and abstract level was checked for a subset (n = 300) of abstracts, by independent reviewers in pairs. Conflicting decisions were made for 39 abstracts, or 13%. Four of the cases were resolved as resulting from classifying review studies differently. The level of agreement was deemed acceptable and in accordance with the protocol (above 80%), and remaining abstracts were therefore single-screened by AB. The articles classified as “probably exclude” (n = 29) were double-screened by IE. Due to the large volume of abstracts, articles excluded at the title and abstract stage were not coded with a reason for exclusion.

Articles that passed title and abstract review were uploaded to the systematic review platform Covidence (https://www.covidence.org) for full text review. At the full text stage, all articles were screened by AB and one additional author. Decisions on all articles were made independently. As noted earlier, however, independence was sometimes compromised by visible author notes. Reviewer agreement at the full text screening was 79%. Disagreements were initially resolved by the reviewer pair, and in case of remaining disagreement by the review team in consensus.

When several studies reported data from the same investigation, we prioritized peer-reviewed studies over PhD and MSc theses, and excluded the latter as redundant. Similarly, we also prioritized studies with longer time series or more extensive or accessible reporting, and excluded the redundant studies with less data. No review authors were allowed to make decisions on articles on which they themselves were a co-author. A list of articles excluded at full text, together with reasons for exclusion, is provided in Additional file 3. The list of included articles is provided in Additional file 4.

### Eligibility criteria

#### Eligible populations

The review focuses on groundwater and geological conditions relevant for Sweden. Studies therefore had to be performed in Köppen-Geiger climate classification zones *BSk*, *C*, or *D* (cold semi-arid climates, temperate climates, or continental climates). Studies should also have been performed in areas that had been subject to glaciation during the Quaternary geological period (last 2.5 million years). Eligibility according to the glaciation history criterion was determined on a case-by-case basis, aided by maps of Quaternary glaciation extent [34]. In cases of doubt, we evaluated the study site description to determine eligibility.

#### Eligible interventions

Since restoration and construction effects are distinct from drainage effects, we defined two sets of eligible interventions. We included drainage interventions and not only restoration and construction interventions for three reasons. First, the original drainage effect is relevant for evaluating how far restoration could go towards undrained wetland conditions. Second, several stakeholders have an interest in drainage effects for permit applications, for a limited number of extended drainage projects or maintenance of present drainage. Third, in cases where it is not possible or desirable to fully restore a wetland, limiting or controlling drainage may still achieve some benefits of elevated groundwater levels.

#### Restoration or construction of wetlands

Included: Restoration or construction actions that aimed to partially or fully restore or create wetland conditions, for example: ditch blocking, check dams, dam restoration, damming, restored shoreline wetlands (wetlands created as result of lake level change or restoration), vegetation removal, remeandering of streams, riparian overflow zones, ditch overflow zones, farm ponds, nutrient retention ponds, wetland construction on soil that was not a wetland prior to the intervention.

Not included: Water reservoirs that were not wetlands (e.g., deep reservoirs for hydropower or irrigation), subsurface flow treatment wetlands, constructed treatment wetlands that were not connected to the surrounding soils/groundwater, artificial raising of the water table with specific amounts as a control variable (unless independent groundwater outcomes were reported for other parts of the wetland, or in adjacent soils).

#### Drainage of wetlands

Included: Drainage actions that aimed to drain wetland areas partially or fully, for example ditching, ditch maintenance or re-excavation to original depth, ditch deepening, dam removal, other water table lowering measures.

Not included: Drained soils that were not or had not been wetlands, subsurface drainage, drainage through pipes or pumping, drainage of constructed wetlands that were not connected to the surrounding soils/groundwater, artificial lowering of the water table with specific amounts as a control variable (unless independent groundwater outcomes were reported for other parts of the wetland, or in adjacent soils).

#### General

We excluded the following wetland types: Coastal wetlands strongly influenced by tides, salt water or brackish waters.

#### Eligible comparators

The study had to include a control. We excluded studies that only reported outcomes for variations of the intervention, e.g., studies of drainage with different ditch spacings but with no pristine wetland comparator. We accepted both before-after (BA) and control-impact (CI) studies if they were designed so that the effect of the intervention could be evaluated.

#### Eligible outcomes

Studies reporting measures that allowed direct assessment of groundwater level, storage or amount were included. The outcome had to be reported within the wetland, or in its near vicinity, both for the intervention site or time and a corresponding control site or time (or both).

Groundwater measurements outside the wetland (apart from control sites) were of particular interest and hence noted in the metadatabase table where all descriptive data about the studies was collected. We also accepted hydraulic head, pump tests, and other measurements that could be directly interpreted as groundwater levels or amounts. Studies with only surface water level measurements of different kinds (in situ and aerial/satellite), such as the depth of water in the wetland, were not included. For the secondary question, groundwater measurements had to be reported at different distances from the intervention.

#### Eligible study types

All types of experimental and observational studies were included. The following study types were excluded: Laboratory studies, greenhouse studies, and model studies that did not report empirical validation data fulfilling our eligibility criteria.

#### Study validity assessment

We did not appraise external validity, but the criteria for study inclusion were formulated to ensure external validity for Swedish conditions. The criteria for internal validity (Table 2) were formulated as in the protocol. In the internal validity assessment, we attempted to abide by the recommendations of [35], similar to the risk of bias tool in Cochrane reviews. The criteria were intended to aid judgement of risk of bias with a focus on selection bias and performance bias. The purpose of the critical assessment was to reduce the risk that the conclusions of the review are misleading.

**Table 2:** Criteria for assessing study validity

	Low susceptibility to bias	High susceptibility to bias	Risk of bias type
Study design			
Comparison domain	Comparison across both space and time (e.g., BACI)	Comparison across space or time only (e.g., CI, BA)	Selection
Control matching at study initiation (not applicable to BA studies, where control and intervention take place at the same site)	Well-matched control and intervention sites, close in proximity or clearly similar, but without spillover effects	Well-matching not evident, other factors likely to influence difference between treatment and control sites	Selection
Effect modifiers and confounding factors during study period	No or minimal presence of co-interventions, co-exposures or trends present that are likely to differ between groups, baseline environmental conditions similar to intervention period; or if present, confounding factors are accounted for	Co-interventions, co-exposures or trends present that are likely to differ between groups, e.g., differing climate conditions, weather events, or other environmental or anthropogenic changes; and factors are not accounted for	Performance
Groundwater measurements			
Measurement similarity and representativeness	Similar and representative measurements across groups, unlikely to differ systematically	Systematic differences in measurements, outcomes not assessed similarly, or measurements unlikely to be representative	Detection
Other			
Confounding variables not included in other risk of bias types (e.g., funding source)	No obvious confounding factors, or confounding factors present but accounted for	Confounding factors stated or obviously present but not accounted for	Other

Before full implementation, we tested the criteria by carrying out blinded assessment by two reviewers (AB and JT) on a subset of 20 articles that contained 29 separate studies. The agreement was deemed satisfactory as less than 12% of assessment decisions differed. Three additional articles were assessed by the review team in blinded pairs, with judgements differing in two out of the total of fifteen criteria decisions. The remainder of the articles were critically appraised by one author (AB), with decisions subsequently reviewed by another author to ensure consistency. Any disagreements that were not resolved between the two reviewers were discussed with the entire review team. Authors of the review were not allowed to perform critical appraisal of their own work. No studies were excluded due to low validity, but study validity was used in sensitivity tests. In these tests, we considered studies with at least four out of five criteria rated as “Low risk of bias” to be of high validity.

### Data coding and extraction strategy

#### Metadata extraction and coding

We prepared an Excel spreadsheet for metadata extraction and presentation. The spreadsheet was first tested on a subset of 20 articles by two reviewers independently (AB and JT). The initial testing resulted in some modifications to the table and the predetermined coding options. Data were subsequently single-extracted for each study. This work was done by two reviewers (AB and JT) who extracted different parts of the literature, with discussions held in cases of doubt. Data were extracted into a shared spreadsheet, with any comments or revisions immediately visible to the other reviewer. In applicable columns, the coding was locked to fixed alternatives, listed on the “Drop-down lists” sheet in the file with all extracted data (Additional file 5). For other columns, coding instructions on the same sheet guided how to extract and enter values. The fixed alternatives and guidance for coding served to ensure reproducible and transparent data extraction. If there were several independent comparisons or wetlands reported in the same article, these were entered as separate lines in the database table.

Metadata extraction focused on wetland type, geographical context, study design, and intervention and sampling details. Study coordinates were transferred directly as reported. When no coordinates were presented, included maps were used to identify locations. If both coordinates and maps were missing, place names and descriptions were used. In both the latter cases, Google maps (http://maps.google.com) was used to determine approximate coordinates. Coordinates in the final table should therefore not be interpreted as exact locations of study sites. The full table with all extracted data is available in Additional File 5.

#### Data extraction for meta-analysis

Before commencement of extraction of outcome data, a pilot extraction was performed on five typical studies. Methods and results from the pilot extraction were discussed with the entire review team. AB then performed all data extraction of outcome data for meta-analysis. A randomly selected subset of 10% of the studies was cross-checked by JT to ensure consistent extraction. The cross-check revealed minor uncertainties in extraction (different labeling conventions, superfluous data remaining), but calculations and effect size data used for meta-analysis were all verified. For studies published 2010 or later, we asked authors for missing information. The study was included if we received sufficient information; otherwise, it was left out of the meta-analysis. One article could contain more than one study if there were multiple independent wetland comparisons (see Additional File 6 for a more detailed discussion of study independence).

We have not included other outcomes than groundwater levels in the meta-analysis. Other outcomes that could have been informative, such as estimates of changes in groundwater storage, turned out to often be based on various assumptions or models that were not standardized between studies. Evaluation of such outcomes therefore risked including variations in assumptions as well as true differences in storage. We expect no substantial effects from omitting these outcomes as most studies with other outcomes reported groundwater levels as well (n = 3 restoration and n = 2 drainage studies did not and were therefore not included in meta-analysis).

Meta-analyses were carried out for two types of investigations: the relationship between change in groundwater level and distance from intervention, and the overall change in groundwater level reported for the wetland. For the first type, the meta-analyses of groundwater level change related to distance involved two types of effect sizes based on function parameters that were comparable across studies (see explanation below). For the second type, the raw mean difference of the overall groundwater level was used as effect size. All meta-analyses were performed separately for restoration and drainage. In all, six meta-analyses were carried out.

For the first type of investigation, we only estimated the relationship between change in groundwater level and distance for studies that reported separate groundwater level values at distinct distances from the intervention. In most cases, these studies investigated ditches that were excavated or filled in, with multiple groundwater wells placed along transects perpendicular to the ditch or ditches. For those studies, each outcome value (i.e., the change in groundwater level recorded at a particular well) was noted, and the logarithm of its distance from the intervention was calculated. These separate values at different distances were then used to fit a logarithmic regression between the distance and the change in groundwater level. The regression function was expressed as

$$y = m\ln x + b$$

where *y* is the change in groundwater level, ln *x* is the natural logarithm of the distance from the intervention, and *m* and *b* are coefficients. The regressions were fit in Excel [36] using the LINEST function. For each study, the coefficients *m* (slope) and *b* (intercept) were calculated as two separate effect sizes of the study, together with their corresponding standard errors. By combining groundwater change at different distances into a function (the parameters of which were used as effect sizes), we were able to use meta-analysis of all studies to estimate a common slope and intercept of the function. Since the underlying data were on the same nominal scale (groundwater level change in cm, distance from the intervention in m), the slopes and intercepts were directly comparable across studies. The purpose of this simple model was to condense information from many measurements in a straightforward way.

For the second type of investigation, we analyzed overall groundwater effects for the wetlands, calculated as the raw mean difference in groundwater levels, without consideration of distance. This analysis depended on the sampling locations chosen by the investigators of each study and was therefore representative of overall wetland groundwater effects to the extent that sampling in the individual studies was representative. We did not evaluate the sampling representativeness for each wetland, as this was often difficult due to limited reporting, but for each study we evaluated the risk of bias due to sampling differences between control and intervention groups, as part of the critical appraisal.

Further details on the data extraction strategy and study independence are reported in Additional file 6. The summary extracted effect sizes and variances are provided in Additional file 5.

### Potential effect modifiers and reasons for heterogeneity

A secondary question of this review was how various factors influence the effect size. At the early stage of the review, we performed a pilot data extraction, identified potential modifiers that were possible to evaluate, and discussed with stakeholders how these could be expected to modify the effect. As stated in the protocol, this resulted in addition of some moderators not explicitly listed there, as it was not known at the protocol writing stage that they would be possible to evaluate. The final decisions on which moderators to include were made solely by the review team. Many studies did not report data in sufficient detail for all factors to be evaluated. The following list shows moderators that were included in at least one meta-analysis, and how we expected them to possibly modify the effect:

- Wetland type. It is reasonable that effects may differ between fens, which are groundwater-fed, and bogs, which are precipitation-fed. Limnic shore wetlands, which lie along rivers or lakes, can also be expected to behave differently than other wetlands.
- Wetland slope. The slope of the wetland surface may influence the slope of the groundwater surface, which can in turn lead to larger or smaller detected effects, depending on how sampling is carried out.
- Underlying soil type. Expected direction: larger effects where the soil is impervious and water transport is restricted to the wetland.
- Peat depth. Peat depth is associated with the degree of peat decomposition, which in turn affects the hydraulic conductivity of groundwater.
- Climate zone. The included climates are rather similar, but we expected that effects could be larger in relative terms in dry climates (*BSk*, compared to others).
- Intervention type. Expected direction: larger effects for more permanent intervention types. For example, we expected that damming drains would have larger effects than spontaneous restoration (unmaintained drainage), and that ditching would have larger effects than ditch network maintenance.
- Intervention magnitude. Expected direction: larger effects with increasing magnitude, i.e., greater height of dams or deeper ditches.
- Time since intervention. Expected direction: larger effects with increasing time, although there are cases where interventions deteriorate over time too.
- Ditch spacing. Closer ditch spacing should increase the effect.
- Study design. There is a risk that sampled differences are larger in less robust designs (CI and BA).
- Type of replication. Because the sampled variation can be smaller if interventions are not appropriately replicated, pseudoreplicated studies risk having narrower confidence intervals due to non-independence between intervention sites.
- Reference point for groundwater measurements. For drainage studies, the effect should be larger if measurements are taken against a fixed datum, as opposed to the ground surface, because of soil compaction that follows drainage.
- Maximum distance from ditch of groundwater sampling. The effect should decrease if sampling points farther from the ditch are included.
- Type of transect (open-ended or closed, i.e., bounded by next ditch). Effects should be larger between ditches, as opposed to along transects with no next ditch.

We expected the statistical heterogeneity, measured with the *I*^*2*^ statistic, to be large (above 50). This is because wetlands can vary substantially, and many factors can influence local effects in particular contexts. The source code for all analyses is available in Additional File 10. The resulting statistics of all tests, including values of *Q*, *τ*^*2*^, and *I*^*2*^, are available in Additional File 7. Effects of moderators are also discussed in Additional File 8.

### Data synthesis and presentation

The review findings were synthesized in several ways, both qualitative and quantitative, described below.

#### Descriptive analysis and narrative synthesis

We first calculated statistics and performed a narrative synthesis for key parameters that describe the body of evidence. This gives an overview of the available research and illustrates what types of studies are predominant in the literature. We focused on metadata and descriptive characteristics, saving discussions of actual results for the quantitative analysis (see below). However, for studies for which no quantitative synthesis was possible, the narrative synthesis included both descriptive information and actual results and outcomes.

#### Quantitative synthesis

For quantitative synthesis of effect sizes, random-effect models were fitted in *R* [37] version × 64 4.1.2 using the *metafor* package v3.0.2 [38] and the function *rma* (https://wviechtb.github.io/metafor/reference/rma.uni.html). The heterogeneity as well as the effects of moderators were analyzed with addition of the *mods* keyword specifying the moderator (detailed coding available in Additional File 10).

Studies with few replications and pseudoreplicated studies provide biased estimates of variance, and since a meta-analysis normally weights studies by their inverse variance, this influences the estimates of meta-variance and meta-effect size [39]. It is possible to use weighting by sample size instead, which provides an unbiased estimate of the effect size [40]. As many studies in our analysis were based on either true replication with low *N* or pseudoreplication, we used the number of wetlands as study weights in the meta-analysis. This assigned higher weight to studies that sampled more wetlands, regardless of the study variance, and assigned the lowest weight to pseudoreplicated studies (based on a single wetland). We also used the Knapp-Harting adjustment [41–43] to partly compensate for the low number of replications [44].

A few studies presented data for different distances to the intervention for limnic shore wetlands, for example in the case of stream re-meandering or beaver dam construction. However, the limnic shore studies were too few and diverse in local wetland setting, research design and reporting to be incorporated into a meaningful meta-analysis of the effect of distance from the intervention. The meta-analyses of the effect of wetland interventions at different distances were therefore restricted to peatlands. Limnic shore wetlands were however included in the meta-analyses of overall wetland effects when not considering the distance from intervention.

For studies investigating effects at different distances from the intervention, we considered the slope of the relationship *m* the most important as it describes how quickly the effect changes with distance, whereas the intercept parameter *b* adjusts this relationship to different depths in the soil. Using the point estimates of *m* and *b*, we constructed an average relationship *y* = *m ln x* + *b* across all studies, with confidence intervals based on standard errors for the *m* and *b* estimates.

To help put results in context, we used results from meta-analyses of effects at different distances to calculate the total groundwater storage changes for various ditch spacings. In this analysis, we integrated the area under the curve *y* = *m ln x* + *b* from 1 m out to either the distance where the effect became zero or to the distance that was half-way to the next ditch, whichever was shorter. Since the area under the curve equals a volume per meter of ditch length, it was used to calculate total groundwater storage change per hectare. We assumed a porosity of 0.9 for the natural wetland soil subject to drainage, and 0.7 for previously drained soil that was restored, to account for compaction after drainage [45]. We chose ditch spacings of 40 m, 70 m and 100 m as illustrative values. The volume per hectare was then converted to mm groundwater storage.

#### Sensitivity analyses

Sensitivity analyses were performed both for aspects of our model specifications and for factors that may have influenced the findings in the primary studies, such as the extent of sampling. We tested how our results changed if studies were weighted by inverse variance, as is commonly done, instead of by the number of wetlands. We also tested how our results changed if we included only studies with true replication. Further, we investigated how the results changed when only high validity studies were included, and how different study designs (BA/CI/BACI) influenced the results.

## Review findings

### Review descriptive statistics

**Fig. 2:**
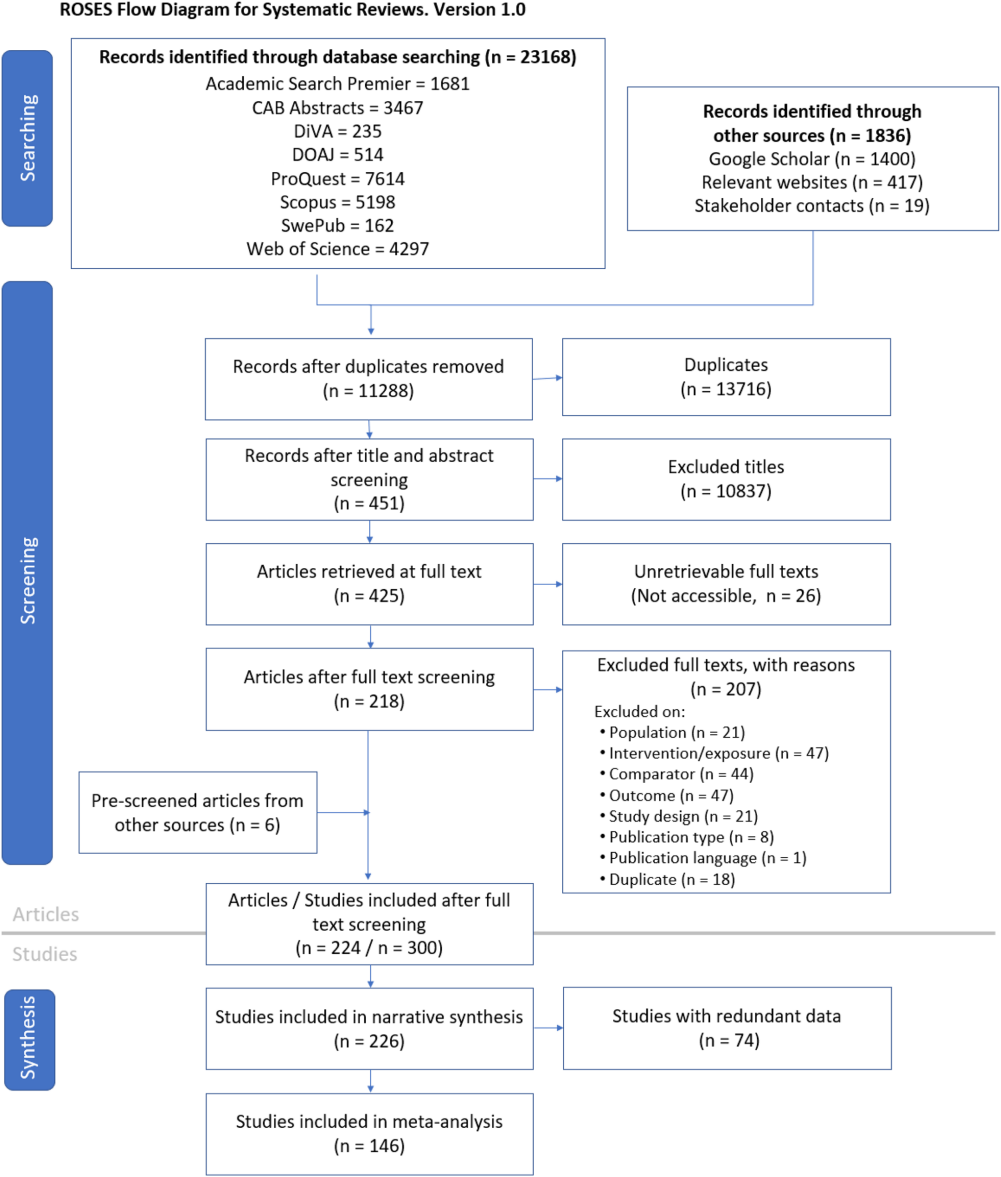
Flow diagram of literature screening stages

The initial searches in all bibliographic databases, Google Scholar and relevant websites resulted in 10,716 unique records (Fig. 2). An additional 12 unique records, mostly consisting of unpublished academic reports and conference papers, were provided by stakeholder contacts. The updated searches in December 2021 added 560 new items, bringing the total to 11,288. Finally, 6 additional records were identified from reference lists: 2 from relevant reviews found during screening and 4 from articles included in this systematic review.

The 224 included articles contained 300 studies of restoration, construction or drainage. Many articles allowed evaluation of both restoration and drainage, as well as multiple independent comparisons (for example, of physically different groups of wetlands with different times since intervention). The number 300 refers to studies of overall wetland effects. In some cases, one study of overall effects could be separated into several studies of effects at different distances from the intervention. All studies of effects at different distances also allowed a study of overall effects.

Out of the 300 studies, 69 were redundant for our purposes (i.e., groundwater data from the same location were published in another study) and thus excluded from descriptive statistics and meta-analysis. Another five studies were combined with other studies to extend the data coverage but not included separately. However, redundant studies were left in the meta-data table (sheets “Redundant” and “Combined”), as some include more detailed data that can be useful in other contexts. A list of articles excluded at full text, together with a reason for exclusion, is included in Additional File 3.

### Narrative synthesis including study validity assessment

#### Narrative synthesis of all studies

**Fig. 3:**
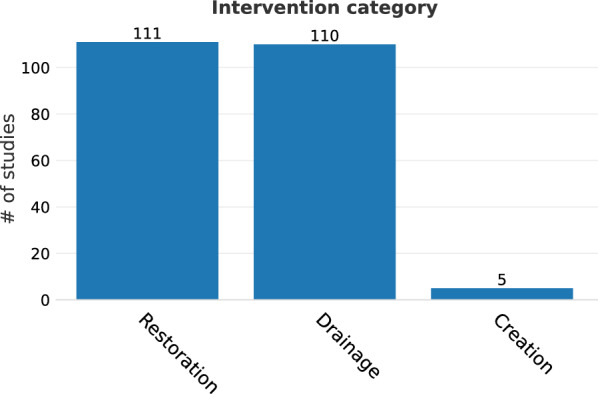
Number of included studies, separated by intervention category

Among the 226 remaining unique studies (from 167 articles), restoration and drainage occurred with similar frequency, while wetland construction on soil that had not previously been a wetland was uncommon. Restoration effects were reported in 111 studies (95 articles), creation in 5 studies (5 articles), and drainage in 110 studies (90 articles, Fig. 3).

**Fig. 4:**
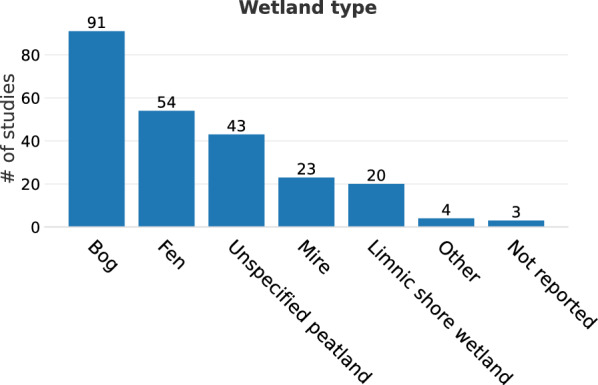
Number of studies by wetland type

**Fig. 5:**
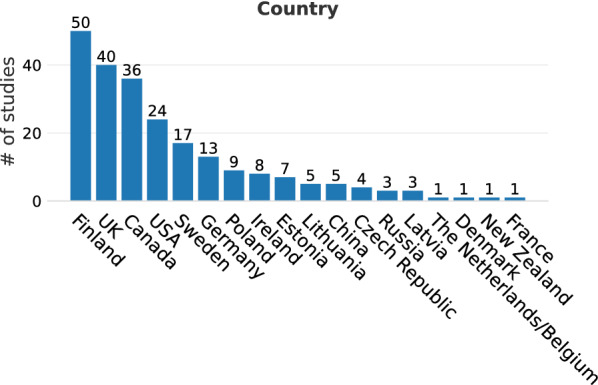
Number of studies by country

**Fig. 6:**
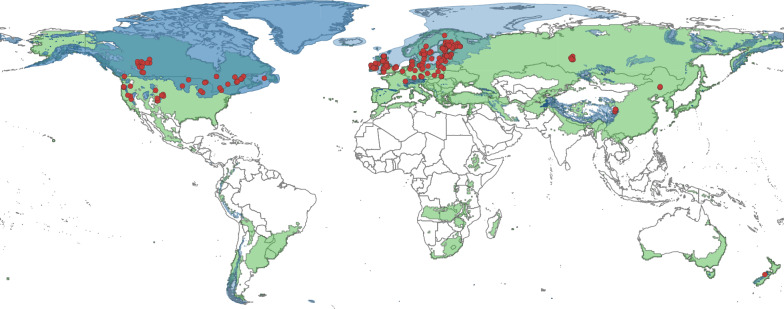
Locations of included studies. Red dots show study locations. Green shaded area shows eligible climate zones (Köppen Geiger climate zones BSk, C, D; based on [46]). Blue shaded area shows approximate extent of glaciation at the Last glacial maximum (LGM, based on data from [34]). Some areas outside the blue region were glaciated earlier during the Quaternary and therefore also included

**Fig. 7:**
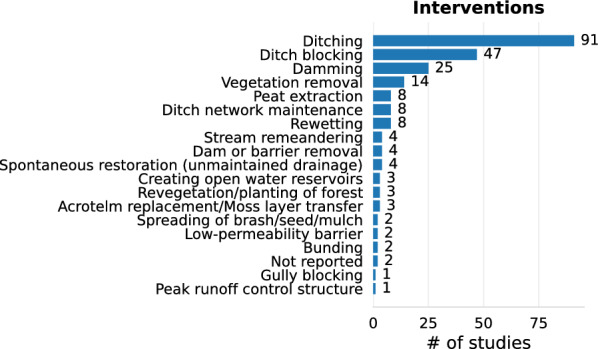
Frequency of wetland interventions in the included articles

The evidence base was dominated by studies on peatlands (bogs and fens correspond to 91 and 54 studies, respectively, with unspecified peatlands and mires making up another 43 and 23 studies, altogether comprising 89% of all studies; Fig. 4), most commonly from Finland (n = 50, Fig. 5) and the UK (n = 40). In total, 18 countries were represented, six of them with at least 10 studies (Finland, UK, Canada, USA, Sweden and Germany). The location of studies is shown in Figs. 6 and 7.

Restoration studies investigated a range of interventions, predominantly ditch blocking (n = 47). Other interventions included vegetation removal (n = 14), unspecified rewetting measures (n = 8), acrotelm replacement (n = 3; in some cases similar to seeding, n = 2) and stream remeandering (n = 4). Drainage studies overwhelmingly investigated the effects of ditching (n = 91), but 8 studies reported effects of ditch network maintenance, all of them from Finland. In two articles on drainage, a dam was either removed [47] or constructed [48] resulting in drained conditions in part of the wetland.

Even though we did not investigate greenhouse gases, such measurements were common, particularly in recent studies, and are indicated in the metadata table. The database is however not to be used as a systematic map of greenhouse gas studies, as this was not a criterion for our search and screening process. The full metadata table together with the data extraction coding is available in Additional file 5.

#### Study validity

The 226 studies were judged as having high or low risk of bias on each criterion, and tallies are presented in Table 3.

**Table 3:** Tallies of risk of bias judgement on the five criteria. Note that some rows sum to more than 226; this is because individual wetland sites in the same study sometimes were appraised differently

Criterion	Number of studies with			
	Low risk of bias	High risk of bias	Not possible to judge	Not applicable
Comparison domain	41	185	–	–
Control matching	135	33	15	47
Effect modifiers	169	46	12	
Measurement similarity	205	10	11	–
Other confounding factors	221	1	3	–

Out of the five study criteria, the three first (focusing on comparison domain, control matching and effect modifying factors during the study) resulted in a clear separation of studies, with 18%, 59% and 74% of studies judged as having low risk of bias for each criterion, respectively (Table 3). The two latter criteria (focusing on measurement similarity and any other possible confounding factors, respectively) did not discriminate much between studies, as most studies (91% and 98%, respectively) were judged to have a low risk of bias.

We considered studies that were judged to have low risk of bias on at least four out of the five criteria as high validity studies. In sensitivity tests, we compared this group of studies to the evidence base as a whole, to evaluate the robustness of our results. In all, 121 studies out of the 226 included (54%) were of high validity. Out of the 146 studies used in meta-analysis, 85 (58%) were of high validity. Study validity in our analysis may differ from study validity for the research question investigated by the authors. Interpretations of risk of bias judgements therefore only apply to the questions in this review.

#### Studies that reported measurements in adjacent soils

Although some studies sampled outside the restoration or drainage area, reporting of measurements outside the wetland soils was uncommon (n = 9 studies [49–57], marked in the “Measurements outside wetland” column in the metadata table). This group of studies was split between restoration (n = 5) and drainage (n = 3), with a single study on construction (n = 1). The studies were from the USA (n = 5), Sweden (n = 2), the UK (n = 1) and Ireland (n = 1). Unlike the rest of the evidence base, peatlands (n = 3) were not predominant. The remainder of studies were from limnic shore wetlands (n = 3), other types of wetlands (n = 2) or did not report the wetland type (n = 1).

The studies mostly had a study design with high risk of bias (n = 5 BA studies, n = 3 BACI, n = 1 CI), and only two studies [49, 53] were of high validity. Of these two, one study [49] focused on fish habitat and did not provide sufficient information to separate groundwater effects outside the wetland from those within the wetland. The other [53] investigated a single drained wetland, and reported drawdown of 5–20 cm in the mineral soil at distances up to 80 m from the fen.

Only two studies [52, 53] reported effects at different distances, although one additional study provided effect-distance information in map form [55]. These three studies were from different types of contexts (across a floodplain valley in the UK, in the mineral soil next to a Swedish peat fen, and in limestone-derived gravel next to an Atlantic raised bog in Ireland) and judged not comparable for meta-analysis.

In summary, the body of literature that reported groundwater measurements outside the wetland soil was limited and did not allow for a reliable evaluation of effects there. The answer to the question of effects outside the wetland soil was therefore *inconclusive*.

### Quantitative synthesis

We present results first for restoration and then for drainage. For both intervention types, we first present the relationship between change in groundwater level and distance from the intervention (based on the two effect sizes in the regression), followed by overall effects across the wetland. Constructed wetlands are grouped with restored wetlands. All data from meta-analyses are available in Additional files 7 and 8.

#### Restoration: relationship between change in groundwater level and distance from intervention.

Out of 115 non-redundant studies of restoration or construction, 14 studies were included in meta-analysis and reported measurements at distances up to 170 m from the intervention. Remaining studies were excluded for the following reasons: too short temporal coverage (17 studies had no data for at least one of the three summer months; see details in Additional file 6), no data for different distances from the intervention (73 studies), limnic shore wetlands that varied widely in context and were difficult to synthesize (9 studies; for example, distances to the intervention in several cases changed during the intervention, as stream channels were re-meandered or dried-out channels reconnected), or difficulty isolating the same distances for the control and treatment (2 studies).

**Fig. 8:**
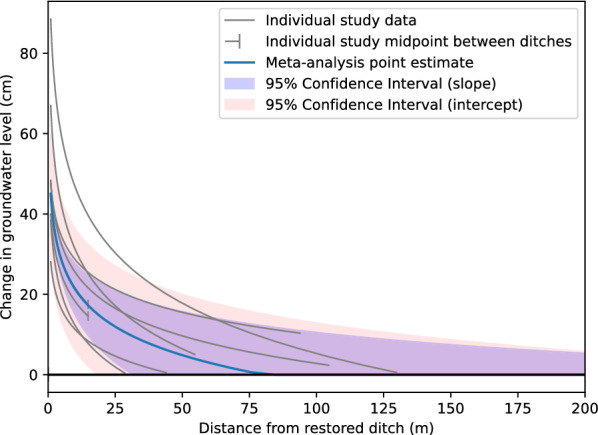
Peatland restoration effects at different distances (excluding blanket peatlands). Individual study (n = 8) and random effects (RE) model relationship y = m ln x + b between distance from restored ditch (x, measured in m) and change in groundwater level (y, measured in cm). Each gray line corresponds to the relationship established from reported effects in an individual study. Vertical bars indicate end points for transects that are between ditches, typically coinciding with midpoints between ditches. The blue line is the RE model point estimate based on all studies. The purple band shows the 95% confidence interval of the RE model estimate of the slope parameter (m). The pink band shows the 95% confidence interval of the RE model estimate of the intercept (b) parameter

Results showed a large variation in the change of groundwater level, and how it depends on distance from the intervention. It was clear that blanket peatlands behaved differently, and we therefore return to treat them separately. For the remaining peatland studies (n = 8), Fig. 8 shows the relationship between change in groundwater level and distance.

The increase in ground water level at 1 m from the restoration intervention ranged from 25 to over 80 cm. The intersection with the x axis, the point at which the effect is zero, ranged from c. 30 to c. 130 m, considering only the distances over which groundwater level sampling took place (with extrapolation, the range extended to over 200 m). The point estimate of the meta-analysis indicated that effects of restoration approached zero at ~ 80 [95% confidence interval: 32, 407] m distance.

**Fig. 9:**
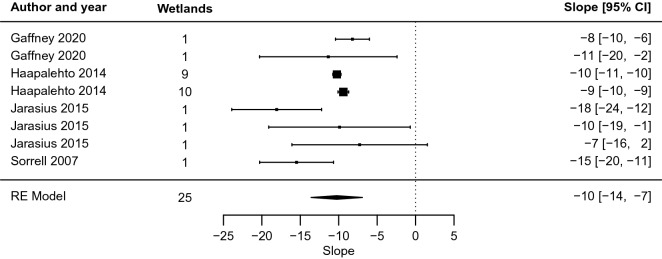
Forest plot of the slope coefficient m of the relationship y = m ln x + b between distance from restoration intervention (x, measured in m) and effect on groundwater level (y, measured in cm), for peatland studies (excluding blanket peatlands)

**Fig. 10:**
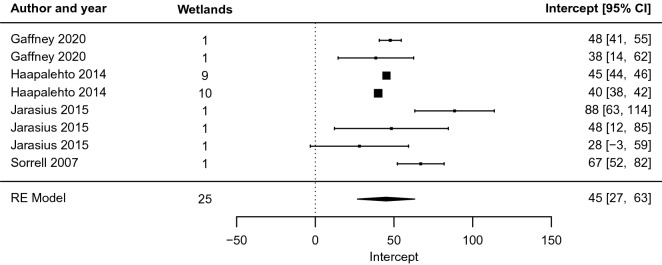
Forest plot of the intercept coefficient m of the relationship y = m ln x + b between distance from restoration intervention (x, measured in m) and effect on groundwater level (y, measured in cm), for peatland studies (excluding blanket peatlands)

Investigating the slope (*m*) and intercept (*b*) coefficients with their standard errors in a traditional forest plot (Fig. 9, Fig. 10) provides further details.

The slope corresponds to the change in groundwater level for a given unit change in the exponent of the base *e* in terms of distance (*e*^*1*^, *e*^*2*^, *e*^*3*^, …). For example, a slope value of − 10 means that the effect on groundwater level decreases by 10 cm when the distance changes from 1 to 2.7 (from *e*^*0*^ to *e*^*1*^) m, or from 2.7 to 7.4 m, and so on (~ 20 m, 55 m, and 148 m are the next integer exponentials of *e*). The value can also be interpreted through small (marginal) changes. For example, if the slope is 10, this means that a 1 per cent increase in distance will increase the groundwater level with 1 per cent of this value, i.e., 0.1 cm.

The intercept is rather straightforward to interpret. Since ln 1 equals zero, the intercept can be thought of as the effect, in centimeters, on the groundwater level at 1 m from the intervention. It also corresponds to an upwards or downwards shift of the curve that is constant across all distances, and thus represents overall “depth” or “intensity” of the effect, rather than how much it changes with distance.

For peatlands that were not blanket peatlands, the meta-analysis indicated an average rate of change of − 10 [− 14, − 7] cm for each unit change in distance from the ditch (Fig. 9). Thus, the effect on groundwater level decreased by about 10 cm with each doubling of the distance, in terms of exponents to the base *e*. In marginal terms, the effect of restoration on groundwater levels decreased by 0.1 cm for each increase in distance of 1 per cent. At a distance of 1 m from the ditch, the point estimate of the restoration effect is 45 [27, 63; Fig. 10] cm. The effect dropped off rather rapidly, and compared with the size at 1 m, it was halved at 9 [5, 26] m and only a quarter as large at 27 [12, 132] m.

**Fig. 11:**
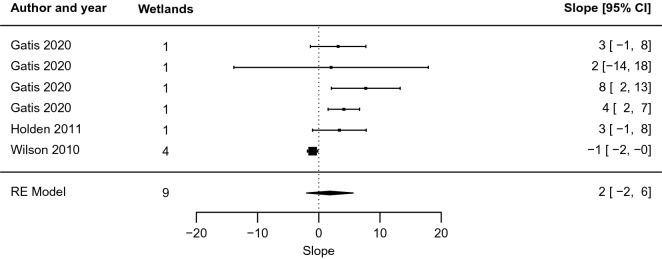
Forest plot for blanket peatlands only, showing the slope coefficient m of the relationship y = m ln x + b between distance from restoration intervention (x, measured in m) and effect on groundwater level (y, measured in cm)

**Fig. 12:**
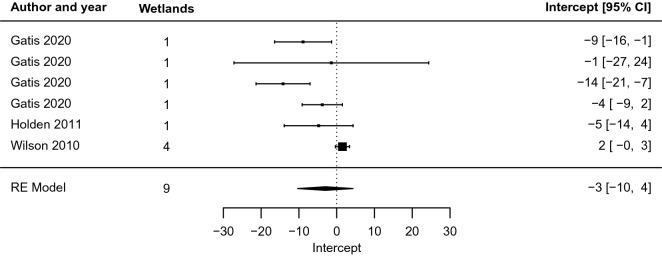
Forest plot for blanket peatlands only, showing the intercept coefficient b of the relationship y = m ln x + b between distance from restoration intervention (x, measured in m) and effect on groundwater level (y, measured in cm)

Turning to the blanket peatland studies (n = 6), the pattern was markedly different. For five of the studies, the best-fit regression implied effects that increased rather than decreased with distance over the sampled region (values of the slope coefficient *m* were positive, mean 2 [− 2, 6], Fig. 11). Both the slope (Fig. 11) and intercept coefficients (mean − 3 [− 10, 4], Fig. 12) were closer to zero than for other peatlands, meaning that effects did not change as quickly with distance and were of smaller overall magnitude. Inspecting the standard errors of the slope and intercept coefficients showed that, in relative terms, errors were four and nine times larger, respectively, than for the group of other peatlands. This means that an exponential function was a worse fit to the data for blanket peatlands than for other peatlands. As the confidence intervals of the random effects model overlapped the zero-effect line for both slope and intervention, effects were not statistically different from zero for restoration in blanket peatlands for the investigated studies.

We investigated the possible effects of peat depth, time since intervention, intervention magnitude, soil type, ditch spacing, transect type and climate zone as moderators, but none were significant. We were unable to test for the moderators wetland slope, intervention type and study design due to too few studies. Statistics of all moderators are provided in Additional files 7 and 8.

**Fig. 13:**
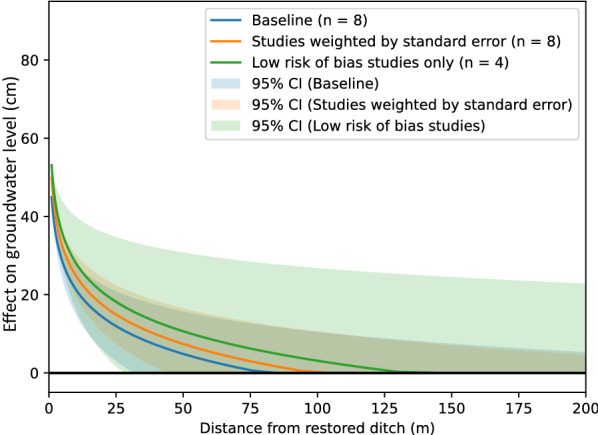
Sensitivity analysis of the random effects (RE) model relationship y = m ln x + b between distance from restored ditch (x, measured in m) and effect on groundwater level (y, measured in cm)

Sensitivity analyses showed no substantial differences in results when including only high validity studies (n = 4), or when we investigated effects of using inverse variance as study weights (Fig. 13 and Additional files 7 and 8).

#### Restoration: overall effects

**Fig. 14:**
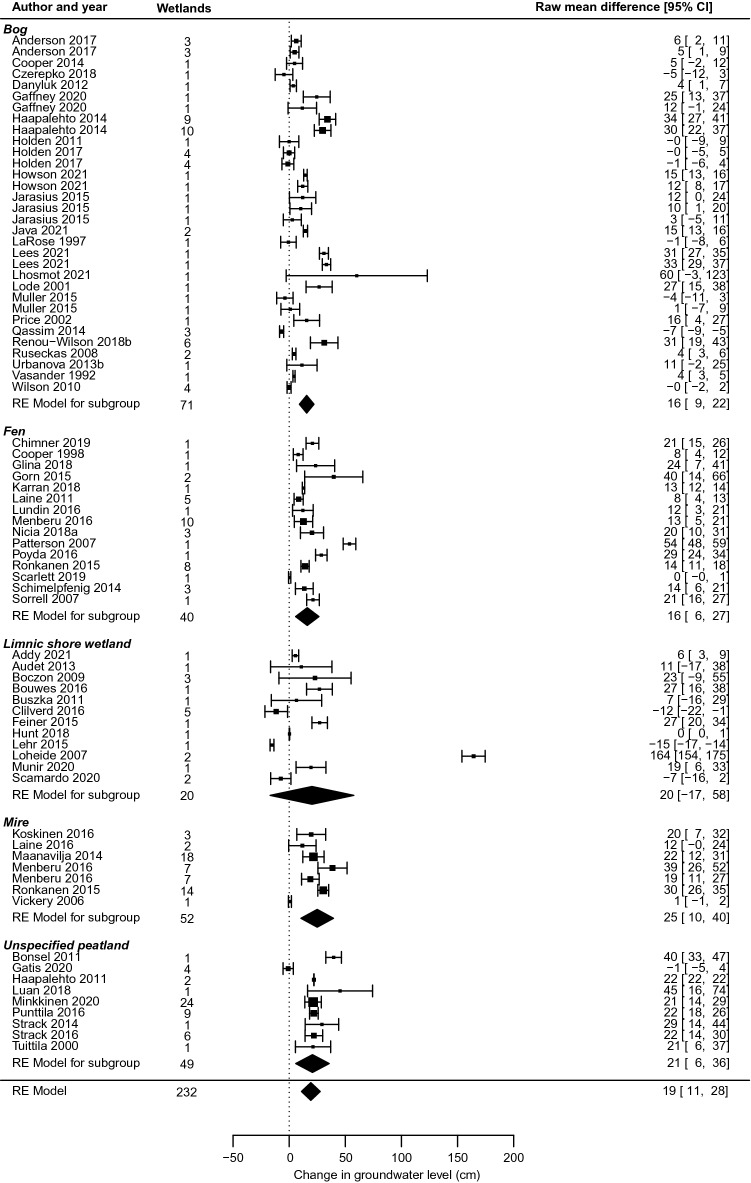
Forest plot of wetland restoration effects on groundwater level

Overall effects of wetland restoration were reported in 117 non-redundant studies in 102 articles. Out of these, meta-analysis was possible for 75 studies in 64 articles. A forest plot of effects, grouped by wetland type, is shown in Fig. 14. In the meta-analysis, the group “Unspecified peatland” contains both studies that did not report the peatland type and studies where a single treatment included several types of peatlands.

Notably, effects in different peatland groups were rather similar, with meta-analysis estimates of ground water level increases of c. 16, 16, 25 and 21 cm in bogs, fens, mires and unspecified peatlands, respectively (excluding blanket peatlands (n = 16), figures changed for both bogs and unspecified peatlands to 23 cm). For all peatlands grouped together (n = 63), the joint estimate was 19 [95% confidence interval 14, 25] cm (excluding blanket peatlands, 22 [16, 28] cm, n = 47). For no group of peatlands did the confidence interval overlap the zero-effect line.

For wetlands that were not peatlands, that is limnic shore wetlands, the picture was markedly different (Fig. 14). The confidence interval was wide and overlapped the zero-effect line. The group of limnic shore wetlands was diverse, both in effects and in local conditions around the wetland and the intervention. Six of the studies [49, 50, 56, 58–60] were from the USA, predominantly in high-elevation meadows. Clearly, effects in these types of wetlands ranged widely, from negative to positive. Remaining analyses of moderators and sensitivity therefore focused on peatlands.

Like effects evaluated at different distances, moderating factors were not significant (underlying soil type, peat depth, time since intervention, intervention magnitude, wetland slope, ditch distance, maximum distance of groundwater sampling, climate zone). For overall effects, we also investigated additional factors (wetland type, intervention type, replication type, study design) that were not significant. Statistics of all tests are available in Additional files 7 and 8.

Sensitivity analyses using inverse variance as study weights showed no significant changes, except that confidence intervals were somewhat narrower (overall effect 16 [11, 22] cm when including all wetlands (n = 75), changing to 18 [15, 22] cm (n = 47) when excluding blanket peatlands and limnic shore wetlands). Including only high validity studies showed similar values (overall effect 15 [7, 24] cm when including all wetlands (n = 42), changing to 15 [11, 19] cm (n = 27) when excluding blanket peatlands and limnic shore wetlands). or the limnic shore wetlands group, excluding the single outlier study at 164 cm showed a confidence interval that still overlapped the zero effect with substantial margins. Full statistics of all tests are available in Additional files 7 and 8.

#### Drainage: relationship between effect size and distance from intervention

**Fig. 15:**
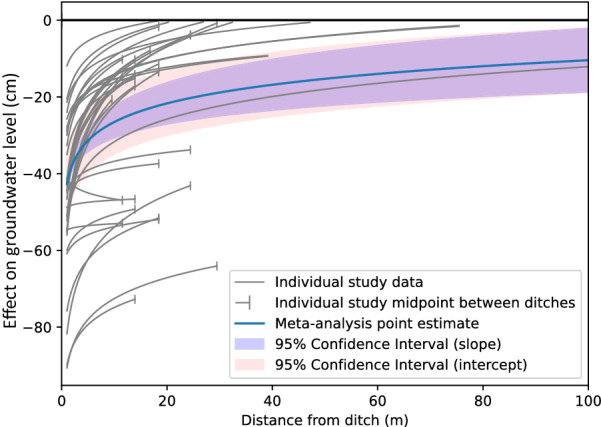
Peatland drainage effects at different distances (excluding blanket peatlands). Individual study and random effects (RE) model relationship y = m ln x + b between distance from ditch (x, measured in m) and effect on groundwater level (y, measured in cm). Each gray line corresponds to the relationship established from reported effects in an individual study. Vertical bars indicate end points for between-ditch transects, typically coinciding with midpoints between ditches. The blue line is the RE model point estimate based on all studies. The purple band shows the 95% confidence interval of the RE model estimate of the slope parameter (m). The pink band shows the 95% confidence interval of the RE model estimate of the intercept (b) parameter

After removing studies that were redundant or lacked sufficient temporal data, effects of drainage at different distances from the intervention (the ditch, in all cases) were reported in 41 studies in 17 articles. One study [61] did not report groundwater levels at different distances but other groundwater-related outcomes and was therefore excluded from the meta-analysis. Two studies [62, 63] presented data in map form that were difficult to extract. One study [53] investigated the effects outside the wetland in the adjacent soil and was therefore not included in meta-analysis. Another study [64] was from a blanket peatland. All remaining studies except one were carried out in peatlands, and that single study from another type of wetland ([52]; a floodplain in the UK) was therefore excluded from meta-analysis as it was judged to be not comparable to the others, leaving 35 studies. Like effects of restoration at different distances, studies showed a large variation in effect size and how it changed with distance from the intervention (Fig. 15). The change in ground water level at 1 m from the ditch ranged from − 10 to below − 90 cm.

The intersection with the *x* axis, the point at which the effect is zero, ranged from c. 20 m to over 225 m, considering only the distances over which groundwater level sampling took place. The point estimate of the meta-analysis indicated that effects of ditching became zero at about 440 m, but the confidence interval was very wide [95% confidence interval 121, 4191]. It is important to note that most studies did not sample farther than about 25 m from the ditch, and that a number of studies with deep ditches at close spacings are included. The estimates above are therefore largely based on how groundwater levels change close to the ditch, and the extension to a zero-effect distance is an extrapolation that is inherently uncertain.

**Fig. 16:**
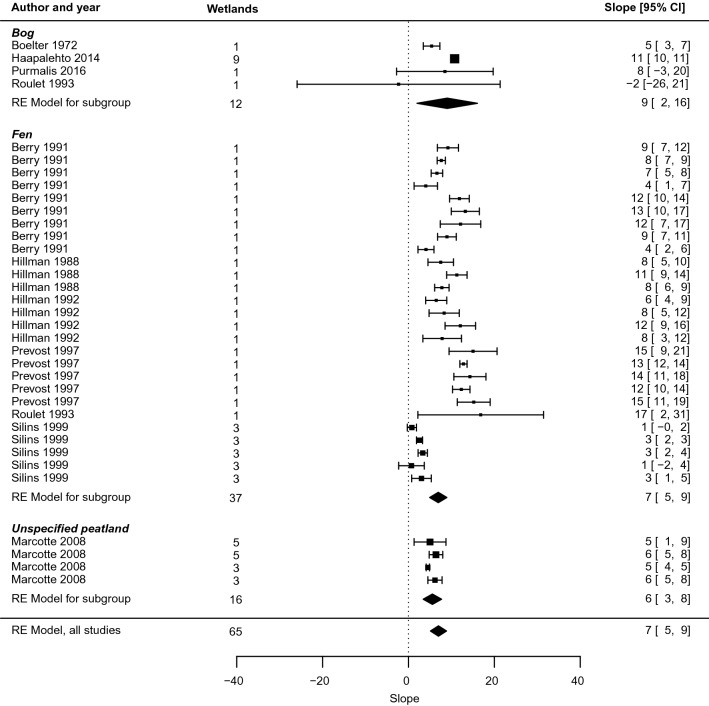
Forest plot of the slope coefficient m of the relationship y = m ln x + b between distance from drainage intervention (x, measured in m) and effect on groundwater level (y, measured in cm)

Compared with restoration, the meta-analysis confidence interval for drainage was narrower in absolute terms (SE = 1.4 for restoration vs 0.9 for drainage), but in relative terms they were similar (14% of the slope coefficient value in both cases). As with restoration, one study [65] had a best-fit regression that implied effects that were reversed from the expected direction and increased rather than decreased with distance (Fig. 16).

The group of studies was predominated by investigations in fens, comprising 27 out of the 35 studies included in the meta-analysis. With the exception of [65], slopes were distinctively positive in the RE model for all studies combined and in all subgroups, save bogs for which the confidence interval lower bound was close to a zero effect.

**Fig. 17:**
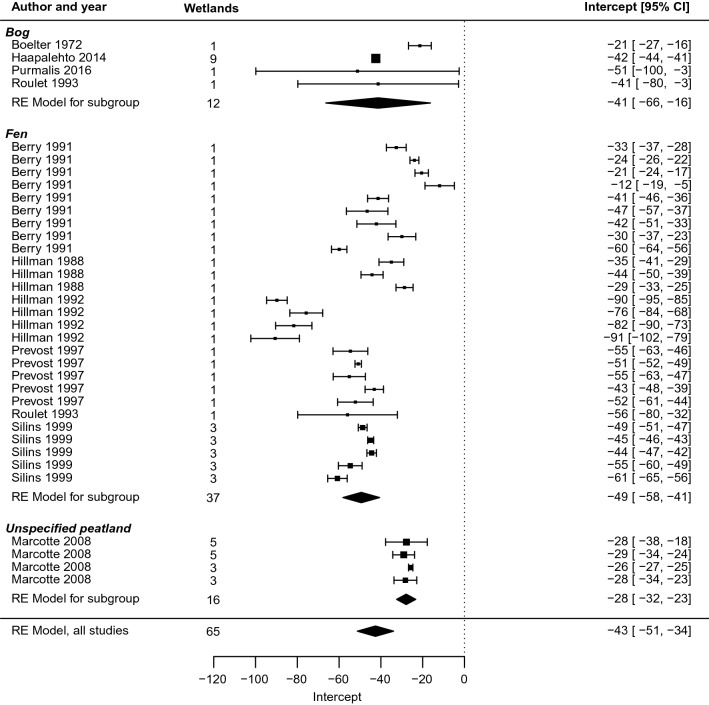
Forest plot of the intercept coefficient b of the relationship y = m ln x + b between distance from drainage intervention (x, measured in m) and effect on groundwater level (y, measured in cm)

For all wetlands, the meta-analysis indicated an average rate of change of 7 [5, 9] cm for each unit change in distance from the ditch. Thus, the effect on groundwater level decreased by between about 5 and 9 cm with each doubling of the distance, in terms of exponents to the base *e*. In marginal terms, the effect of drainage on groundwater levels decreased by 0.07 cm for each increase in distance of 1 per cent. At 1 m from the ditch, the point estimate of the drainage effect was − 43 [− 51, − 34] cm (Fig. 17). Compared with restoration, the effect dropped off more slowly: compared with the size at 1 m, it was halved at 21 [11, 64] m and a quarter as large at 97 [37, 514] m.

**Fig. 18:**
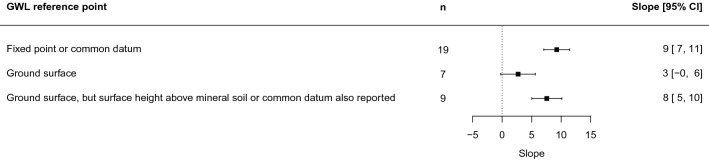
Forest plot of the slope coefficient m of the relationship y = m ln x + b between distance from drainage intervention (x, measured in m) and effect on groundwater level (y, measured in cm), separated between studies that measure the groundwater level in different ways. Blanket peatland studies are not included

Like restoration, we tested moderators and found mostly no significant effects (wetland type, ditch spacing, wetland slope, peat depth, time since intervention, intervention magnitude, transect type, climate zone). However, the groundwater level reference point was a significant moderator (*p* = 0.005, Fig. 18) for the slope parameter (*m*), with studies measuring against a fixed point or common datum showing greater change of the effect with distance than studies measuring against the ground level only. Also, the intercept (*b* parameter) was shallower at open transects compared to closed ones (see Fig. 14 in Additional file 8).

**Fig. 19:**
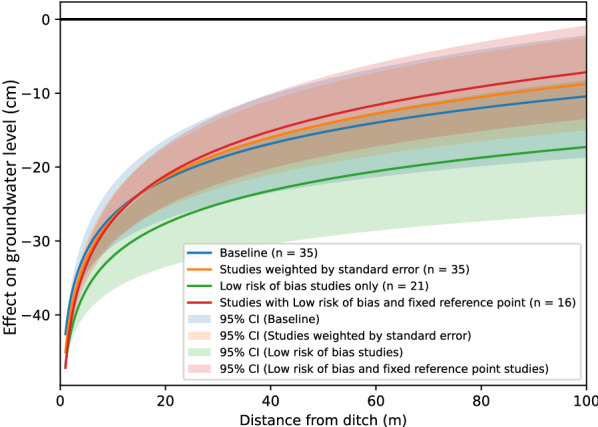
Sensitivity analysis of the random effects (RE) model relationship y = m ln x + b between distance from ditch (x, measured in m) and effect on groundwater level (y, measured in cm)

Sensitivity tests showed no substantial differences close to the ditch when weighting studies by inverse variance, or when only including studies with low risk of bias (Fig. 19). Differences were larger at greater distances from the ditch, particularly for the group of studies with low risk of bias. However, a group of studies with low risk of bias and the more reliable fixed reference point was again close to the baseline model specification. All statistics of tests of moderators and sensitivity tests are provided in Additional files 7 and 8.

#### Drainage: overall effects

**Fig. 20:**
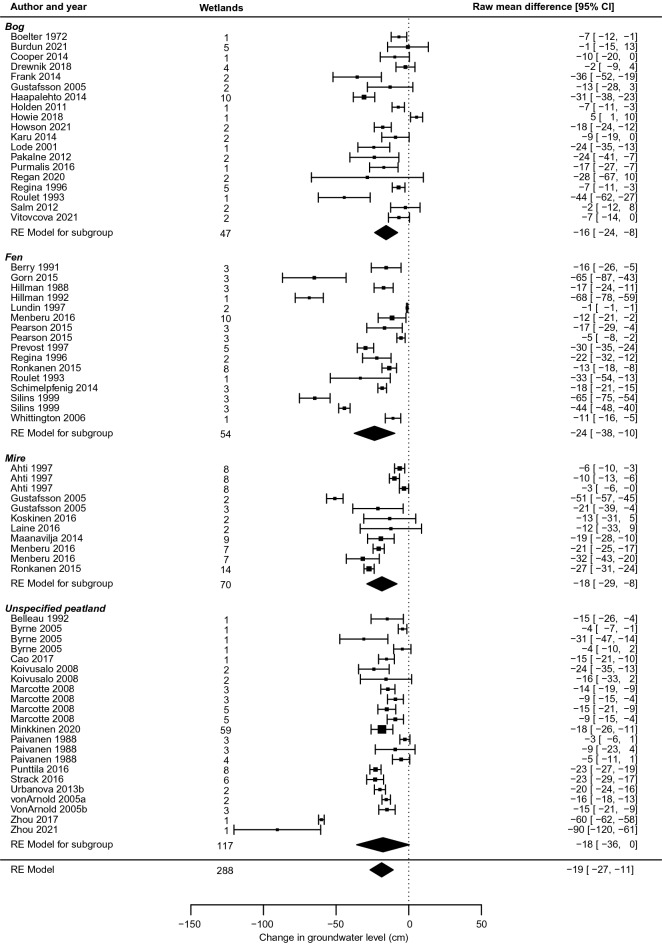
Forest plot of wetland drainage effects on groundwater level

The evidence base of overall effects of wetland drainage comprised 114 non-redundant studies in 94 articles. Out of these, 70 studies in 51 articles were possible to include in meta-analysis. However, one study was excluded as it was the only non-peatland study of drainage effects [52], and one study was excluded as it investigated drainage of soil outside the wetland [53]. A forest plot of effects in the remaining 68 studies, grouped by wetland type, is shown in Fig. 20.

For drainage, the confidence interval did not overlap with zero except for unspecified peatlands, and then only just, due to a single study with very large effects that widened the overall confidence interval. Effects differed substantially between studies, particularly for bogs and fens, but group effects indicated that differences between wetland types were small. Effects in bogs, mires and unspecified peatlands differed on the group level by only 2 cm, while fens showed a value that was ~ 5 cm lower than any other group.

Overall, the effects of drainage when combining all studies were close to mirroring the effects of restoration (change of − 19 [95% confidence interval − 27; − 11] cm for drainage, compared to + 19 [11, 28] cm for restoration). If we restricted the comparison to peatlands (there were only peatlands in the drainage meta-analysis), the difference was similar, but more precisely estimated for restoration (restoration effect of + 19 [14, 25] cm). If we excluded studies on blanket peatlands (n = 6), results changed to − 19 [− 27, − 10] cm for remaining drainage studies (n = 62). Corresponding restoration effects here differed somewhat (+ 22 [16, 28] cm, n = 47) but confidence intervals still largely mirrored each other.

**Fig. 21:**
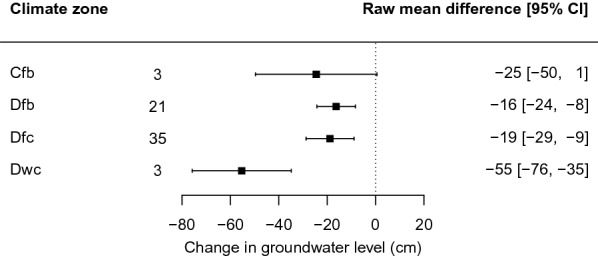
Forest plot of the influence of the climate zone on the change in groundwater level after drainage

We investigated several moderators, most of which were not significant (wetland type, peat depth, time since intervention, underlying soil type, intervention magnitude, ditch spacing, wetland slope, maximum distance of groundwater level sampling, intervention type, replication type, study design, groundwater reference level). The only significant moderator was the climate zone, indicating that studies in climate zone Dwc (n = 3, including three high altitude peatlands in China) showed significantly different effects than studies in zones *Dfb* and *Dfc* (Fig. 21). As for restoration, the deviant group was small, and the *Dfb* and *Dfc* zones were predominant. Test statistics are available in Additional files 7 and 8.

We also investigated effects of ditch network maintenance (DNM) by separately analyzing the DNM articles. Even when only considering articles evaluating DNM, no significant effect of intervention type was present (that is, ordinary ditching could not be separated from DNM in terms of groundwater level effects). Using a multilevel model with clustering of studies by article still showed no significant effect of the type of intervention. DNM studies were very few, however (n = 8).

Sensitivity analyses showed similar effects to our baseline specification. Excluding blanket peatlands, the overall effect was − 20 [− 24, − 16] cm when using inverse study variance as weights instead of sample sizes. All statistical details of tests of moderators and sensitivity tests are provided in Additional files 7 and 8.

#### Synthesis of overall effects across both restoration and drainage

A persistent result for peatlands was that the restoration effect was a mirror of the drainage effect, in terms of magnitude when estimated with meta-analysis across studies. This implies that restoration can be expected to reverse drainage effects, at least in terms of an average magnitude of local ground water level change over groups of wetlands, irrespective of the type of wetland in this geographical region.

In environmental systems, large heterogeneity in effects is a typical phenomenon [66–68]. Identifying sources of this heterogeneity is an important way that systematic reviews can contribute to knowledge in the field. In our analysis, we saw large differences in effects for individual wetlands and studies (as expected), but were not able to isolate relevant moderators that had a significant and practically important influence on effect sizes. For some moderators, the variation among studies in our sample may be small, with studies clustered within a small range, which makes it difficult to capture the influence a moderator could have. It is important to note that even when we find no significance for various moderating factors, this cannot be interpreted as evidence of no effect unless the remaining amount of heterogeneity is small. This was not the case for the meta-analyses or tests of moderators we investigated. The heterogeneity statistics are reported in detail in Additional File 7, with further discussions in Additional File 8.

**Fig. 22:**
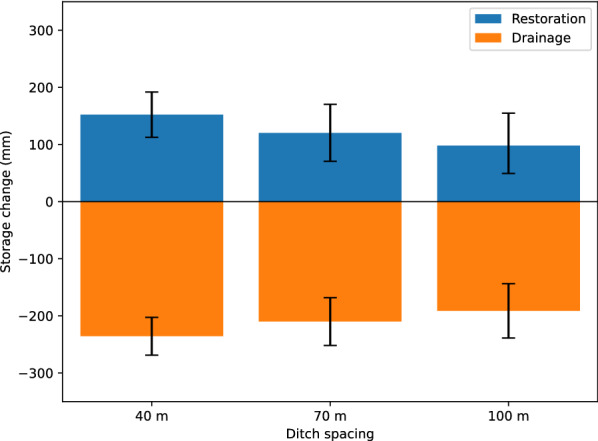
Area-wide groundwater storage change in mm for restoration and drainage, based on the relationship between effect size and distance (Fig. 8 and Fig. 15) and ditches spaced at different intervals. Error bars show confidence interval estimates based on the slope parameter for effects reported at different distances from the ditch

To put the results in context, we calculated the changed groundwater storage in the wetland soil for various typical ditch spacings (Fig. 22). With ditches more densely spaced, groundwater storage effects increased. Drainage effects were larger than restoration effects, in terms of actual groundwater stored, partly due to a more extensive lateral effect and partly due to our assumption of higher porosity in undrained soils. Without the assumption of decreasing porosity, differences are smaller but remain (see Additional File 8). The magnitude of the effect was substantial for both restoration and drainage when compared to typical annual precipitation in boreal regions (on the order of 500–1000 mm/year). Despite the large uncertainty in the confidence interval estimate of the distance to no effect, confidence interval estimates were rather narrow for the storage volume change. This is because only the first 20, 35, and 50 m of the curve, respectively, were used to calculate effects at the different ditch spacings. Since the confidence interval estimates of the no effect distance were mostly greater than those distances, there was a relatively small effect on the storage volume closer to the ditch.

#### Comparison with findings in the literature

Our results are mostly in line with previously published results and reviews. A review by Lindsay et al. [10] noted that the effect of drainage can extend to well over 100 m, which generally is in line with our analysis. However, Lindsay noted that sampling from the ground should yield shallower depths of water table drawdown compared to sampling from a fixed reference point well anchored in bedrock or mineral soil. In our analysis, however, we could not isolate a clear influence of sampling level on the results. We tested for the reference level for groundwater measurements as a moderator, but it was not significant, except for drainage effects measured at different distances.

A study by Ameli and Creed [69] has found that wetland loss increased groundwater travel times and decreased groundwater discharge areas. This is in line with our findings of decreased groundwater levels, but it is not immediately intuitive in terms of lowered groundwater levels away from the ditch. In their analysis, shrinking groundwater catchments from wetland loss decreased the discharge to local water bodies, which could increase risks of water shortage if other water withdrawals occur nearby [69]. However, risks are greater where aquifers are thick with small variability in conductivity. The risks in heterogeneous aquifers, such as glaciofluvial deposits on top of impermeable bedrocks, is smaller according to their study [69]. In Swedish overview reports [13, 29], effects are mostly stated in qualitative terms. Examples of results from individual studies, included in this review, are presented, but there is no summary value of how far the effect extends or how much the groundwater level will change on average that can be compared with estimates in this review. However, a modeling study by the Swedish geological survey [70] quantified restoration effects at different distances for a few typical hydrogeological settings. The results indicate that effects are limited to the few dozen meters closest to the intervention, except where soils next to the wetland are conductive, where effects can extend to several hundred meters in favorable conditions.

Results in the Bussell et al. review [40] are largely compatible with our findings. Their meta-analysis included 26 drainage studies in peatlands and their point estimate and confidence intervals (− 16 [− 22, − 11] cm) are close to our corresponding figures (− 19 [− 27; − 11] cm). Model simulations, presented in another review by Price et al. [43], show curves of water table drawdown after drainage and subsequent increase of water levels after dich blocking. Like our results, effects in the simulation fit well to a logarithmic relationship (R^2^ = 0.94–0.99). In this analysis, based on assumptions of a peat depth of 1 m with relatively impermeable subsoil, the restoration effect of 34 cm at 1 m from the ditch is similar to our estimate of 45 [27, 63] cm. However, in the simulation, the effect was halved already at 3 m from the ditch, which is short compared to our finding of 9 [5, 26] m.

#### Sensitivity of findings to publication bias

We used funnel plots and Egger’s tests to investigate a tendency for publication bias in the literature. All individual effect sizes in the four main meta-analyses were plotted against sample standard error. Following recommendations [71, 72], we also used the trim and fill method [73, 74] to investigate what the effect on our results could be if publication bias were present. Results are presented in Additional file 9, and although publication bias cannot be ruled out, there is no clear indication of publication bias nor substantial impact of it for any of the meta-analyses. In addition, cumulative forest plots (Figs. 9 and 17 in Additional file 8) show stability of effect sizes over time as new studies are added.

### Review limitations

Due to resource constraints, several steps of our review process (abstract screening, data extraction, study validity assessment) were not done independently by two reviewers. When we tested dual reviewers on subsets of articles or studies, agreement was good but not perfect. It is therefore likely that a full dual review of all items would have produced slightly different decisions for some articles, studies, or data. However, the level of agreement was relatively high, and we aimed to err on the side of caution for inclusion or exclusion decisions. Despite possible errors or misclassifications, we judge it unlikely that these would substantially alter any conclusions.

We attempted to perform a comprehensive search by including many common words to refer to wetlands and groundwater, but as our search only retrieved references with these words in the title, abstract or keywords, we may have missed studies that either did not use any of these words or used them but only in the main body of the text. It is difficult to assess the influence of this risk of bias, but we expect it to be of limited concern. One reason is that studies that focused on groundwater effects, with more useful reporting for our purposes, should have been more likely to mention groundwater terms. Also, we have no reason to believe there was a systematic bias in effects between studies that mentioned groundwater information only in passing and studies that reported groundwater effects as a key result.

It can be questioned whether different wetland studies are comparable. Wetlands are inherently diverse systems, and many factors can influence local conditions and effects. Even though we have judged at least the peatland interventions to be comparable, results combining different wetlands should be interpreted with caution.

The statistical tests we used require that variability was independent in all studies. This assumption may have been violated as some studies included comparison of multiple intervention sites against a single control site. However, in all studies, there was only a single outcome (groundwater level) used in each meta-analysis, and many studies used paired designs with independent controls for each intervention site. Overall, however, it is likely that certain non-independent controls contributed to a somewhat narrower confidence interval than would otherwise be the case. The statistical model in this study, as in most other empirical research in the environmental sciences, cannot be interpreted as fully covering all sources of uncertainty.

For the meta-analyses of studies without reported distances to the intervention, the results can be considered an average of areas that researchers were interested in. We judge it highly likely that groundwater sampling locations in these studies were skewed towards points closer to the intervention than points in the wetland as average. Therefore, our estimates likely represent an effect size near the intervention, rather than the whole wetland. This should be kept in mind when interpreting results.

Although we have no empirical basis for this judgement, the extent of peatland drainage in Russia, together with a historically limited volume of international scientific publication there, points to a potential gap from not including studies in Russian. However, the omission of Russian and other languages, for example in Eastern Europe, only contributes to a biased estimate if effects in those regions were relevant but systematically different from the remaining sampled literature.

We have included gray literature in our synthesis and not treated these studies differently than traditional academic peer-reviewed literature. However, critical appraisal was carried out in the same way for both kinds of literature, and we therefore expect any bias from including gray literature to be limited.

### Limitations from the evidence base

For effects outside the wetland, the main limitation was that there were few studies investigating this. This means that we had little empirical basis for evaluating effects of wetland interventions in terms of any influence to groundwater outside the restored or drained area.

Compared with peatland studies, we found few studies on limnic shore wetlands that fulfilled our criteria. This points to a possible research gap. It was common with experimental peatland studies that were designed to evaluate the effect on groundwater. In contrast, studies on limnic shore wetlands tended to be process understanding studies, or focused on other aspects than groundwater. Few involved a control that allowed the comparison of groundwater effects with or without the intervention. Instead, instrumentation was often restricted to the actual intervention site and time. A notable exception was beaver dam studies in North America. In several cases, these were designed with control-intervention sites, sometimes also with a comparison period before the intervention.

More generally, and in terms of study reliability, short calibration and intervention periods were not uncommon and may have increased the risk of bias due to weather variability. At least a few years would be desirable in before-after comparisons [75]. Long monitoring periods are however often not possible with project-based research funding a few years at a time. This affects not only research projects—environmental management projects also tend to have limited budgets for monitoring of long-term effects.

Most studies had small samples, with n = 1 wetland the most common. This meant that there was no variability estimate of the intervention effect, other than pseudoreplication within the same treated site. To increase knowledge about the variability of treatment effects, it would be helpful with fewer but larger studies, where treatments are replicated with the same method but in independent wetlands. Granted, such study designs are more costly and would require pooling resources, and judgement is needed as to what research designs best serve the need for science and policy considering multiple goals.

For restoration, the time since intervention was on average shorter than for drainage studies. We did not find any significant effect of the time since intervention, however, but it would be possible to investigate this in more detail with a larger spread among the studies.

## Review Conclusions

This review aimed to evaluate all available empirical studies on effects of groundwater storage from restoring, constructing, and draining wetlands in temperate and boreal climates. We were not able to evaluate the potential effects outside the wetland soil. Those results were inconclusive due to a small evidence base. This implies that more research is needed to provide a solid answer about what effects outside the wetland can be expected. For managers overseeing wetland projects, this means that projects that aim to reinforce groundwater storage in soils adjacent to the wetland should consider initial implementation at pilot scales or additional resources for monitoring and following up on effects.

We were able to answer the secondary question on how far effects extended from the intervention, and its overall size, as well as the further secondary questions on factors that influenced the size of the effect. Restoring or draining peatlands (except blanket bogs) had substantial effects on the groundwater level in the vicinity of ditches. The relationship between the change in groundwater level and distance from ditch was well described by an exponential function. The average effect on groundwater levels in the vicinity of restoration or drainage was + 22 [95% confidence interval 16, 28] cm and − 19 [− 27, − 10] cm, respectively, in peatlands that were not blanket bogs. This conclusion is conditional on measurements being performed in the vicinity of the restoration or drainage.

At a distance of 1 m from a ditch in peatlands, the groundwater level increased by 45 [27, 63] cm after restoration and fell by − 42 [− 51, − 33] cm after drainage. The effect was reduced to 50% after 9 [5, 26] m for restoration and 21 [11, 64] m for drainage, and to 25% after 27 [12, 132] m for restoration and 97 [37, 514] m for drainage. These figures apply to relatively flat peatlands and interventions in ditches.

The effect of restoration and drainage did not differ between different types of peatlands (bogs, fens and mires). The conclusion applies at group level and is conditional on sampling not being systematically biased between different types of wetlands. The effect of restoration and drainage was heterogeneous, however, and differed between different wetland sites, even for similar depths of restoration or drainage interventions. Restoration of blanket bogs had small or no effects on groundwater levels. Results for limnic shore wetlands and other types of wetlands were inconclusive.

### Implications for policy and management

In favorable hydrogeological conditions, such as where sandy soils are adjacent to a wetland, there are reasons to believe that effects could extend into surrounding soils, but we have not found a solid evidence-base of such investigations. Wetland project managers and decisionmakers with the specific aim of changing groundwater storage in adjacent soils should therefore consider reinforced efforts to monitor and follow-up effects outside the wetland. Projects should focus on interventions with permeable soils next to the wetland. Starting with pilot studies or small-scale projects can also be a valid strategy when effects are uncertain.

Our results indicate that intervention magnitude is not a significant factor affecting the outcome of wetland restoration or drainage. This points to context-dependent effects that may be hard to predict with any precision. In practice, a similar depth of restored ditch can be associated with varying effects on the groundwater level, depending on other local factors that also influence the outcome. If faced with two proposed wetland restoration projects, similar in approach and magnitude, it is important to realize that the resulting changes in groundwater level may differ. To the degree that effect modifiers can predict outcomes, we have not been able to isolate them, except the decreasing effect with increasing distance from the ditch. Wetland interventions may therefore need to incorporate some margin of error in the design if specific amounts of groundwater level change are desired. The distance to half of the effect, however, is a useful rule to estimate the extent of changes that can be expected. In practice, it puts a typical limit of a few tens of meters for effects that are of substantial magnitude, although effects may extend farther under favorable conditions.

Drainage effects extended farther than restoration. In practice, this means that past drainage may extend beyond the reach of restoration, even if the restoration is successful close to the ditch in terms of groundwater levels. One reason for this difference could be related to the compaction of peat soil after drainage. Even if the compaction is greatest near the ditch, effects can extend out to 100 m or farther, as noted by Lindsay et al. [10]. This difference in the extent of the effect calls for some caution in our other finding that restoration effects mirror the drainage, and that restoration therefore fully restores the effect of drainage in terms of groundwater levels. While this is likely close to the ditch, it is less certain if groundwater levels, or stored groundwater amounts, will fully recover farther away.

With heterogeneous effects, managers can use the list of studies to investigate cases that were closer in context to their respective situation. From a Swedish perspective, some results may be less transferable as ditches are typically dug deeper in Finland and Canada than in Sweden.

### Implications for research

There were few studies that included effects on groundwater in adjacent soils. In terms of both research and practical planning, alternative methods may then be needed, such as evaluating whether any wetland effect is discernible in other outcomes, like surface water levels, stream discharge, or recession flow patterns. Due to the identified knowledge gaps, there is a need for studies that empirically quantify effects of wetland restoration, construction, and drainage outside the wetland, in places where such effects can be expected to be of importance, such as in sandy soils.

In ecology and related subjects, research studies are often lacking in robust design [76–79]. While it may be difficult to use fully randomized designs in hydrology, several studies showed that it is possible to achieve true replication with a reasonable number of sites. Of course, it depends on the focus of a particular study whether it is feasible, as some measurements are easier to perform across many wetlands while other may be unreasonably time-consuming or expensive.

In many studies where a variability estimate was provided (often in the form of standard error or standard deviation), it was not reported what this estimate was based on. The reader is left to guess as to whether it was based on all samples, plot averages, or something else. In many cases the *N* was omitted, precluding the use of the variability estimate in meta-analysis and obscuring the basis for the calculation. A simple improvement, one that should be considered a requirement by editorial boards and reviewers, would be to always report the *N* and the underlying unit of analysis that is the basis for variance estimates given as error margins ( ± ) or ranges around a central value. Also, there is often a lack of systematic reporting of potential effect modifiers (soil conditions, peat depth, wetland area, ditch depth), which is another topic to address in future research.

## Supplementary Information

**Additional file 1.** ROSES reporting standards.

**Additional file 2.** Searches for literature.

**Additional file 3.** List of articles excluded at full text screening and reasons for exclusion.

**Additional file 4.** List of included articles.

**Additional file 5.** Metadata spreadsheet including coding instructions.

**Additional file 6.** Details on data extraction strategy.

**Additional file 7.** Results of all meta-analyses, moderator tests and sensitivity analyses.

**Additional file 8.** Discussion of moderator tests and sensitivity analyses.

**Additional file 9.** Funnel plots.

**Additional file 10.** Meta-analysis code in R.

## Data Availability

The datasets supporting the conclusions of this article are included within the article and its additional files.
