# Supplementary material for: Effects on groundwater storage of restoring, constructing or draining wetlands in temperate and boreal climates: a systematic review
Source: Environ Evid. 2022 Dec 8;11:38. doi: 10.1186/s13750-022-00289-5 (PMC11378806; doi:10.1186/s13750-022-00289-5)
Supplement: Supplementary file 2 — Additional file 2. Searches for literature. [file 13750_2022_289_MOESM2_ESM.docx]

README

Title: Searching for literature

Description: This additional file describes all the search strings that was used to find peer-reviewed articles and grey literature to include in the systematic review. Searches were made in bibliographic databases, an academic search engine and websites of relevant organizations.

**Searching for literature**

**Bibliographic database search**

**Database: Scopus**

Database provider: Elsevier

Date of search: December 10, 2021

| **No** | **Search string** | **Number of hits** |
| --- | --- | --- |
|  | **Intervention: Restoration, construction or drainage of wetlands** |  |
| **1** | TITLE-ABS-KEY((restor* OR construct* OR creat* OR manmade OR "man* made" OR rehabilit* OR inundat* OR rewet* OR "re-wet*" OR "clear cut*" OR dam OR dams OR damming OR "re-meander*" OR remeander* OR "grip block*" OR gripblock* OR "re-establish*" OR "re-vegetation" OR revegetation OR "re-vegetating" OR revegetating OR "vegetation removal" OR "remov* vegetation" OR "removal of vegetation" OR excavation OR excavating OR backfill* OR drain* OR ditch* OR "dry* out" OR "dried out") **W/10** (wetland* OR "wet* land*" OR bog OR bogs OR bogland* OR carr OR carrs OR fen OR fens OR fenland* OR "flood plain*" OR floodplain* OR "river plain*" OR riverplain* OR marsh* OR mire* OR "peat land*" OR peatland* OR peatbog* OR pond* OR moor* OR highmoor* OR lowmoor* OR riparian* OR riverine* OR "river bank*" OR swamp* OR shore* OR aapa* OR histosol* OR morass* OR muskeg* OR quag* OR slough* OR "flood* meadow*" OR "wet* meadow*" OR "flood* forest*" OR "wet* forest*" OR "flood* grassland*" OR "wet* grassland*" OR "flood* heath*" OR "wet* heath*" OR "overflow zone*" OR "over flow zone*" OR "overflow area*" OR "over flow area*")) | **51 519** |
|  | **Outcome: Change in groundwater level, storage or amount** |  |
| **2** | TITLE-ABS-KEY(groundwater* OR "ground water*" OR "water table*" OR watertable* OR "water budget*" OR waterbudget* OR "water storag*" OR waterstorag* OR "hydraulic head" OR "pump* test*" OR "subsurface hydrolog*" OR "sub surface hydrolog*" OR piezometer* OR "observation well*" OR "monitoring well*" OR "water well*" OR "dip well*" OR dipwell* OR "bore hole*" OR borehole*) | **335 695** |
|  | **Combination of search strings** |  |
| **3** | 1 AND 2 | **5 389** |
|  | **Limit to language: English, Danish, French, German, Norwegian, Polish, Swedish** |  |
| **4** | AND (LIMIT-TO(LANGUAGE, "English”) OR LIMIT-TO(LANGUAGE, "Danish”) OR LIMIT-TO(LANGUAGE, "French”) OR LIMIT-TO(LANGUAGE, "German”) OR LIMIT-TO(LANGUAGE, "Norwegian”) OR LIMIT-TO(LANGUAGE, "Polish”) OR LIMIT-TO( LANGUAGE, "Swedish”)) | **5 198** |

* = Represents any group of characters, including no character

" " = Searches for an exact phrase

TITLE-ABS-KEY = Title or Abstract or Keywords

W/10 = A proximity operator to find terms within ten words from each other

**Database: Web of Science Core Collection (1970-)**

Database provider: Clarivate Analytics

Date of search: December 10, 2021

Including: Science Citation Index Expanded (SCI-EXPANDED), Social Sciences Citation Index (SSCI), Arts & Humanities Citation Index (A&HCI), Conference Proceedings Citation Index- Science (CPCI-S), Conference Proceedings Citation Index- Social Science & Humanities (CPCI-SSH) and Emerging Sources Citation Index (ESCI)

| **No** | **Search string** | **Number of hits** |
| --- | --- | --- |
|  | **Intervention: Restoration, construction or drainage of wetlands** |  |
| **1** | TS=((restor* OR construct* OR creat* OR manmade OR "man* made" OR rehabilit* OR inundat* OR rewet* OR "re-wet*" OR "clear cut*" OR dam OR dams OR damming OR "re-meander*" OR remeander* OR "grip block*" OR gripblock* OR "re-establish*" OR "re-vegetation" OR revegetation OR "re-vegetating" OR revegetating OR "vegetation removal" OR "remov* vegetation" OR "removal of vegetation" OR excavation OR excavating OR backfill* OR drain* OR ditch* OR "dry* out" OR "dried out") **NEAR/10** (wetland* OR "wet* land*" OR bog OR bogs OR bogland* OR carr OR carrs OR fen OR fens OR fenland* OR "flood plain*" OR floodplain* OR "river plain*" OR riverplain* OR marsh* OR mire* OR "peat land*" OR peatland* OR peatbog* OR pond* OR moor* OR highmoor* OR lowmoor* OR riparian* OR riverine* OR "river bank*" OR swamp* OR shore* OR aapa* OR histosol* OR morass* OR muskeg* OR quag* OR slough* OR "flood* meadow*" OR "wet* meadow*" OR "flood* forest*" OR "wet* forest*" OR "flood* grassland*" OR "wet* grassland*" OR "flood* heath*" OR "wet* heath*" OR "overflow zone*" OR "over flow zone*" OR "overflow area*" OR "over flow area*")) | **39 785** |
|  | **Outcome: Change in groundwater level, storage or amount** |  |
| **2** | TS=(groundwater* OR "ground water*" OR "water table*" OR watertable* OR "water budget*" OR waterbudget* OR "water storag*" OR waterstorag* OR "hydraulic head" OR "pump* test*" OR "subsurface hydrolog*" OR "sub surface hydrolog*" OR piezometer* OR "observation well*" OR "monitoring well*" OR "water well*" OR "dip well*" OR dipwell* OR "bore hole*" OR borehole*) | **211 991** |
|  | **Combination of search strings** |  |
| **3** | 1 AND 2 | **4 322** |
|  | **Limit to language: English, Danish, French, German, Norwegian, Polish, Swedish** |  |
| **4** | AND LANGUAGE: (English OR Danish OR French OR German OR Norwegian OR Polish OR Swedish) | **4 297** |

* = Represents any group of characters, including no character

" " = Searches for an exact phrase

TS = Topic Search (search the Title, Abstract, Author Keywords and Keywords Plus within every record)

NEAR/10 = A proximity operator to find terms within ten words from each other

**Database: Academic Search Premier**

Database provider: EBSCO

Date of search: December 10, 2021
Search mode: Boolean/Phrase
Expanders: Apply equivalent subjects

| **No** | **Search string** | **Number of hits** |
| --- | --- | --- |
|  | **Intervention: Restoration, construction or drainage of wetlands** |  |
| **1** | (**SU** ((restor* OR construct* OR creat* OR manmade OR "man* made" OR rehabilit* OR inundat* OR rewet* OR "re-wet*" OR "clear cut*" OR dam OR dams OR damming OR "re-meander*" OR remeander* OR "grip block*" OR gripblock* OR "re-establish*" OR "re-vegetation" OR revegetation OR "re-vegetating" OR revegetating OR "vegetation removal" OR "remov* vegetation" OR "removal of vegetation" OR excavation OR excavating OR backfill* OR drain* OR ditch* OR "dry* out" OR "dried out") **N10** (wetland* OR "wet* land*" OR bog OR bogs OR bogland* OR carr OR carrs OR fen OR fens OR fenland* OR "flood plain*" OR floodplain* OR "river plain*" OR riverplain* OR marsh* OR mire* OR "peat land*" OR peatland* OR peatbog* OR pond* OR moor* OR highmoor* OR lowmoor* OR riparian* OR riverine* OR "river bank*" OR swamp* OR shore* OR aapa* OR histosol* OR morass* OR muskeg* OR quag* OR slough* OR "flood* meadow*" OR "wet* meadow*" OR "flood* forest*" OR "wet* forest*" OR "flood* grassland*" OR "wet* grassland*" OR "flood* heath*" OR "wet* heath*" OR "overflow zone*" OR "over flow zone*" OR "overflow area*" OR "over flow area*")) OR **TI** ((restor* OR construct* OR creat* OR manmade OR "man* made" OR rehabilit* OR inundat* OR rewet* OR "re-wet*" OR "clear cut*" OR dam OR dams OR damming OR "re-meander*" OR remeander* OR "grip block*" OR gripblock* OR "re-establish*" OR "re-vegetation" OR revegetation OR "re-vegetating" OR revegetating OR "vegetation removal" OR "remov* vegetation" OR "removal of vegetation" OR excavation OR excavating OR backfill* OR drain* OR ditch* OR "dry* out" OR "dried out") **N10** (wetland* OR "wet* land*" OR bog OR bogs OR bogland* OR carr OR carrs OR fen OR fens OR fenland* OR "flood plain*" OR floodplain* OR "river plain*" OR riverplain* OR marsh* OR mire* OR "peat land*" OR peatland* OR peatbog* OR pond* OR moor* OR highmoor* OR lowmoor* OR riparian* OR riverine* OR "river bank*" OR swamp* OR shore* OR aapa* OR histosol* OR morass* OR muskeg* OR quag* OR slough* OR "flood* meadow*" OR "wet* meadow*" OR "flood* forest*" OR "wet* forest*" OR "flood* grassland*" OR "wet* grassland*" OR "flood* heath*" OR "wet* heath*" OR "overflow zone*" OR "over flow zone*" OR "overflow area*" OR "over flow area*")) OR **AB** ((restor* OR construct* OR creat* OR manmade OR "man* made" OR rehabilit* OR inundat* OR rewet* OR "re-wet*" OR "clear cut*" OR dam OR dams OR damming OR "re-meander*" OR remeander* OR "grip block*" OR gripblock* OR "re-establish*" OR "re-vegetation" OR revegetation OR "re-vegetating" OR revegetating OR "vegetation removal" OR "remov* vegetation" OR "removal of vegetation" OR excavation OR excavating OR backfill* OR drain* OR ditch* OR "dry* out" OR "dried out") **N10** (wetland* OR "wet* land*" OR bog OR bogs OR bogland* OR carr OR carrs OR fen OR fens OR fenland* OR "flood plain*" OR floodplain* OR "river plain*" OR riverplain* OR marsh* OR mire* OR "peat land*" OR peatland* OR peatbog* OR pond* OR moor* OR highmoor* OR lowmoor* OR riparian* OR riverine* OR "river bank*" OR swamp* OR shore* OR aapa* OR histosol* OR morass* OR muskeg* OR quag* OR slough* OR "flood* meadow*" OR "wet* meadow*" OR "flood* forest*" OR "wet* forest*" OR "flood* grassland*" OR "wet* grassland*" OR "flood* heath*" OR "wet* heath*" OR "overflow zone*" OR "over flow zone*" OR "overflow area*" OR "over flow area*")) OR **KW** ((restor* OR construct* OR creat* OR manmade OR "man* made" OR rehabilit* OR inundat* OR rewet* OR "re-wet*" OR "clear cut*" OR dam OR dams OR damming OR "re-meander*" OR remeander* OR "grip block*" OR gripblock* OR "re-establish*" OR "re-vegetation" OR revegetation OR "re-vegetating" OR revegetating OR "vegetation removal" OR "remov* vegetation" OR "removal of vegetation" OR excavation OR excavating OR backfill* OR drain* OR ditch* OR "dry* out" OR "dried out") **N10** (wetland* OR "wet* land*" OR bog OR bogs OR bogland* OR carr OR carrs OR fen OR fens OR fenland* OR "flood plain*" OR floodplain* OR "river plain*" OR riverplain* OR marsh* OR mire* OR "peat land*" OR peatland* OR peatbog* OR pond* OR moor* OR highmoor* OR lowmoor* OR riparian* OR riverine* OR "river bank*" OR swamp* OR shore* OR aapa* OR histosol* OR morass* OR muskeg* OR quag* OR slough* OR "flood* meadow*" OR "wet* meadow*" OR "flood* forest*" OR "wet* forest*" OR "flood* grassland*" OR "wet* grassland*" OR "flood* heath*" OR "wet* heath*" OR "overflow zone*" OR "over flow zone*" OR "overflow area*" OR "over flow area*"))) | **21 699** |
|  | **Outcome: Change in groundwater level, storage or amount** |  |
| **2** | (**SU** (groundwater* OR "ground water*" OR "water table*" OR watertable* OR "water budget*" OR waterbudget* OR "water storag*" OR waterstorag* OR "hydraulic head" OR "pump* test*" OR "subsurface hydrolog*" OR "sub surface hydrolog*" OR piezometer* OR "observation well*" OR "monitoring well*" OR "water well*" OR "dip well*" OR dipwell* OR "bore hole*" OR borehole*) OR **TI** (groundwater* OR "ground water*" OR "water table*" OR watertable* OR "water budget*" OR waterbudget* OR "water storag*" OR waterstorag* OR "hydraulic head" OR "pump* test*" OR "subsurface hydrolog*" OR "sub surface hydrolog*" OR piezometer* OR "observation well*" OR "monitoring well*" OR "water well*" OR "dip well*" OR dipwell* OR "bore hole*" OR borehole*) OR **AB** (groundwater* OR "ground water*" OR "water table*" OR watertable* OR "water budget*" OR waterbudget* OR "water storag*" OR waterstorag* OR "hydraulic head" OR "pump* test*" OR "subsurface hydrolog*" OR "sub surface hydrolog*" OR piezometer* OR "observation well*" OR "monitoring well*" OR "water well*" OR "dip well*" OR dipwell* OR "bore hole*" OR borehole*) OR **KW** (groundwater* OR "ground water*" OR "water table*" OR watertable* OR "water budget*" OR waterbudget* OR "water storag*" OR waterstorag* OR "hydraulic head" OR "pump* test*" OR "subsurface hydrolog*" OR "sub surface hydrolog*" OR piezometer* OR "observation well*" OR "monitoring well*" OR "water well*" OR "dip well*" OR dipwell* OR "bore hole*" OR borehole*)) | **88 070** |
|  | **Combination of search strings** |  |
| **3** | 1 AND 2 | **1 704** |
|  | **Limit to language: English, Danish, French, German, Norwegian, Polish, Swedish** |  |
| **4** | AND (LA(english OR danish OR french OR german OR norwegian OR polish OR swedish)) | **1 681** |

* = Represents any group of characters, including no character

" " = Searches for an exact phrase
SU = Subject Terms
TI = Title
AB = Abstract
KW = Author-Supplied Keywords
LA = Language

N10 = A proximity operator to find terms within ten words from each other

**Database: CAB Abstracts (1973-)**

Database provider: Ovid

Date of search: December 10, 2021

| **No** | **Search string** | **Number of hits** |
| --- | --- | --- |
|  | **Intervention: Restoration, construction or drainage of wetlands** |  |
| **1** | ((restor* OR construct* OR creat* OR manmade OR "man* made" OR rehabilit* OR inundat* OR rewet* OR "re-wet*" OR "clear cut*" OR dam OR dams OR damming OR "re-meander*" OR remeander* OR "grip block*" OR gripblock* OR "re-establish*" OR "re-vegetation" OR revegetation OR "re-vegetating" OR revegetating OR "vegetation removal" OR "remov* vegetation" OR "removal of vegetation" OR excavation OR excavating OR backfill* OR drain* OR ditch* OR "dry* out" OR "dried out") **ADJ10** (wetland* OR "wet* land*" OR bog OR bogs OR bogland* OR carr OR carrs OR fen OR fens OR fenland* OR "flood plain*" OR floodplain* OR "river plain*" OR riverplain* OR marsh* OR mire* OR "peat land*" OR peatland* OR peatbog* OR pond* OR moor* OR highmoor* OR lowmoor* OR riparian* OR riverine* OR "river bank*" OR swamp* OR shore* OR aapa* OR histosol* OR morass* OR muskeg* OR quag* OR slough* OR "flood* meadow*" OR "wet* meadow*" OR "flood* forest*" OR "wet* forest*" OR "flood* grassland*" OR "wet* grassland*" OR "flood* heath*" OR "wet* heath*" OR "overflow zone*" OR "over flow zone*" OR "overflow area*" OR "over flow area*")).ti,ab,hw. | **30 843** |
|  | **Outcome: Change in groundwater level, storage or amount** |  |
| **2** | (groundwater* OR "ground water*" OR "water table*" OR watertable* OR "water budget*" OR waterbudget* OR "water storag*" OR waterstorag* OR "hydraulic head" OR "pump* test*" OR "subsurface hydrolog*" OR "sub surface hydrolog*" OR piezometer* OR "observation well*" OR "monitoring well*" OR "water well*" OR "dip well*" OR dipwell* OR "bore hole*" OR borehole*).ti,ab,hw. | **123 660** |
|  | **Combination of search strings** |  |
| **3** | 1 AND 2 | **3 657** |
|  | **Limit to language: English, Danish, French, German, Norwegian, Polish, Swedish** |  |
| **4** | Limit 3 to (english OR danish OR french OR german OR norwegian OR polish OR swedish) | **3 467** |

* = Represents any group of characters, including no character
" " = Searches for an exact phrase
.ti,ab,hw. = Title or Abstract or Heading words

ADJ10 = A proximity operator to find terms within ten words from each other

**Database: Directory of Open Access Journals (DOAJ)**

Database provider: Independent
Date of search: September 8, 2020

DOAJ is a community-curated list of open access journals and aims to be the starting point for information searches for quality, peer reviewed open access material.

| **Search string** (search in all fields) | **Number of hits** (duplicates included) |
| --- | --- |
| restor* AND wetland* AND groundwater  construct* AND wetland* AND groundwater  drain* AND wetland* AND groundwater  restor* AND wetland* AND "water table"  construct* AND wetland* AND "water table"  drain* AND wetland* AND "water table"  restor* AND peatland* AND groundwater  construct* AND peatland* AND groundwater  drain* AND peatland* AND groundwater  restor* AND peatland* AND "water table"  construct* AND peatland* AND "water table"  drain* AND peatland* AND "water table"  restor* AND bog AND groundwater  construct* AND bog AND groundwater  drain* AND bog AND groundwater  restor* AND bog AND "water table"  construct* AND bog AND "water table"  drain* AND bog AND "water table"  restor* AND fen AND groundwater  construct* AND fen AND groundwater  drain* AND fen AND groundwater  restor* AND fen AND "water table"  construct* AND fen AND "water table"  drain* AND fen AND "water table"  restor* AND mire AND groundwater  construct* AND mire AND groundwater  drain* AND mire AND groundwater  restor* AND mire AND "water table"  construct* AND mire AND "water table"  drain* AND mire AND "water table" | **31**  **34**  **45**  **22**  **10**  **25**  **10**  **2**  **38**  **41**  **10**  **72**  **3**  **0**  **12**  **16**  **3**  **26**  **7**  **1**  **15**  **8**  **2**  **14**  **4**  **2**  **10**  **24**  **4**  **23** |

* = Represents any group of characters, including no character
" " = Searches for an exact phrase

**Database: DiVA**

Database provider: Swedish universities and research institutions
Date of search: September 10, 2020

DiVA contains research publications and student theses from Swedish universities and research institutions.

| **Language** | **Search string** (search in all fields) | **Number of hits** (duplicates included) |
| --- | --- | --- |
| **English** | restor* AND wetland* AND groundwater  construct* AND wetland* AND groundwater  drain* AND wetland* AND groundwater  restor* AND wetland* AND "water table"  construct* AND wetland* AND "water table"  drain* AND wetland* AND "water table"  restor* AND peatland* AND groundwater  construct* AND peatland* AND groundwater  drain* AND peatland* AND groundwater  restor* AND peatland* AND "water table"  construct* AND peatland* AND "water table"  drain* AND peatland* AND "water table"  restor* AND bog AND groundwater  construct* AND bog AND groundwater  drain* AND bog AND groundwater  restor* AND bog AND "water table"  construct* AND bog AND "water table"  drain* AND bog AND "water table"  restor* AND fen AND groundwater  construct* AND fen AND groundwater  drain* AND fen AND groundwater  restor* AND fen AND "water table"  construct* AND fen AND "water table"  drain* AND fen AND "water table"  restor* AND mire AND groundwater  construct* AND mire AND groundwater  drain* AND mire AND groundwater  restor* AND mire AND "water table"  construct* AND mire AND "water table"  drain* AND mire AND "water table" | **7**  **24**  **18**  **2**  **3**  **2**  **3**  **0**  **11**  **3**  **0**  **2**  **1**  **0**  **1**  **0**  **0**  **1**  **2**  **0**  **1**  **0**  **0**  **1**  **0**  **0**  **7**  **2**  **0**  **0** |
| **Swedish** | våtmark* AND grundvatten*  torvmark* AND grundvatten*  myr* AND grundvatten*  damm* AND grundvatten*  moss* AND grundvatten*  kärr* AND grundvatten*  sump* AND grundvatten* lövsump* AND grundvatten*  barrsump* AND grundvatten*  strand* AND grundvatten*  stränder* AND grundvatten*  svämplan* AND grundvatten*  träsk* AND grundvatten* | **77**  **4**  **8**  **22**  **9**  **3**  **1**  **1**  **0**  **17**  **0**  **2**  **0** |

* = Represents any group of characters, including no character
" " = Searches for an exact phrase

**Database: ProQuest Natural Science Collection**

Database provider: ProQuest

Date of search: December 10, 2021

Including: AGRICOLA; Agricultural Science database; Aquatic Sciences and Fisheries Abstracts; Biological Science database; Biological Science index; Earth, atmosphere & Aquatic Science database; Environmental Science database; Environmental Science index; Meteorological & Geoastrophysical Abstracts

| **No** | **Search string** | **Number of hits** |
| --- | --- | --- |
|  | **Intervention: Restoration, construction or drainage of wetlands** |  |
| **1** | ti,ab,su((restor* OR construct* OR creat* OR manmade OR "man* made" OR rehabilit* OR inundat* OR rewet* OR "re-wet*" OR "clear cut*" OR dam OR dams OR damming OR "re-meander*" OR remeander* OR "grip block*" OR gripblock* OR "re-establish*" OR "re-vegetation" OR revegetation OR "re-vegetating" OR revegetating OR "vegetation removal" OR "remov* vegetation" OR "removal of vegetation" OR excavation OR excavating OR backfill* OR drain* OR ditch* OR "dry* out" OR "dried out") **NEAR/10** (wetland OR "wet* land" OR bog OR bogland OR carr OR fen OR fenland OR "flood plain" OR floodplain OR "river plain" OR riverplain OR marsh OR mire OR "peat land" OR peatland OR peatbog OR pond OR moor OR highmoor OR lowmoor OR riparian OR riverine OR "river bank" OR swamp OR shore OR aapa OR histosol OR morass OR muskeg OR quag OR slough OR meadow OR forest OR grassland OR heath OR "overflow zone" OR "over flow zone" OR "overflow area" OR "over flow area")) | **138 415** (duplicates included) |
|  | **Outcome: Change in groundwater level, storage or amount** |  |
| **2** | ti,ab,su(groundwater* OR "ground water*" OR "water table" OR watertable OR "water budget" OR waterbudget OR "water storag*" OR waterstorag* OR "hydraulic head" OR "pump* test" OR "subsurface hydrolog*" OR "sub surface hydrolog*" OR piezometer OR "observation well" OR "monitoring well" OR "water well" OR "dip well" OR dipwell OR "bore hole" OR borehole) | **410 934** (duplicates included) |
|  | **Combination of search strings** |  |
| **3** | 1 AND 2 | **10 710** (duplicates included) |
|  | **Limit to language: English, Danish, French, German, Norwegian, Polish, Swedish** |  |
| **4** | AND la.exact("ENG" OR "DAN" OR "FRE" OR "GER" OR "NOR" OR "POL" OR "SWE") | **7 614** (duplicates removed) |

* = Represents any group of characters, including no character (Formas ProQuest settings is configured to automatically search for the plural forms of the search terms)
" " = Searches for an exact phrase
ti,ab,su = Title or Abstract or All subjects & indexing
la.exact = Language

NEAR/10 = A proximity operator to find terms within ten words from each other

**Database: SwePub**
Database provider: National Library of Sweden
Date of search: September 10, 2020

SwePub contains references to articles, conference papers and dissertations published at Swedish universities and authorities.

| **Language** | **Search string** | **Number of hits** |
| --- | --- | --- |
| **English** | (restor* OR construct* OR drain*) **AND** (wetland* OR peatland* OR bog OR fen OR mire) **AND** (groundwater OR "ground water" OR "water table") | **87** |
| **Swedish** | (våtmark* OR torvmark* OR myr* OR damm* OR moss* OR kärr* OR sump* OR lövsump* OR barrsump* OR strand* OR stränder* OR svämplan* OR träsk*) **AND** (grundvatten*) | **75** |

* = Represents any group of characters, including no character
" " = Searches for an exact phrase

**Search engine**

**Search engine: Google Scholar**

Date of search: September 10, 2020
The first 200 results for every search were exported from Google Scholar using Publish or Perish version 6 software: Harzing, A.W. (2007) Publish or Perish, available from <https://harzing.com/resources/publish-or-perish>

| **Language** | **Search string** | **Number of hits** |
| --- | --- | --- |
| **English** | **Wetland and groundwater**  Any of the words: "restored wetland” "constructed wetland" "drained wetland” All of the words: groundwater  None of the words: wastewater  **Peatland and groundwater**  Any of the words: "restored peatland” "constructed peatland" "drained peatland” All of the words: groundwater  None of the words: wastewater  **Wetland and water table**  Any of the words: "restored wetland” "constructed wetland" "drained wetland” All of the words: "water table"  None of the words: wastewater  **Peatland and water table**  Any of the words: "restored peatland” "constructed peatland" "drained peatland” All of the words: "water table"  None of the words: wastewater | **200** (generated more hits, but we only imported the first 200)  **200** (generated more hits, but we only imported the first 200)  **200** (generated more hits, but we only imported the first 200)  **200** (generated more hits, but we only imported the first 200) |
| **Swedish** | **Våtmark och grundvatten (Wetland and groundwater)**  Any of the words: restaurering restaurera anlagd anlägga dränering dränera  All of the words: våtmark grundvatten  None of the words: avloppsvatten  **Torvmark och grundvatten (Peatland and groundwater)**  Any of the words: restaurering restaurera anlagd anlägga dränering dränera  All of the words: torvmark grundvatten  None of the words: avloppsvatten  **Myr och grundvatten (Bog and groundwater)**  Any of the words: restaurering restaurera anlagd anlägga dränering dränera  All of the words: myr grundvatten  None of the words: avloppsvatten | **200** (generated more hits, but we only imported the first 200)  **200** (generated more hits, but we only imported the first 200)  **200** (generated more hits, but we only imported the first 200) |

" " = Searches for an exact phrase
No truncation (*) is used because Google Scholar automatically searches plural forms of the search terms

**Websites of relevant organisations**

The search languages used on these websites are English or Swedish and sometimes both, depending on the language of the website.

| **Website** | **Date** | **Search string** | **Number of potentially relevant documents** |
| --- | --- | --- | --- |
| EEA (European Environment Agency) <https://www.eea.europa.eu> | March 18, 2021 | **Used the search box on the publications page**  wetland*  peat*  bog*  fen*  mire* | **3**  **0**  **0**  **0**  **0** |
| European Commission Joint Research Centre  https://ec.europa.eu/info/departments/joint-research-centre_en | March 18, 2021 | **Used the search box on the publications page**  wetland  peat  bog  fen  mire | **0**  **0**  **0**  **0**  **0** |
| Miljøstyrelsen (Danish Environmental Protection Agency) <https://mst.dk> | March 18, 2021 | **Used the regular search box for the entire website** wetland*  peat*  bog*  fen*  mire* | **0**  **0**  **0**  **0**  **0** |
| Luke (Natural Resources Institute Finland) <https://www.luke.fi> | March 22, 2021 | **Used Advanced search in their Jukuri publication service. https://jukuri.luke.fi/discover?query=&scope=**  wetland* AND groundwater*  wetland* AND "ground water*"  wetland* AND watertable*  wetland* AND "water table*" peatland* AND groundwater*  peatland* AND "ground water*"  peatland* AND watertable*  peatland* AND "water table*"  "peat land*" AND groundwater*  "peat land*" AND "ground water*"  "peat land*" AND watertable*  "peat land*" AND "water table*" bog AND groundwater*  bog AND "ground water*"  bog AND watertable*  bog AND "water table*"  fen AND groundwater*  fen AND "ground water*"  fen AND watertable*  fen AND "water table*"  mire* AND groundwater*  mire* AND "ground water*"  mire* AND watertable*  mire* AND "water table*"  våtmark* AND grundvatten* torv* AND grundvatten*  myr* AND grundvatten* | **23**  **9**  **5**  **14**  **4**  **5**  **3**  **10**  **0**  **0**  **0**  **0**  **1**  **0**  **0**  **3**  **0**  **0**  **1**  **4**  **0**  **0**  **0**  **1**  **0**  **1**  **0** |
| Metsähallitus (Steward of state-owned land and water areas in Finland)  https://www.metsa.fi/  https://julkaisut.metsa.fi/ | March 30, 2021 | **Used the free text search box on their publications site**  **https://julkaisut.metsa.fi**  wetland  wetlands  peatland  peatlands  peat land  peat lands  bog  bogs  fen  fens  mire  mires  våtmark  våtmarker torvmark  torvmarker  myr  myrmark  myrmarker | **2**  **0**  **7**  **3**  **3**  **0**  **0**  **0**  **0**  **0**  **2**  **1**  **0**  **0**  **0**  **0**  **1**  **0**  **0** |
| SYKE (Finnish Environment Institute)  https://www.syke.fi | March 31, 2021 | **Used simple search in their publications archive**  **https://helda.helsinki.fi/handle/10138/29865?locale-attribute=en**  wetland* AND groundwater*  wetland* AND "ground water*"  wetland* AND watertable*  wetland* AND "water table*" peatland* AND groundwater*  peatland* AND "ground water*"  peatland* AND watertable*  peatland* AND "water table*"  "peat land*" AND groundwater*  "peat land*" AND "ground water*"  "peat land*" AND watertable*  "peat land*" AND "water table*" bog AND groundwater*  bog AND "ground water*"  bog AND watertable*  bog AND "water table*"  fen AND groundwater*  fen AND "ground water*"  fen AND watertable*  fen AND "water table*"  mire* AND groundwater*  mire* AND "ground water*"  mire* AND watertable*  mire* AND "water table*"  våtmark* AND grundvatten* torv* AND grundvatten*  myr* AND grundvatten* | **18**  **1**  **8**  **20**  **4**  **3**  **1**  **3**  **3**  **2**  **0**  **0**  **2**  **1**  **0**  **0**  **1**  **0**  **0**  **0**  **0**  **0**  **0**  **0**  **0**  **0**  **0** |
| EPA Ireland (Environmental Protection Agency, Ireland)  http://epa.ie | April 8, 2021 | **Used the search box on the publications page**  wetland AND groundwater  wetland AND "ground water"  wetland AND watertable  wetland AND "water table" peatland AND groundwater  peatland AND "ground water"  peatland AND watertable  peatland AND "water table" bog AND groundwater  bog AND "ground water"  bog AND watertable  bog AND "water table"  fen AND groundwater  fen AND "ground water"  fen AND watertable  fen AND "water table"  mire AND groundwater  mire AND "ground water"  mire AND watertable  mire AND "water table" | **3**  **0**  **0**  **0**  **5**  **0**  **0**  **1**  **1**  **0**  **0**  **0**  **0**  **0**  **0**  **0**  **0**  **0**  **0**  **0** |
| Deltares  https://www.deltares.nl/en/ | April 8, 2021 | **Used the search box on the publications page**  wetland*  peatland*  bog*  fen*  mire* | **14**  **3**  **0**  **1**  **0** |
| PBL Netherlands Environmental Assessment Agency  https://www.pbl.nl/ | April 9, 2021 | **Used the search box on the publications page**  wetland  peatland  bog  fen  mire | **4**  **0**  **0**  **0**  **0** |
| NIVA (Norwegian Institute for Water Research)  https://www.niva.no | April 9, 2021 | **Used the regular search box for the entire website**  wetland*  peat*  bog*  fen*  mire* | **0**  **0**  **0**  **0**  **0** |
| IVL (Swedish Environmental Research Institute)  https://www.ivl.se | April 9, 2021 | **Used the search box on the publications page**  wetland*  peat*  bog*  fen*  mire*  våtmark* torv*  myr* | **0**  **2**  **0**  **0**  **0**  **3**  **0**  **0** |
| Jordbruksverket (Swedish Board of Agriculture)  https://jordbruksverket.se/ | April 9, 2021 | **Used the search box on the publications page https://webbutiken.jordbruksverket.se/**  våtmark torv  myr | **10**  **1**  **0** |
| Länsstyrelsen Blekinge (County Administrative Board of Blekinge, Sweden) https://www.lansstyrelsen.se/blekinge | April 12, 2021 | **Used the regular search box for the entire website**  våtmark*  torv* myr* | **4**  **0**  **0** |
| Länsstyrelsen Dalarna (County Administrative Board of Dalarna, Sweden) https://www.lansstyrelsen.se/dalarna | April 12, 2021 | **Used the regular search box for the entire website**  våtmark* torv* myr* | **0**  **0**  **0** |
| Länsstyrelsen Gotland (County Administrative Board of Gotland, Sweden) https://www.lansstyrelsen.se/gotland | April 12, 2021 | **Used the regular search box for the entire website**  våtmark* torv* myr* | **4**  **0**  **0** |
| Länsstyrelsen Gävleborg (County Administrative Board of Gävleborg, Sweden) https://www.lansstyrelsen.se/gavleborg | April 12, 2021 | **Used the regular search box for the entire website**  våtmark* torv* myr* | **0**  **0**  **0** |
| Länsstyrelsen Halland (County Administrative Board of Halland, Sweden) https://www.lansstyrelsen.se/halland | April 12, 2021 | **Used the regular search box for the entire website**  våtmark* torv* myr* | **1**  **0**  **0** |
| Länsstyrelsen Jämtland (County Administrative Board of Jämtland, Sweden) https://www.lansstyrelsen.se/jamtland | April 12, 2021 | **Used the regular search box for the entire website**  våtmark* torv* myr* | **2**  **0**  **0** |
| Länsstyrelsen Jönköping (County Administrative Board of Jönköping, Sweden) https://www.lansstyrelsen.se/jonkoping | April 12, 2021 | **Used the regular search box for the entire website**  våtmark* torv* myr* | **7**  **0**  **0** |
| Länsstyrelsen Kalmar (County Administrative Board of Kalmar, Sweden) https://www.lansstyrelsen.se/kalmar | April 12, 2021 | **Used the regular search box for the entire website**  våtmark* torv* myr* | **2**  **0**  **0** |
| Länsstyrelsen Kronoberg (County Administrative Board of Kronoberg, Sweden) https://www.lansstyrelsen.se/kronoberg | April 12, 2021 | **Used the regular search box for the entire website**  våtmark* torv* myr* | **1**  **0**  **1** |
| Länsstyrelsen Norrbotten (County Administrative Board of Norrbotten, Sweden) https://www.lansstyrelsen.se/norrbotten | April 12, 2021 | **Used the regular search box for the entire website**  våtmark* torv* myr* | **1**  **0**  **0** |
| Länsstyrelsen Skåne (County Administrative Board of Skåne, Sweden) https://www.lansstyrelsen.se/skane | April 13, 2021 | **Used the regular search box for the entire website**  våtmark* torv* myr* | **2**  **0**  **0** |
| Länsstyrelsen Stockholm (County Administrative Board of Stockholm, Sweden) https://www.lansstyrelsen.se/stockholm | April 13, 2021 | **Used the regular search box for the entire website**  våtmark* torv* myr* | **2**  **0**  **0** |
| Länsstyrelsen Södermanland (County Administrative Board of Södermanland, Sweden) <https://www.lansstyrelsen.se/sodermanland> | April 13, 2021 | **Used the regular search box for the entire website**  våtmark* torv* myr* | **1**  **0**  **0** |
| Länsstyrelsen Uppsala (County Administrative Board of Uppsala, Sweden) https://www.lansstyrelsen.se/uppsala | April 13, 2021 | **Used the regular search box for the entire website**  våtmark* torv* myr* | **1**  **0**  **0** |
| Länsstyrelsen Värmland (County Administrative Board of Värmland, Sweden) https://www.lansstyrelsen.se/varmland | April 13, 2021 | **Used the regular search box for the entire website**  våtmark* torv* myr* | **1**  **0**  **0** |
| Länsstyrelsen Västerbotten (County Administrative Board of Västerbotten, Sweden) https://www.lansstyrelsen.se/vasterbotten | April 13, 2021 | **Used the regular search box for the entire website**  våtmark* torv* myr* | **2**  **0**  **0** |
| Länsstyrelsen Västernorrland (County Administrative Board of Västernorrland, Sweden) https://www.lansstyrelsen.se/vasternorrland | April 13, 2021 | **Used the regular search box for the entire website**  våtmark* torv* myr* | **2**  **0**  **0** |
| Länsstyrelsen Västmanland (County Administrative Board of Västmanland, Sweden) https://www.lansstyrelsen.se/vastmanland | April 13, 2021 | **Used the regular search box for the entire website**  våtmark* torv* myr* | **0**  **0**  **0** |
| Länsstyrelsen Västra Götaland (County Administrative Board of Västra Götaland, Sweden) https://www.lansstyrelsen.se/vastra-gotaland | April 13, 2021 | **Used the regular search box for the entire website**  våtmark* torv* myr* | **4**  **0**  **0** |
| Länsstyrelsen Örebro (County Administrative Board of Örebro, Sweden) https://www.lansstyrelsen.se/orebro | April 13, 2021 | **Used the regular search box for the entire website**  våtmark* torv* myr* | **3**  **0**  **0** |
| Länsstyrelsen Östergötland (County Administrative Board of Östergötland, Sweden) https://www.lansstyrelsen.se/ostergotland | April 13, 2021 | **Used the regular search box for the entire website**  våtmark* torv* myr* | **3**  **0**  **1** |
| Naturvårdsverket (Swedish Environmental Protection Agency) <http://www.naturvardsverket.se> | April 14, 2021 | **Used the regular search box for the entire website Limit: Publications**  våtmark* torv* myr* | **14**  **0**  **0** |
| SGU (Geological Survey of Sweden)  https://www.sgu.se | April 14, 2021 | **Used the regular search box for the entire website Limit: Publications**  våtmark* torv* myr* | **3**  **6**  **1** |
| Skogsstyrelsen (Swedish Forest Agency)  https://www.skogsstyrelsen.se | April 14, 2021 | **Used the regular search box for the entire website Limit: Files**  våtmark* torv* myr* | **8**  **2**  **1** |
| SMED (Swedish Environmental Emissions Data)  https://www.smed.se | April 15, 2021 | **Used the regular search box for the entire website**  **Automatic truncation is used (it's a default functionality)**  våtmark torv myr | **2**  **0**  **6** |
| SMHI (Swedish Meteorological and Hydrological Institute)  https://www.smhi.se/ | April 15, 2021 | **Used the regular search box for the entire website Limit: Publications**  wetland*  peatland*  bog*  fen*  mire*  våtmark* torv* myr* | **10**  **0**  **0**  **0**  **2**  **4**  **0**  **0** |
| SKB (Swedish Nuclear Waste Management Company)  https://www.skb.se/ | April 15, 2021 | **Used the search box on the publications page**  **Search field: Title**  våtmark  våtmarker  torvmark  torvmarker myr  myrmark  myrmarker | **2**  **2**  **0**  **0**  **0**  **0**  **0** |
| Vattenmyndigheterna (Swedish Water Authorities)  https://www.vattenmyndigheterna.se/ | April 16, 2021 | **Used the regular search box for the entire website Limit: Publications**  våtmark* torv* myr* | **1**  **0**  **0** |
| DEFRA (Department for Environment, Food & Rural Affairs)  https://www.gov.uk/government/organisations/department-for-environment-food-rural-affairs | April 16, 2021 | **Used the regular search box for the entire website**  **Limit to Content type: "Research and statistics"**  wetland*  peatland*  bog*  fen*  mire* | **0**  **0**  **0**  **0**  **0** |
| SEPA (Scottish Environmental Protection Agency)  https://www.sepa.org.uk | April 16, 2021 | **Used the search box on the publications page**  **https://www.sepa.org.uk/library/** wetland*  peat*  bog*  fen*  mire* | **2**  **1**  **1**  **0**  **0** |
| UK Centre for Ecology & Hydrology  https://www.ceh.ac.uk/ | April 16, 2021 | **Used the regular search box for the entire website**  wetland*  peat*  bog*  fen*  mire* | **0**  **0**  **0**  **0**  **0** |
| UK Environment Agency  https://www.gov.uk/government/organisations/environment-agency | April 16, 2021 | **Used the regular search box for the entire website**  **Limit to Content type: "Research and statistics"**  wetland*  peatland*  bog*  fen*  mire* | **9**  **0**  **0**  **1**  **0** |
| Association of State Wetland Managers  https://www.aswm.org/ | April 16, 2021 | **Browsed through their page on wetland restorations**  **https://www.aswm.org/aswm/publications/aswm-publications/7673-restoration** | **7** |
| Environment and Climate Change Canada  https://www.canada.ca/en/environment-climate-change.html | April 16, 2021 | **Used Advanced Search  http://www.publications.gc.ca/site/eng/search/advancedSearch.html**  **Search Field: Title**  **Format: PDF**  wetland*  peat*  bog*  fen*  mire* | **0**  **0**  **0**  **0**  **0** |
| EPA U.S. (Environmental Protection Agency, United States) <https://www.epa.gov/> | April 19-20, 2021 | **Used Fields Search on National Service Center for Environmental Publications (NSCEP) http://nepis.epa.gov/Fields.html Search Field: Title Results Precision: Exact match**  **Limits: Only PDF-publications**  wetland  wetlands  peatland  peatlands  bog  bogs  fen  fens  mire  mires | **10**  **23**  **0**  **0**  **0**  **1**  **0**  **0**  **0**  **0** |
| U.S. Geological Survey  https://www.usgs.gov/ | April 19-20, 2021 | **Used the regular search box for the entire website**  **Limit to: Publications**  **Type of document: Report**  wetland AND groundwater  wetland AND "ground water"  wetland AND watertable  wetland AND "water table"  wetlands AND groundwater  wetlands AND "ground water"  wetlands AND watertable  wetlands AND "water table" peatland AND groundwater  peatland AND "ground water"  peatland AND watertable  peatland AND "water table"  "peat land" AND groundwater  "peat land" AND "ground water"  "peat land" AND watertable  "peat land" AND "water table"  peatlands AND groundwater  peatlands AND "ground water"  peatlands AND watertable  peatlands AND "water table"  "peat lands" AND groundwater  "peat lands" AND "ground water"  "peat lands" AND watertable  "peat lands" AND "water table" bog AND groundwater  bog AND "ground water"  bog AND watertable  bog AND "water table"  bogs AND groundwater  bogs AND "ground water"  bogs AND watertable  bogs AND "water table"  fen AND groundwater  fen AND "ground water"  fen AND watertable  fen AND "water table"  fens AND groundwater  fens AND "ground water"  fens AND watertable  fens AND "water table"  mire AND groundwater  mire AND "ground water"  mire AND watertable  mire AND "water table"  mires AND groundwater  mires AND "ground water"  mires AND watertable  mires AND "water table" | **10**  **21**  **1**  **6**  **7**  **13**  **0**  **2**  **0**  **0**  **0**  **0**  **0**  **0**  **0**  **2**  **1**  **0**  **0**  **0**  **0**  **0**  **0**  **0**  **2**  **3**  **0**  **0**  **0**  **2**  **0**  **0**  **0**  **0**  **0**  **0**  **0**  **0**  **0**  **0**  **0**  **0**  **0**  **0**  **0**  **0**  **0**  **0** |
| GWEN (Global Wetland Ecohydrology Network)  https://www.gwennetwork.se | April 20, 2021 | **Browsed through their publications page**  **https://www.gwennetwork.se/?page_id=37** | **4** |
| Ramsar  https://ramsar.org | April 20-21, 2021 | **Used the search box on the documents page**  **https://ramsar.org/search?f%5B0%5D=type%3Adocument**  restor* AND wetland* AND groundwater  construct* AND wetland* AND groundwater  drain* AND wetland* AND groundwater  restor* AND wetland* AND "water table"  construct* AND wetland* AND "water table"  drain* AND wetland* AND "water table"  restor* AND peatland* AND groundwater  construct* AND peatland*AND groundwater  drain* AND peatland*AND groundwater  restor* AND peatland*AND "water table"  construct* AND peatland* AND "water table"  drain* AND peatland* AND "water table"  restor* AND bog* AND groundwater  construct* AND bog*AND groundwater  drain* AND bog*AND groundwater  restor* AND bog*AND "water table"  construct* AND bog* AND "water table"  drain* AND bog* AND "water table"  restor* AND fen* AND groundwater  construct* AND fen*AND groundwater  drain* AND fen*AND groundwater  restor* AND fen*AND "water table"  construct* AND fen* AND "water table"  drain* AND fen* AND "water table"  restor* AND mire* AND groundwater  construct* AND mire*AND groundwater  drain* AND mire*AND groundwater  restor* AND mire*AND "water table"  construct* AND mire* AND "water table"  drain* AND mire* AND "water table" | **12**  **2**  **2**  **1**  **0**  **0**  **0**  **0**  **0**  **0**  **0**  **1**  **0**  **0**  **0**  **0**  **1**  **0**  **0**  **0**  **1**  **0**  **0**  **0**  **0**  **0**  **0**  **0**  **0**  **0** |
| Society of Wetland Scientists  https://www.sws.org | April 20, 2021 | **Not able to search or browse publications on their website** |  |
| Wetlands International  https://www.wetlands.org | April 20, 2021 | **Used the regular search box for the entire website**  **Post type: Publications**  groundwater  ground water  water table | **0**  **1**  **0** |
