# Supplementary material for: Effects on groundwater storage of restoring, constructing or draining wetlands in temperate and boreal climates: a systematic review
Source: Environ Evid. 2022 Dec 8;11:38. doi: 10.1186/s13750-022-00289-5 (PMC11378806; doi:10.1186/s13750-022-00289-5)
Supplement: Supplementary file 4 — Additional file 4. List of included articles. [file 13750_2022_289_MOESM4_ESM.docx]

Additional file 4. List of included articles

Addy, S., Wilkinson, M.E., 2021. Embankment lowering and natural self-recovery improves river-floodplain hydro-geomorphic connectivity of a gravel bed river. Sci. Total Environ. 770. <https://doi.org/10.1016/j.scitotenv.2020.144626>

Ahmad, S., Liu, H., Alam, S., Günther, A., Jurasinski, G., Lennartz, B., 2021. Meteorological Controls on Water Table Dynamics in Fen Peatlands Depend on Management Regimes. Front. Earth Sci. 9. <https://doi.org/10.3389/feart.2021.630469>

Ahmad, S., Liu, H., Günther, A., Couwenberg, J., Lennartz, B., 2020. Long-term rewetting of degraded peatlands restores hydrological buffer function. Sci. Total Environ. 749. <https://doi.org/10.1016/j.scitotenv.2020.141571>

Ahti, E., Paivanen, J., 1997. Response of stand growth and water table level to maintenance of ditch networks within forest drainage areas, in: Trettin, C.C., Jurgensen, M., Grigal, D., Gale, M., Jeglum, J. (Eds.), Northern Forested Wetlands: Ecology and Management. CRC Press, Boca Raton, Fl, USA, pp. 449–457.

Alderson, D.M., Evans, M.G., Shuttleworth, E.L., Pilkington, M., Spencer, T., Walker, J., Allott, T.E.H., 2019. Trajectories of ecosystem change in restored blanket peatlands. Sci. Total Environ. 665, 785–796.

Anderson, R., 2010. Restoring afforested peat bogs: results of current research. Research Note - Forestry Commission; 2010. (006):8 pp. 4 ref., Forestry Commission, Edinburgh, UK.

Anderson, R., Peace, A., 2017. Ten-year results of a comparison of methods for restoring afforested blanket bog. Mires Peat 19, 23. <https://doi.org/10.19189/MaP.2015.OMB.214>

Audet, J., Elsgaard, L., Kjaergaard, C., Larsen, S.E., Hoffmann, C.C., 2013. Greenhouse gas emissions from a Danish riparian wetland before and after restoration. Ecol. Eng. 57, 170–182.

Beetz, S., Liebersbach, H., Glatzel, S., Jurasinski, G., Buczko, U., Hoper, H., 2013. Effects of land use intensity on the full greenhouse gas balance in an Atlantic peat bog. Biogeosciences 10, 1067–1082.

Belleau, P., Plamondon, A.P., Lagace, R., Pepin, S., 1992. Hydrodynamics of a drained black spruce bog. Canadian Journal of Forest Research 22, 1063–1070.

Bergquist, B., Lundin, L., Andersson, A., 1984. Hydrologiska och limnologiska konsekvenser av skogs-och myrdikning. Siksjöbäcksområdet. Effects of peatland drainage on hydrology and limnology. The basin Siksjöbäcken. (Rapport LIU 1984 B:4 No. 9), Forskningsrapport. Limnologiska institutionen, Uppsala.

Berry, G.J., Jeglum, J.K., 1991. Hydrology of drained and undrained black spruce peatlands: groundwater table profiles and fluctuations (COFRDA Report 3307:v + 39 pp. 43 ref.), Forestry Canada, Ontario Region. Sault Ste. Marie, Ontario, Canada.

Berry, G.J., Jeglum, J.K., 1988. Water table profiles of drained forested and clearcut peatlands in northern Ontario, Canada. Suomen Akatemian Julkaisuja 5, 72–79.

Bess, J.A., Chimner, R.A., Kangas, L.C., 2014. Ditch restoration in a large Northern Michigan Fen: Vegetation response and basic porewater chemistry. Ecol. Restor. 32, 260–274.

Bieniada, A., Strack, M., 2021. Steady and ebullitive methane fluxes from active, restored and unrestored horticultural peatlands. Ecol. Eng. 169. <https://doi.org/10.1016/j.ecoleng.2021.106324>

Blankenburg, J., 2009. Renaturing and Rewetting of Bogs and Flood Plains in the Light of Diffuse Shifts in Material Loads. KW - Korrespondenz Wasserwirtschaft 2, 146–150.

Boczon, A., Wrobel, M., Syniaiev, V., 2009. The impact of beaver ponds on water resources in the catchment area in the Browsk Forest District - a case study. Lesne Prace Badawcze 70, 363–371.

Boelter, D., 1972. Water table drawdown around an open ditch in organic soils. Journal of Hydrology 15, 329–340. <https://doi.org/10.1016/0022-1694(72)90046-7>

Bonsel, A., Sonneck, A.G., 2011. Effects of a hydrological protection zone on the restoration of a raised bog: a case study from Northeast-Germany 1997-2008. Wetl. Ecol. Manag. 19, 183–194.

Bouwes, N., Weber, N., Jordan, C.E., Saunders, W.C., Tattam, I.A., Volk, C., Wheaton, J.M., Pollock, M.M., 2016. Ecosystem experiment reveals benefits of natural and simulated beaver dams to a threatened population of steelhead (Oncorhynchus mykiss). Scientific reports 6, 1–12. <https://doi.org/10.1038/srep28581>

Burdun, I., Kull, A., Maddison, M., Veber, G., Karasov, O., Sagris, V., Mander, U., 2021. Remotely Sensed Land Surface Temperature Can Be Used to Estimate Ecosystem Respiration in Intact and Disturbed Northern Peatlands. Journal of Geophysical Research-Biogeosciences 126. <https://doi.org/10.1029/2021JG006411>

Burke, W., 1975. Effect of drainage on the hydrology of blanket bog. Irish Journal of Agricultural Research 14, 145–162.

Buszka, P.M., Cohen, D.A., Lampe, D.C., Pavlovic, N.B., 2011. Relation of hydrologic processes to groundwater and surface-water levels and flow directions in a dune-beach complex at Indiana Dunes National Lakeshore and Beverly Shores, Indiana (Scientific Investigations Report No. 2011–5073). U.S. Geological Survey.

Byrne, K.A., Farrell, E.P., 2005. The effect of afforestation on soil carbon dioxide emissions in blanket peatland in Ireland. Forestry 78, 217–227.

Cao, R., Xi, X.Q., Yang, Y.H.S., Wei, X., Wu, X.W., Sun, S.C., 2017. The effect of water table decline on soil CO2 emission of Zoige peatland on eastern Tibetan Plateau: A four-year in situ experimental drainage. Appl. Soil Ecol. 120, 55–61.

Chimner, R.A., Cooper, D.J., Bidwell, M.D., Culpepper, A., Zillich, K., Nydick, K., 2019. A new method for restoring ditches in peatlands: ditch filling with fiber bales. Restor. Ecol. 27, 63–69.

Chimner, R.A., Pypker, T.G., Hribljan, J.A., Moore, P.A., Waddington, J.M., 2017. Multi-decadal Changes in Water Table Levels Alter Peatland Carbon Cycling. Ecosystems 20, 1042–1057.

Christen, A., Jassal, R.S., Black, T.A., Grant, N.J., Hawthorne, I., Johnson, M.S., Lee, S.C., Merkens, M., 2016. Summertime greenhouse gas fluxes from an urban bog undergoing restoration through rewetting. Mires Peat 17, 24.

Clilverd, H.M., Thompson, J.R., Heppell, C.M., Sayer, C.D., Axmacher, J.C., 2016. Coupled Hydrological/Hydraulic Modelling of River Restoration Impacts and Floodplain Hydrodynamics. River Res. Appl. 32, 1927–1948.

Cooper, D.J., Kaczynski, K.M., Sueltenfuss, J., Gaucherand, S., Hazen, C., 2017. Mountain wetland restoration: The role of hydrologic regime and plant introductions after 15 years in the Colorado Rocky Mountains, USA. Ecol. Eng. 101, 46–59.

Cooper, D.J., MacDonald, L.H., Wenger, S.K., Woods, S.W., 1998. Hydrologic restoration of a fen in Rocky Mountain National Park, Colorado, USA. Wetlands 18, 335–345.

Cooper, M.D.A., Evans, C.D., Zielinski, P., Levy, P.E., Gray, A., Peacock, M., Norris, D., Fenner, N., Freeman, C., 2014. Infilled Ditches are Hotspots of Landscape Methane Flux Following Peatland Re-wetting. Ecosystems 17, 1227–1241.

Cowdery, T.K., Christenson, C.A., Ziegeweid, J.R., 2019. The Hydrologic Benefits of Wetland and Prairie Restoration in Western Minnesota-Lessons Learned at the Glacial Ridge National Wildlife Refuge, 2002–15 (Scientific Investigations Report No. 2019–5041). U.S. Geological Survey, Reston.

Czerepko, J., Boczon, A., Wrobel, M., Gawrys, R., Sokolowski, K., 2018. Removal of birch as a means of protecting raised bog mossy vegetation Ledo-Sphagnetum magellanici. Wetl. Ecol. Manag. 26, 689–702.

Czerepko, J., Wróbel, M., Boczoń, A., SokoŁowski, K., 2009. The response of ash-alder swamp forest to increasing stream water level caused by damming by the European beaver (Castor fiber L.). J. Water Land Dev. 13, 249–262.

Danyluk, A., 2012. Tree removal as a tool of ecological restoration in Burns Bog, Delta, B.C (MSc thesis). Royal Roads University, Victoria, BC, Canada.

de Louw, P.G.B., Stuurman, R.J., Means, J.L., Hinchee, R.E., 2000. Regional and local hydrological influences on a brook valley wetland system, in: Wetlands & Remediation: An International Conference. Battelle Press, Delft, Netherlands, pp. 111–118.

Dixon, S.D., Qassim, S.M., Rowson, J.G., Worrall, F., Evans, M.G., Boothroyd, I.M., Bonn, A., 2014. Restoration effects on water table depths and CO2 fluxes from climatically marginal blanket bog. Biogeochemistry 118, 159–176.

Doležal, T., Vlček, L., Kocum, J., Jansky, B., 2017. Evaluation of the influence of mountain peat bogs restoration measures on the groundwater level: Case study rokytka peat bog, the šumava mts., Czech Republic. Acta Univ. Carol. Geogr. 52, 141–150.

Drewnik, M., Rajwa-Kuligiewicz, A., Stolarczyk, M., Kucharzyk, S., Zelazny, M., 2018. Intra-annual groundwater levels and water temperature patterns in raised bogs affected by human impact in mountain areas in Poland. Sci. Total Environ. 624, 991–1003.

Engman, A., Kero, J., Oleskog, A., Pierrau, H., Tholander, J., 2020. Karaktärisering av våtmarker med hydrologiska syften och analys av våtmarkers effekt på hydrologin : En studie av anlagda, restaurerade och planerade våtmarker i Sverige (MSc thesis). Uppsala University, Uppsala, Sweden.

Essery, C.J., Wilcock, D.N., 1990. Impact of Channelization on the Hydrology of the Upper River Main, County Antrim, Northern Ireland: A Long-Term Case Study. Regulated Rivers Research & Management RRRMEP Vol. 5, No. 1, p 17-34, January/February 1990. 10 fig, 2 tab, 25 ref.

Farrell, C.A., Doyle, G.J., 2003. Rehabilitation of industrial cutaway Atlantic blanket bog in County Mayo, North-West Ireland. Wetl. Ecol. Manag. 11, 21–35.

Feiner, K., Lowry, C.S., 2015. Simulating the effects of a beaver dam on regional groundwater flow through a wetland. J. Hydrol.-Reg. Stud. 4, 689–699.

Fox, A.D., 1986. Effects of ditch-blockage on adult Odonata at a coastal raised mire site in central west Wales, United Kingdom. Odonatologica 15, 327–334.

Frank, S., Tiemeyer, B., Gelbrecht, J., Freibauer, A., 2014. High soil solution carbon and nitrogen concentrations in a drained Atlantic bog are reduced to natural levels by 10 years of rewetting. Biogeosciences 11, 2309–2324.

Gaffney, P.P.J., Hancock, M.H., Taggart, M.A., Andersen, R., 2018. Measuring restoration progress using pore- and surface-water chemistry across a chronosequence of formerly afforested blanket bogs. J. Environ. Manage. 219, 239–251.

Gaffney, P.P.J., Hugron, S., Jutras, S., Marcoux, O., Raymond, S., Rochefort, L., 2020. Ecohydrological change following rewetting of a deep-drained northern raised bog. Ecohydrology 13, 15.

Gatis, N., Luscombe, D.J., Benaud, P., Ashe, J., Grand-Clement, E., Anderson, K., Hartley, I.P., Brazier, R.E., 2020. Drain blocking has limited short-term effects on greenhouse gas fluxes in a Molinia caerulea dominated shallow peatland. Ecol. Eng. 158, 106079. <https://doi.org/10.1016/j.ecoleng.2020.106079>

Glatzel, S., Kalbitz, K., Dalva, M., Moore, T., 2003. Dissolved organic matter properties and their relationship to carbon dioxide efflux from restored peat bogs. Geoderma 113, 397–411.

Glina, B., Bogacz, A., Mendyk, L., Bojko, O., Nowak, M., 2018. Effectiveness of restoration of a degraded shallow mountain fen after five years. Mires Peat 21, 15.

Gorn, S., Fischer, K., 2015. Measuring the efficiency of fen restoration on carabid beetles and vascular plants: a case study from north-eastern Germany. Restor. Ecol. 23, 413–420.

Graf, M.D., Rochefort, L., Poulin, M., 2008. Spontaneous revegetation of cutwaway peatlands of North America. Wetlands 28, 28–39.

Green, S.M., Baird, A.J., Holden, J., Reed, D., Birch, K., Jones, P., 2017. An experimental study on the response of blanket bog vegetation and water tables to ditch blocking. Wetl. Ecol. Manag. 25, 703–716.

Grigaliunas, V., Ruseckas, J., 2005. The effect of soil properties on natural forest regeneration on drained fens. Baltic Forestry 11, 75–83.

Grzywna, A., Kowalczyk-Juśko, A., 2018. The Effect of Adjustable Outflow on the Fluctuations in the Level of Surface and Ground Water. J. Ecol. Eng. 19, 159–163.

Gustafsson, M., 2005. Carbon loss after forest drainage of three peatlands in southern Sweden (MSc thesis). Department of Forest Ecology and Management, SLU, Uppsala, Sweden.

Gyimah, A., Wu, J.H., Scott, R., Gong, Y., 2020. Agricultural drainage increases the photosynthetic capacity of boreal peatlands. Agric. Ecosyst. Environ. 300, 13.

Haapalehto, T., Kotiaho, J.S., Matilainen, R., Tahvanainen, T., 2014. The effects of long-term drainage and subsequent restoration on water table level and pore water chemistry in boreal peatlands. J. Hydrol. 519, 1493–1505.

Haapalehto, T.O., Vasander, H., Jauhiainen, S., Tahvanainen, T., Kotiaho, J.S., 2011. The Effects of Peatland Restoration on Water-Table Depth, Elemental Concentrations, and Vegetation: 10 Years of Changes. Restor. Ecol. 19, 587–598.

Hedberg, P., Kotowski, W., Saetre, P., Malson, K., Rydin, H., Sundberg, S., 2012. Vegetation recovery after multiple-site experimental fen restorations. Biol. Conserv. 147, 60–67.

Hensel, B.R., Miller, M.V., 1991. Effects of wetlands creation on groundwater flow. Journal of Hydrology 126, 293–314.

Hill, A.R., Duval, T.P., 2009. Beaver dams along an agricultural stream in southern Ontario, Canada: their impact on riparian zone hydrology and nitrogen chemistry. Hydrol. Process. 23, 1324–1336.

Hillman, G.R., 1992. Some hydrological effects of peatland drainage in Alberta boreal forest. Can. J. For. Res.-Rev. Can. Rech. For. 22, 1588–1596.

Hillman, G.R., 1988. Improving wetlands for forestry in Alberta (Catalogue No. Fo 42-91/43-1988E). Canadian Forest Service and Alberta Forest Service, Edmonton, Alberta, Canada.

Hillman, G.R., Roberts, J.J., 2006. Tamarack and black spruce growth on a boreal fen in central Alberta 9 years after drainage. New For. 31, 225–243.

Holden, J., Evans, M.G., Burt, T.P., Horton, M., 2006. Impact of land drainage on peatland hydrology. Journal of Environmental Quality 35, 1764–1778.

Holden, J., Green, S.M., Baird, A.J., Grayson, R.P., Dooling, G.P., Chapman, P.J., Evans, C.D., Peacock, M., Swindles, G., 2017. The impact of ditch blocking on the hydrological functioning of blanket peatlands. Hydrol. Process. 31, 525–539.

Holden, J., Wallage, Z.E., Lane, S.N., McDonald, A.T., 2011. Water table dynamics in undisturbed, drained and restored blanket peat. J. Hydrol. 402, 103–114.

Holl, B.S., Fiedler, S., Jungkunst, H.F., Kalbitz, K., Freibauer, A., Drosler, M., Stahr, K., 2009. Characteristics of dissolved organic matter following 20 years of peatland restoration. Sci. Total Environ. 408, 78–83.

Howie, S.A., Hebda, R.J., 2018. Bog surface oscillation (mire breathing): A useful measure in raised bog restoration. Hydrol. Process. 32, 1518–1530.

Howie, S.A., Whitfield, P.H., Hebda, R.J., Munson, T.G., Dakin, R.A., Jeglum, J.K., 2009. Water Table and Vegetation Response to Ditch Blocking: Restoration of a Raised Bog in Southwestern British Columbia. Can. Water Resour. J. 34, 381–392.

Howson, T., Chapman, P.J., Shah, N., Anderson, R., Holden, J., 2021a. A comparison of porewater chemistry between intact, afforested and restored raised and blanket bogs. Sci. Total Environ. 766, 144496. <https://doi.org/10.1016/j.scitotenv.2020.144496>

Howson, T., Chapman, P.J., Shah, N., Anderson, R., Holden, J., 2021b. The effect of forest-to-bog restoration on the hydrological functioning of raised and blanket bogs. Ecohydrology 14, e2334. <https://doi.org/10.1002/eco.2334>

Hribljan, J.A., Kane, E.S., Pypker, T.G., Chimner, R.A., 2014. The effect of long-term water table manipulations on dissolved organic carbon dynamics in a poor fen peatland. J. Geophys. Res.-Biogeosci. 119, 577–595.

Hunt, L.J.H., Fair, J., Odland, M., 2018. Meadow Restoration Increases Baseflow and Groundwater Storage in the Sierra Nevada Mountains of California. Journal of the American Water Resources Association 54, 1127–1136.

Ikkonen, E.N., Kurets, V.K., Grabovik, S.I., Drozdov, S.N., 2001. The rate of carbon dioxide emission into the atmosphere from a southern Karelian mesooligotrophic bog. Russian Journal of Ecology 32, 382–385.

Iritz, L., Johansson, B., Lundin, L., 1994. Impacts of forest drainage on floods. Hydrological sciences journal 39, 637–661.

Jaatinen, K., Tuittila, E.S., Laine, J., Yrjala, K., Fritze, H., 2005. Methane-oxidizing bacteria in a Finnish raised mire complex: Effects of site fertility and drainage. Microb. Ecol. 50, 429–439.

Janzen, K., Westbrook, C.J., 2011. Hyporheic Flows Along a Channelled Peatland: Influence of Beaver Dams. Can. Water Resour. J. 36, 331–347.

Jarasius, L., Lygis, V., Sendzikaite, J., Pakalnis, R., 2015. Effect of Different Hydrological Restoration Measures in Aukstumala Raised Bog Damaged by Peat Harvesting Activities. Baltic For. 21, 192–203.

Jarveoja, J., Peichl, M., Maddison, M., Soosaar, K., Vellak, K., Karofeld, E., Teemusk, A., Mander, U., 2016. Impact of water table level on annual carbon and greenhouse gas balances of a restored peat extraction area. Biogeosciences 13, 2637–2651.

Jauhiainen, S., Laiho, R., Vasander, H., 2002. Ecohydrological and vegetational changes in a restored bog and fen. Ann. Bot. Fenn. 39, 185–199.

Java, O., Kohv, M., Lõhmus, A., 2021. Performance of a bog hydrological system dynamics simulation model in an ecological restoration context: Soomaa case study, Estonia. Water 13, 2217. <https://doi.org/10.3390/w13162217>

Johansson, B., 1993. Modelling the effects of wetland drainage on high flows (Reports Hydrology No. 8). SMHI, Norrköping, Sweden.

Karran, D.J., Westbrook, C.J., Bedard-Haughn, A., 2018. Beaver-mediated water table dynamics in a Rocky Mountain fen. Ecohydrology 11, 11.

Karu, H., Pensa, M., Room, E.I., Portsmuth, A., Triisberg, T., 2014. Carbon fluxes in forested bog margins along a human impact gradient: could vegetation structure be used as an indicator of peat carbon emissions? Wetl. Ecol. Manag. 22, 399–417.

Ketcheson, S.J., Price, J.S., 2011. The Impact of Peatland Restoration on the Site Hydrology of an Abandoned Block-Cut Bog. Wetlands 31, 1263–1274.

Koivusalo, H., Ahti, E., Lauren, A., Kokkonen, T., Karvonen, T., Nevalainen, R., Finer, L., 2008. Impacts of ditch cleaning on hydrological processes in a drained peatland forest. Hydrol. Earth Syst. Sci. 12, 1211–1227.

Komulainen, V.M., Nykanen, H., Martikainen, P.J., Laine, J., 1998. Short-term effect of restoration on vegetation change and methane emissions from peatlands drained for forestry in southern Finland. Can. J. For. Res.-Rev. Can. Rech. For. 28, 402–411.

Komulainen, V.M., Tuittila, E.S., Vasander, H., Laine, J., 1999. Restoration of drained peatlands in southern Finland: initial effects on vegetation change and CO2 balance. J. Appl. Ecol. 36, 634–648.

Kopp, B.J., Fleckenstein, J.H., Roulet, N.T., Humphreys, E., Talbot, J., Blodau, C., 2013. Impact of long-term drainage on summer groundwater flow patterns in the Mer Bleue peatland, Ontario, Canada. Hydrol. Earth Syst. Sci. 17, 3485–3498.

Koskinen, M., Maanavilja, L., Nieminen, M., Minkkinen, K., Tuittila, E.S., 2016. High methane emissions from restored Norway spruce swamps in southern Finland over one growing season. Mires Peat 17, 13.

Laine, A.M., Frolking, S., Tahvanainen, T., Tolvanen, A., Tuittila, E.S., 2019a. Spring-season flooding is a primary control of vegetation succession trajectories in primary mires. Mires Peat 24, 8.

Laine, A.M., Leppala, M., Tarvainen, O., Paatalo, M.L., Seppanen, R., Tolvanen, A., 2011. Restoration of managed pine fens: effect on hydrology and vegetation. Appl. Veg. Sci. 14, 340–349.

Laine, A.M., Mehtatalo, L., Tolvanen, A., Frolking, S., Tuittila, E.S., 2019b. Impacts of drainage, restoration and warming on boreal wetland greenhouse gas fluxes. Sci. Total Environ. 647, 169–181.

Laine, A.M., Tolvanen, A., Atalo, L.M., Tuittila, E.S., 2016. Vegetation structure and photosynthesis respond rapidly to restoration in young coastal fens. Ecol. Evol. 6, 6880–6891.

LaRose, S., Price, J., Rochefort, L., 1997. Rewetting of a cutover peatland: Hydrologic assessment. Wetlands 17, 416–423.

Lees, K.J., Artz, R.R.E., Chandler, D., Aspinall, T., Boulton, C.A., Buxton, J., Cowie, N.R., Lenton, T.M., 2021. Using remote sensing to assess peatland resilience by estimating soil surface moisture and drought recovery. Science of the Total Environment 761, 143312. <https://doi.org/10.1016/j.scitotenv.2020.143312>

Lehr, C., Poschke, F., Lewandowski, J., Lischeid, G., 2015. A novel method to evaluate the effect of a stream restoration on the spatial pattern of hydraulic connection of stream and groundwater. J. Hydrol. 527, 394–401.

Lhosmot, A., Collin, L., Magnon, G., Steinmann, M., Bertrand, C., Stefani, V., Marie‐Laure, T., Bertrand, G., 2021. Restoration and meteorological variability highlight nested water supplies in middle altitude/latitude peatlands: Towards a hydrological conceptual model of the Frasne peatland, Jura Mountains, France. Ecohydrology 14, 14:e2315. <https://doi.org/10.1002/eco.2315>

Lieffers, V.J., 1988. Sphagnum and cellulose decomposition in drained and natural areas of an Alberta peatland. Canadian Journal of Soil Science 68, 755–761.

Lindholm, T., Markkula, I., 1984. Moisture conditions in hummocks and hollows in virgin and drained sites on the raised bog Laaviosuo, southern Finland. Annales Botanici Fennici 21, 241–255.

Lode, E., 2001. Natural mire hydrology in restoration of peatland functions (Doctoral thesis). Department of Forest Soils, Swedish University of Agricultural Sciences, Uppsala, Sweden.

Loheide, S.P., Gorelick, S.M., 2007. Riparian hydroecology: A coupled model of the observed interactions between groundwater flow and meadow vegetation patterning. Water Resour. Res. 43, 16.

Luan, J.W., Liu, S.R., Wu, J.H., Wang, M., Yu, Z., 2018. The transient shift of driving environmental factors of carbon dioxide and methane fluxes in Tibetan peatlands before and after hydrological restoration. Agric. For. Meteorol. 250, 138–146.

Luan, J.W., Wu, J.H., 2015. Long-term agricultural drainage stimulates CH4 emissions from ditches through increased substrate availability in a boreal peatland. Agric. Ecosyst. Environ. 214, 68–77.

Lundin, L., 1999. Effects on hydrology and surface water chemistry of regeneration cuttings in peatland forests. International Peat Journal 9, 118–126.

Lundin, L., 1997. Effects of peat-winning on the water environment at a sedge fen ecosystem, in: Proceedings from the 10th International Peat Congress. Bremen, Germany, p. 12.

Lundin, L., 1993. Impacts of forest drainage on flow regime. Studia Forestalia Suecica; 1993. (192):22 pp. 54 ref. 192, 1–22.

Lundin, L., 1984. Torvmarksdikning Hydrologiska konsekvenser for Docksmyren (Peatland Drainage—Effects on the hydrology of the Mire Docksmyre) (Report Series A No. 1984:3). Department of Physical Geography Hydrology Division, University of Uppsala, Uppsala, Sweden.

Lundin, L., Bergquist, B., 1990. Effects on water chemistry after drainage of a bog for forestry. Hydrobiologia 196, 167–181.

Lundin, L., Lode, E., Nilsson, T., Strömgren, M., Jordan, S., Kozlov, S., 2016. Effekter vid restaurering av avslutade torvtäkter genom återvätning; undersökningar vid Porla, Toftmossen och Västkärr (Projekt, Energimyndigheten No. 7494–6). Department of Soil and Environment, Swedish University of Agricultural Sciences, Uppsala, Sweden.

Maanavilja, L., Aapala, K., Haapalehto, T., Kotiaho, J.S., Tuittila, E.S., 2014. Impact of drainage and hydrological restoration on vegetation structure in boreal spruce swamp forests. For. Ecol. Manage. 330, 115–125.

MacDonald, E., Brummell, M.E., Bieniada, A., Elliott, J., Engering, A., Gauthier, T.L., Saraswati, S., Touchette, S., Turmel-Courchesne, L., Strack, M., 2018. Using the Tea Bag Index to characterize decomposition rates in restored peatlands. Boreal Environ. Res. 23, 221–235.

Mahmood, M.S., Strack, M., 2011. Methane dynamics of recolonized cutover minerotrophic peatland: Implications for restoration. Ecol. Eng. 37, 1859–1868.

Malloy, S., Price, J.S., 2014. Fen restoration on a bog harvested down to sedge peat: A hydrological assessment. Ecol. Eng. 64, 151–160.

Maloletko, A.A., Sinyutkina, A.A., Gashkova, L.P., Kharanzhevskaya, Y.A., Magur, M.G., Voistinova, E.S., Ivanova, E.S., Chudinovskaya, L.A., Khaustova, A.A., Gordov, E., 2018. Effects of long-term drainage on vegetation, surface topography, hydrology and water chemistry of north-eastern part of Great Vasyugan Mire (Western Siberia). Presented at the International Conference and Early Career Scientists School on Environmental Observations, Modeling and Information Systems, ENVIROMIS 2018, Siberian Research Institute of Agriculture and Peat-branch, Siberian Federal Scientific Centre of Agro-BioTechnologies, Russian Academy of Sciences, Tomsk, Russia.

Mander, Ü., Lõhmus, K., Teiter, S., Uri, V., Augustin, J., 2008. Gaseous nitrogen and carbon fluxes in riparian alder stands. Boreal Environment Research 13, 231–241.

Marcotte, P., Roy, V., Plamondon, A.P., Auger, I., 2008. Ten-year water table recovery after clearcutting and draining boreal forested wetlands of eastern Canada. Hydrol. Process. 22, 4163–4172.

Marshall, S.M., 2011. The Effects of Land Use on Mineral Flat Wetland Hydrologic Processes in Lowland Agricultural Catchments (Doctoral thesis). Oregon State University, Corvallis, Oregon, USA.

Martikainen, P., Nykänen, H., Crill, P., Silvola, J., 1993. Effect of a lowered water table on nitrous oxide fluxes from northern peatlands. Nature 366, 51–53.

Martikainen, P.J., Nykänen, H., Alm, J., Silvola, J., 1995. Change in fluxes of carbon dioxide, methane and nitrous oxide due to forest drainage of mire sites of different trophy. Plant Soil 168, 571–577.

Marttila, H., Klove, B., 2010. Managing runoff, water quality and erosion in peatland forestry by peak runoff control. Ecol. Eng. 36, 900–911.

Mawby, F.J., 1995. Effects of damming peat cuttings on Glasson Moss and Wedholme Flow, two lowland raised bogs in northwest England, in: Wheeler, B.D., Shaw, S.C., Fojt, W.J., Robertson, R.A. (Eds.), Restoration of Temperate Wetlands. John Wiley and Sons, New York, NY, USA, pp. 349–357.

McCarter, C.P.R., Price, J.S., 2013. The hydrology of the Bois-des-Bel bog peatland restoration: 10 years post-restoration. Ecol. Eng. 55, 73–81.

Menberu, M.W., Haghighi, A.T., Ronkanen, A.K., Marttila, H., Klove, B., 2018. Effects of Drainage and Subsequent Restoration on Peatland Hydrological Processes at Catchment Scale. Water Resour. Res. 54, 4479–4497.

Menberu, M.W., Marttila, H., Tahvanainen, T., Kotiaho, J.S., Hokkanen, R., Move, B., Ronkanen, A.K., 2017. Changes in Pore Water Quality After Peatland Restoration: Assessment of a Large-Scale, Replicated Before-After Control-Impact Study in Finland. Water Resour. Res. 53, 8327–8343.

Menberu, M.W., Tahvanainen, T., Marttila, H., Irannezhad, M., Ronkanen, A.K., Penttinen, J., Klove, B., 2016. Water-table-dependent hydrological changes following peatland forestry drainage and restoration: Analysis of restoration success. Water Resour. Res. 52, 3742–3760.

Miller, C.A., Benscoter, B.W., Turetsky, M.R., 2015. The effect of long-term drying associated with experimental drainage and road construction on vegetation composition and productivity in boreal fens. Wetl. Ecol. Manag. 23, 845–854.

Minkkinen, K., Laine, J., 2006. Vegetation heterogeneity and ditches create spatial variability in methane fluxes from peatlands drained for forestry. Plant Soil 285, 289–304.

Minkkinen, K., Ojanen, P., Koskinen, M., Penttilä, T., 2020. Nitrous oxide emissions of undrained, forestry-drained, and rewetted boreal peatlands. For. Ecol. Manage. 478, 118494. <https://doi.org/10.1016/j.foreco.2020.118494>

Mojeremane, W., Rees, R.M., Mencuccini, M., 2010. Effects of site preparation for afforestation on methane fluxes at Harwood Forest, NE England. Biogeochemistry 97, 89–107.

Muller, F.L.L., Chang, K.C., Lee, C.L., Chapman, S.J., 2015. Effects of temperature, rainfall and conifer felling practices on the surface water chemistry of northern peatlands. Biogeochemistry 126, 343–362.

Munir, T.M., Khadka, B., Xu, B., Strack, M., 2017. Partitioning Forest‐Floor Respiration into Source Based Emissions in a Boreal Forested Bog: Responses to Experimental Drought. Forests 8, 75.

Munir, T.M., Perkins, M., Kaing, E., Strack, M., 2015. Carbon dioxide flux and net primary production of a boreal treed bog: Responses to warming and water-table-lowering simulations of climate change. Biogeosciences 12, 1091–1111.

Munir, T.M., Westbrook, C.J., 2020. Beaver dam analogue configurations influence stream and riparian water table dynamics of a degraded spring-fed creek in the Canadian Rockies. River Research and Applications 37, 330–342.

Munir, T.M., Xu, B., Perkins, M., Strack, M., 2014. Responses of carbon dioxide flux and plant biomass to water table drawdown in a treed peatland in northern Alberta: a climate change perspective. Biogeosciences 11, 807–820.

Murphy, M., Laiho, R., Moore, T.R., 2009. Effects of Water Table Drawdown on Root Production and Aboveground Biomass in a Boreal Bog. Ecosystems 12, 1268–1282.

Mustamo, P., Hyvarinen, M., Ronkanen, A.K., Klove, B., 2016. Physical properties of peat soils under different land use options. Soil Use Manage. 32, 400–410.

Mustonen, S.E., Seuna, P., 1975. Influence of forest drainage on the hydrology of an open bog in Finland. IAHS Publication 105, 519–530.

Nicia, P., Bejger, R., Sterzynska, M., Zadrozny, P., Lamorski, T., Stary, J., Parzych, P., 2018a. Restoration of hydro-ecological conditions in Carpathian forested mountain fens. Wetl. Ecol. Manag. 26, 537–546.

Nicia, P., Bejger, R., Zadrozny, P., Sterzynska, M., 2018b. The impact of restoration processes on the selected soil properties and organic matter transformation of mountain fens under Caltho-Alnetum community in the Babiogrski National Park in Outer Flysch Carpathians, Poland. J. Soils Sediments 18, 2770–2776.

Noreika, N., Kotiaho, J.S., Penttinen, J., Punttila, P., Vuori, A., Pajunen, T., Autio, O., Loukola, O.J., Kotze, D.J., 2015. Rapid recovery of invertebrate communities after ecological restoration of boreal mires. Restor. Ecol. 23, 566–579.

Nowakowska, J., Gazda, A., Tomski, A., Szwagrzyk, J., 2021. Drainage ditches enhance forest succession in a raised bog but do not affect the spatial pattern of tree encroachment. PLoS ONE 16, e0247760. <https://doi.org/10.1371/journal.pone.0247760>

Nykanen, H., Alm, J., Silvola, J., Tolonen, K., Martikainen, P.J., 1998. Methane fluxes on boreal peatlands of different fertility and the effect of long-term experimental lowering of the water table on flux rates. Glob. Biogeochem. Cycle 12, 53–69.

Paivanen, J., Ahti, E., 1988. Ditch cleaning and additional ditching in peatland forestry-effect on groundwater level. Suomen Akatemian Julkaisuja 4, 184–189.

Paivanen, J., Sarkkola, S., 2000. The effect of thinning and ditch network maintenance on the water table level in a Scots pine stand on peat soil. Suo 51, 131–138.

Palkane, M., Indriksons, A., 2012. Management and monitoring of three Latvian raised bogs and a fen, in: The Finnish Environment. Presented at the Mires from Pole to Pole. Proceedings of the XII biennial International Mire Conservation Group symposium, Finnish Environment Institute, Helsinki, Finland.

Patterson, L., Cooper, D.J., 2007. The use of hydrologic and ecological indicators for the restoration of drainage ditches and water diversions in a mountain fen, cascade range, California. Wetlands 27, 290–304.

Pearce, C., Vidon, P., Lautz, L., Kelleher, C., Davis, J., 2021. Impact of beaver dam analogues on hydrology in a semi-arid floodplain. Hydrol. Processes 35, e14275. <https://doi.org/10.1002/hyp.14275>

Pearson, M., Penttilä, T., Harjunpää, L., Laiho, R., Laine, J., Sarjala, T., Silvan, K., Silvan, N., 2015. Effects of temperature rise and water-table-level drawdown on greenhouse gas fluxes of boreal sedge fens. Boreal Environment Research 20, 489–505.

Petrone, R.M., Price, J.S., Waddington, J.M., von Waldow, H., 2004. Surface moisture and energy exchange from a restored peatland, Québec, Canada. Journal of Hydrology 295, 198–210.

Pitkänen, A., Turunen, J., Tahvanainen, T., Simola, H., 2013. Carbon storage change in a partially forestry-drained boreal mire determined through peat column inventories. Boreal Environment Research 18, 223–234.

Poyda, A., Reinsch, T., Kluss, C., Loges, R., Taube, F., 2016. Greenhouse gas emissions from fen soils used for forage production in northern Germany. Biogeosciences 13, 5221–5244.

Prevost, M., Belleau, P., Plamondon, A.P., 1997. Substrate conditions in a treed peatland: Responses to drainage. Ecoscience 4, 543–554.

Price, J., 1997. Soil moisture, water tension, and water table relationships in a managed cutover bog. J. Hydrol. 202, 21–32.

Price, J.S., Rochefort, L., Campeau, S., 2002. Use of shallow basins to restore cutover peatlands: Hydrology. Restor. Ecol. 10, 259–266.

Punttila, P., Autio, O., Kotiaho, J.S., Kotze, D.J., Loukola, O.J., Noreika, N., Vuori, A., Vepsalainen, K., 2016. The effects of drainage and restoration of pine mires on habitat structure, vegetation and ants. Silva. Fenn. 50, 31.

Purmalis, O., Grinfelde, I., Valujeva, K., Burlakovs, J., Treija, S., Skujeniece, S., 2016. The abandoned block-cut peat extraction field influence on the natural raised bog hydrological regime. Research for Rural Development 1, 236–241.

Pyatt, D.G., Anderson, A.R., Stannard, J.P., White, I.M.S., 1985. A drainage experiment on a peaty gley soil at Kershope Forest, Cumbria. Soil use and management 1, 89–94.

Qassim, S.M., Dixon, S.D., Rowson, J.G., Worrall, F., Evans, M.G., Bonn, A., 2014. A 5-year study of the impact of peatland revegetation upon DOC concentrations. J. Hydrol. 519, 3578–3590.

Raivonen, M., Makiranta, P., Lohila, A., Juutinen, S., Vesala, T., Tuittila, E.S., 2015. A simple CO2 exchange model simulates the seasonal leaf area development of peatland sedges. Ecol. Model. 314, 32–43.

Regan, S., 2020. Ecohydrology, Greenhouse Gas Dynamics and Restoration Guidelines for Degraded Raised Bogs (EPA Research Report No. 342). Environmental Protection Agency, Johnstown Castle, Co. Wexford, Ireland.

Regan, S., Flynn, R., Gill, L., Naughton, O., Johnston, P., 2019. Impacts of Groundwater Drainage on Pentland Subsidence and Its Ecological Implications on an Atlantic Raised Bog. Water Resour. Res. 55, 6153–6168.

Regina, K., Nykanen, H., Silvola, J., Martikainen, P.J., 1996. Fluxes of nitrous oxide from boreal peatlands as affected by peatland type, water table level and nitrification capacity. Biogeochemistry 35, 401–418.

Renou-Wilson, F., 2018. Network Monitoring Rewetted and Restored Peatlands/Organic Soils for Climate and Biodiversity Benefits (NEROS) (EPA Research Report No. 236). Environmental Protection Agency, Johnstown Castle, Co. Wexford, Ireland.

Renou-Wilson, F., Müller, C., Wilson, D., Moser, G., 2016. To graze or not to graze? Four years greenhouse gas balances and vegetation composition from a drained and a rewetted organic soil under grassland. Agriculture, ecosystems & environment 222, 156–170.

Ronkanen, A.-K., Irannezhad, M., Menberu, M., Marttila, H., Penttinen, J., Klöve, B., 2015. Boreal Peatland LIFE -project - Effect of restoration and drainage on peatland hydrology - A study of data before and after restoration at 46 sites in Finland (Research report). Water Resources and Environmental Engineering Research Group, University of Oulu, Oulu, Finland.

Roulet, N.T., Ash, R., Quinton, W., Moore, T., 1993. Methane flux from drained northern peatlands: Effect of a persistent water table lowering on flux. Global Biogeochemical Cycles 7, 749–769.

Roulet, N.T., Moore, T.R., 1995. The effect of forestry drainage practices on the emission of methane from northern peatlands. Can. J. For. Res.-Rev. Can. Rech. For. 25, 491–499.

Roy, V., Plamondon, A.P., Bernier, P.Y., 2000a. Draining forested wetland cutovers to improve seedling root zone conditions. Scand. J. Forest Res. 15, 58–67.

Roy, V., Ruel, J.C., Plamondon, A.P., 2000b. Establishment, growth and survival of natural regeneration after clearcutting and drainage on forested wetlands. For. Ecol. Manage. 129, 253–267.

Ruseckas, J., Grigaliūnas, V., 2008. Effect of drain‐blocking and meteorological factors on ground water table fluctuations in kamanos mire/Griovių blokavimo bei meteorologinių faktorių įtaka kamanų pelkės gruntinių vandenų slūgsojimo lygio svyravimams. Journal of Environmental Engineering and Landscape Management 16, 168–177.

Salm, J.O., Maddison, M., Tammik, S., Soosaar, K., Truu, J., Mander, U., 2012. Emissions of CO2, CH4 and N2O from undisturbed, drained and mined peatlands in Estonia. Hydrobiologia 692, 41–55.

Scamardo, J., Wohl, E., 2020. Sediment storage and shallow groundwater response to beaver dam analogues in the Colorado Front Range, USA. River Res. Appl. 36, 398–409.

Scarlett, S.J., Price, J.S., 2019. The influences of vegetation and peat properties on the hydrodynamic variability of a constructed fen, Fort McMurray, Alberta. Ecol. Eng. 139, 10.

Schimelpfenig, D.W., Cooper, D.J., Chimner, R.A., 2014. Effectiveness of Ditch Blockage for Restoring Hydrologic and Soil Processes in Mountain Peatlands. Restor. Ecol. 22, 257–265.

Secco, E.D., Haapalehto, T., Haimi, J., Meissner, K., Tahvanainen, T., 2016. Do testate amoebae communities recover in concordance with vegetation after restoration of drained peatlands? Mires and Peat 18, 1–14.

Secco, E.D., Haimi, J., Hogmander, H., Taskinen, S., Niku, J., Meissner, K., 2018. Testate amoebae community analysis as a tool to assess biological impacts of peatland use. Wetl. Ecol. Manag. 26, 597–611.

Segerros, M., 1987. Inverkan av uppdämning på grundvattenstånd (Avdelningsmeddelande (Department notice) No. 87:5). Department of Soil Science, SLU, Uppsala, Sweden.

Shantz, M.A., Price, J.S., 2006. Hydrological changes following restoration of the Bois-des-Bel Peatland, Quebec, 1999-2002. J. Hydrol. 331, 543–553.

Shuttleworth, E.L., Evans, M.G., Pilkington, M., Spencer, T., Walker, J., Milledge, D., Allott, T.E.H., 2019. Restoration of blanket peat moorland delays stormflow from hillslopes and reduces peak discharge. Journal of Hydrology X 2, 100006.

Silins, U., Rothwell, R.L., 1999. Spatial patterns of aerobic limit depth and oxygen diffusion rate at two peatlands drained for forestry in Alberta. Can. J. For. Res.-Rev. Can. Rech. For. 29, 53–61.

Silvan, N., Laiho, R., Vasander, H., 2000. Changes in mesofauna abundance in peat soils drained for forestry. Forest Ecology and Management 133, 127–133.

Simonsson, P., 1987. Skogs-och myrdikningens miljökonsekvenser: Slutrapport från ett projektområde (Environmental effects of draining wetland and forest: Final report from a group of projects) (Naturvårdsverket Rapport No. 3270). Naturvårdsverket (Swedish Environmental Protection Agency), Stockholm, Sweden.

Sinyutkina, A., 2021. Drainage consequences and self-restoration of drained raised bogs in the south-eastern part of Western Siberia: Peat accumulation and vegetation dynamics. CATENA 205, 105464. <https://doi.org/10.1016/j.catena.2021.105464>

Sorrell, B.K., Partridge, T.R., Clarkson, B.R., Jackson, R.J., Chagué-Goff, C., Ekanayake, J., Payne, J., Gerbeaux, P., Grainger, N.P.J., 2007. Soil and vegetation responses to hydrological manipulation in a partially drained polje fen in New Zealand. Wetlands Ecol. Manage. 15, 361–383.

Sparacino, M.S., Rathburn, S.L., Covino, T.P., Singha, K., Ronayne, M.J., 2019. Form-based river restoration decreases wetland hyporheic exchange: Lessons learned from the Upper Colorado River. Earth Surf. Process. Landf. 44, 191–203.

Stewart, A.J.A., Lance, A.N., 1991. Effects of moor-draining on the hydrology and vegetation of Northern Pennine blanket bog. J. Appl. Ecol. 28, 1105–1117.

Strack, M., Cagampan, J., Fard, G.H., Keith, A.M., Nugent, K., Rankin, T., Robinson, C., Strachan, I.B., Waddington, J.M., Xu, B., 2016. Controls on plot-scale growing season CO2 and CH4 fluxes in restored peatlands: Do they differ from unrestored and natural sites? Mires Peat 17, 18.

Strack, M., Keith, A.M., Xu, B., 2014. Growing season carbon dioxide and methane exchange at a restored peatland on the Western Boreal Plain. Ecol. Eng. 64, 231–239.

Strack, M., Munir, T.M., Khadka, B., 2019. Shrub abundance contributes to shifts in dissolved organic carbon concentration and chemistry in a continental bog exposed to drainage and warming. Ecohydrology 12, 12.

Strack, M., Waddington, J.M., Bourbonniere, R.A., Buckton, E.L., Shaw, K., Whittington, P., Price, J.S., 2008. Effect of water table drawdown on peatland dissolved organic carbon export and dynamics. Hydrol. Process. 22, 3373–3385.

Strack, M., Waddington, J.M., Rochefort, L., 2006a. Response of vegetation and net ecosystem carbon dioxide exchange at different peatland microforms following water table drawdown. Journal of Geophysical Research 111, G02006. <https://doi.org/10.1029/2005JG000145>

Strack, M., Waddington, J.M., Tuittila, E.S., 2004. Effect of water table drawdown on northern peatland methane dynamics: Implications for climate change. Glob. Biogeochem. Cycle 18, 7.

Strack, M., Waller, M.F., Waddington, J.M., 2006b. Sedge succession and peatland methane dynamics: A potential feedback to climate change. Ecosystems 9, 278–287.

Strack, M., Zuback, Y., McCarter, C., Price, J., 2015. Changes in dissolved organic carbon quality in soils and discharge 10 years after peatland restoration. J. Hydrol. 527, 345–354.

Swindles, G.T., Green, S.M., Brown, L., Holden, J., Raby, C.L., Turner, T.E., Smart, R., Peacock, M., Baird, A.J., 2016. Evaluating the use of dominant microbial consumers (testate amoebae) as indicators of blanket peatland restoration. Ecol. Indic. 69, 318–330.

Tauchnitz, N., Osterloh, K., Bernsdorf, S., Meissner, R., Kison, H.U., 2010. Changes of the water and nutrient dynamics of a drained mire in the German Harz Mountains after rewetting. Telma 229–244.

Tuittila, E.S., Vasander, H., Laine, J., 2000. Impact of rewetting on the vegetation of a cut-away peatland. Appl. Veg. Sci. 3, 205–212.

Urbanova, Z., Barta, J., Picek, T., 2013a. Methane Emissions and Methanogenic Archaea on Pristine, Drained and Restored Mountain Peatlands, Central Europe. Ecosystems 16, 664–677.

Urbanova, Z., Picek, T., Tuittila, E.S., 2013b. Sensitivity of carbon gas fluxes to weather variability on pristine, drained and rewetted temperate bogs. Mires Peat 11, 14.

Van Seters, T.E., Price, J.S., 2002. Towards a conceptual model of hydrological change on an abandoned cutover bog, Quebec. Hydrol. Process. 16, 1965–1981.

Vasander, H., Leivo, A., Tanninen, T., 1992. Rehabilitation of a drained peatland area in the Seitseminen national park in southern Finland, in: Bragg, O.M., Hulme, P.D., Ingram, H.A.P., Robertson, R.A. (Eds.), Peatland Ecosystems and Man: An Impact Assesment. International Peat Society and Department of Biological Sciences, University of Dundee, Dundee, Scotland, pp. 381–387.

Venterink, H.O., Kardel, I., Kotowski, W., Peeters, W., Wassen, M.J., 2009. Long-term effects of drainage and hay-removal on nutrient dynamics and limitation in the Biebrza mires, Poland. Biogeochemistry 93, 235–252.

Vickery, E.J., 2006. Monitoring Peatland Damage and Restoration Using Testate Amoebae as Indicator Organisms (Doctoral thesis). University of Plymouth, Plymouth, UK.

Vitovcova, K., Liparova, J., Manukjanova, A., Vasutova, M., Vrba, P., Prach, K., 2021. Biodiversity restoration of formerly extracted raised bogs: vegetation succession and recovery of other trophic groups. Wetlands Ecology and Management. <https://doi.org/10.1007/s11273-021-09847-z>

Vogt, J., Wu, J.H., Altdorff, D., Le, T.B., Gong, Y., 2020. Nitrous oxide fluxes of a boreal abandoned pasture do not significantly differ from an adjacent natural bog despite distinct environmental conditions. Sci. Total Environ. 714, 11.

von Arnold, K., Nilsson, M., Hanell, B., Weslien, P., Klemedtsson, L., 2005. Fluxes of CO2, CH4 and N2O from drained organic soils in deciduous forests. Soil Biology & Biochemistry 37, 1059–1071.

Von Arnold, K., Weslien, P., Nilsson, M., Svensson, B.H., Klemedtsson, L., 2005. Fluxes of CO2, CH4 and N2O from drained coniferous forests on organic soils. For. Ecol. Manage. 210, 239–254.

Waddington, J.M., Strack, M., Greenwood, M.J., 2010. Toward restoring the net carbon sink function of degraded peatlands: Short-term response in CO2 exchange to ecosystem-scale restoration. J. Geophys. Res.-Biogeosci. 115, 13.

Waddington, J.M., Toth, K., Bourbonniere, R., 2008. Dissolved organic carbon export from a cutover and restored peatland. Hydrological Processes 22, 2215–2224.

Wallage, Z.E., Holden, J., 2011. Near-surface macropore flow and saturated hydraulic conductivity in drained and restored blanket peatlands. Soil Use Manage. 27, 247–254.

Westbrook, C.J., Cooper, D.J., Baker, B.W., 2006. Beaver dams and overbank floods influence groundwater-surface water interactions of a Rocky Mountain riparian area. Water Resour. Res. 42, 12.

Whittington, P.N., Price, J.S., 2006. The Effects of Water Table Draw-Down (as a Surrogate for Climate Change) on the Hydrology of a Fen Peatland, Canada. Hydrological Processes 20, 3589.

Wilson, D., Farrell, C.A., Fallon, D., Moser, G., Muller, C., Renou-Wilson, F., 2016. Multiyear greenhouse gas balances at a rewetted temperate peatland. Glob. Change Biol. 22, 4080–4095.

Wilson, L., Wilson, J., Holden, J., Johnstone, I., Armstrong, A., Morris, M., 2011. The impact of drain blocking on an upland blanket bog during storm and drought events, and the importance of sampling-scale. J. Hydrol. 404, 198–208.

Wilson, L., Wilson, J., Holden, J., Johnstone, I., Armstrong, A., Morris, M., 2010. Recovery of water tables in Welsh blanket bog after drain blocking: Discharge rates, time scales and the influence of local conditions. J. Hydrol. 391, 377–386.

Worrall, F., Armstrong, A., Holden, J., 2007. Short-term impact of peat drain-blocking on water colour, dissolved organic carbon concentration, and water table depth. J. Hydrol. 337, 315–325.

Yamulki, S., Anderson, R., Peace, A., Morison, J.I.L., 2013. Soil CO2 CH4 and N2O fluxes from an afforested lowland raised peatbog in Scotland: implications for drainage and restoration. Biogeosciences 10, 1051–1065.

Zhang, X.H., Liu, H.Y., Baker, C., Graham, S., 2012. Restoration approaches used for degraded peatlands in Ruoergai (Zoige), Tibetan Plateau, China, for sustainable land management. Ecol. Eng. 38, 86–92.

Zhou, W., Cui, L., Wang, Y., Li, W., Kang, X., 2021. Carbon emission flux and storage in the degraded peatlands of the Zoige alpine area in the Qinghai–Tibetan Plateau. Soil Use Manage. 37, 72–82.

Zhou, W.C., Cui, L.J., Wang, Y.F., Li, W., 2017. Methane emissions from natural and drained peatlands in the Zoige, eastern Qinghai-Tibet Plateau. J. For. Res. 28, 539–547.
