# Supplementary material for: Effects on groundwater storage of restoring, constructing or draining wetlands in temperate and boreal climates: a systematic review
Source: Environ Evid. 2022 Dec 8;11:38. doi: 10.1186/s13750-022-00289-5 (PMC11378806; doi:10.1186/s13750-022-00289-5)
Supplement: Supplementary file 6 — Additional file 6. Details on data extraction strategy. [file 13750_2022_289_MOESM6_ESM.docx]

Additional file 6. Details on data extraction strategy

## Dealing with non-independence

A meta-analysis rests on the assumption of independent data in the studies that are included. In wetland research, non-independence can arise if, for example, multiple plots in a study sample groundwater levels at the same wetland intervention. The samples are then not independent as they essentially measure the same intervention.

Our principal approach to dealing with this type of non-independence was to prefer true replicates of wetland interventions, that is, studies of separate wetlands where the same intervention was introduced in places that were not related to each other. In addition to this, we used sensitivity analysis to investigate the influence of allowing pseudoreplicated designs. Our approach of using true replication before pseudoreplication was a conservative approach and ensures we did not artificially inflate the *N* in a study.

Another possible source of non-independence is common controls against multiple interventions. This was typically not a problem in our analysis, as there was usually only one type of intervention that could be measured against a particular control. For example, a group of restored wetlands was only measured against a group of drained wetlands. No other interventions were measured with the drained wetland as control.

Yet another possible source of non-independence is multiple outcomes measured for the same sample. This was also not an issue in our investigation, as we only measured a single outcome (groundwater level) per study.

Non-independence can also arise if multiple studies are part of the same article. When we have included multiple studies from the same article, the studies were performed in physically and distinctly separate wetlands or parts of wetlands that we judged to be hydrologically independent.

## Handling of replication for studies using raw mean difference

Studies had to report spatial replication to be included in data extraction for meta-analysis. We accepted both true replication across different wetlands and spatial pseudoreplication across different parts of the same wetland. When possible, we preferred studies with true replicates. That is, when the same wetland was included both in a study with pseudoreplication (e.g., multiple plots, microtopography units, or vegetation types, or multiple distances from intervention reported) and a study with true replication (i.e., as one of several separate wetlands investigated), we included only the true replication study and excluded the other study from meta-analysis. In some instances, data for a particular wetland were combined from several articles.

Spatial pseudoreplication can be difficult to distinguish from true replication. A strict separation into two categories is often not meaningful. Instead, study designs can be thought of as ranging from fully pseudoreplicated to fully replicated.

As an example of the former (spatial pseudoreplication), consider a single wetland with multiple sampling plots in a limited area (see Figure 1). Often, the sampling plots are allocated in this way to investigate some other phenomenon than groundwater storage in the wetland (for example, effects on vegetation or insects), and for this purpose, the replication scale may be adequate. However, for groundwater changes, only a single intervention (for example, ditch blocking in the single ditch in the illustration) is present, and therefore the intervention is not replicated. True replication requires that different ditches are blocked in different wetlands, and that these wetlands are not hydrologically connected to each other (Figure 1, right side). Often, there is as noted a continuum, with separate ditches in the same wetland being blocked, or with different sampling areas in the same wetland increasingly acquiring the properties of a true replication as distances increase.


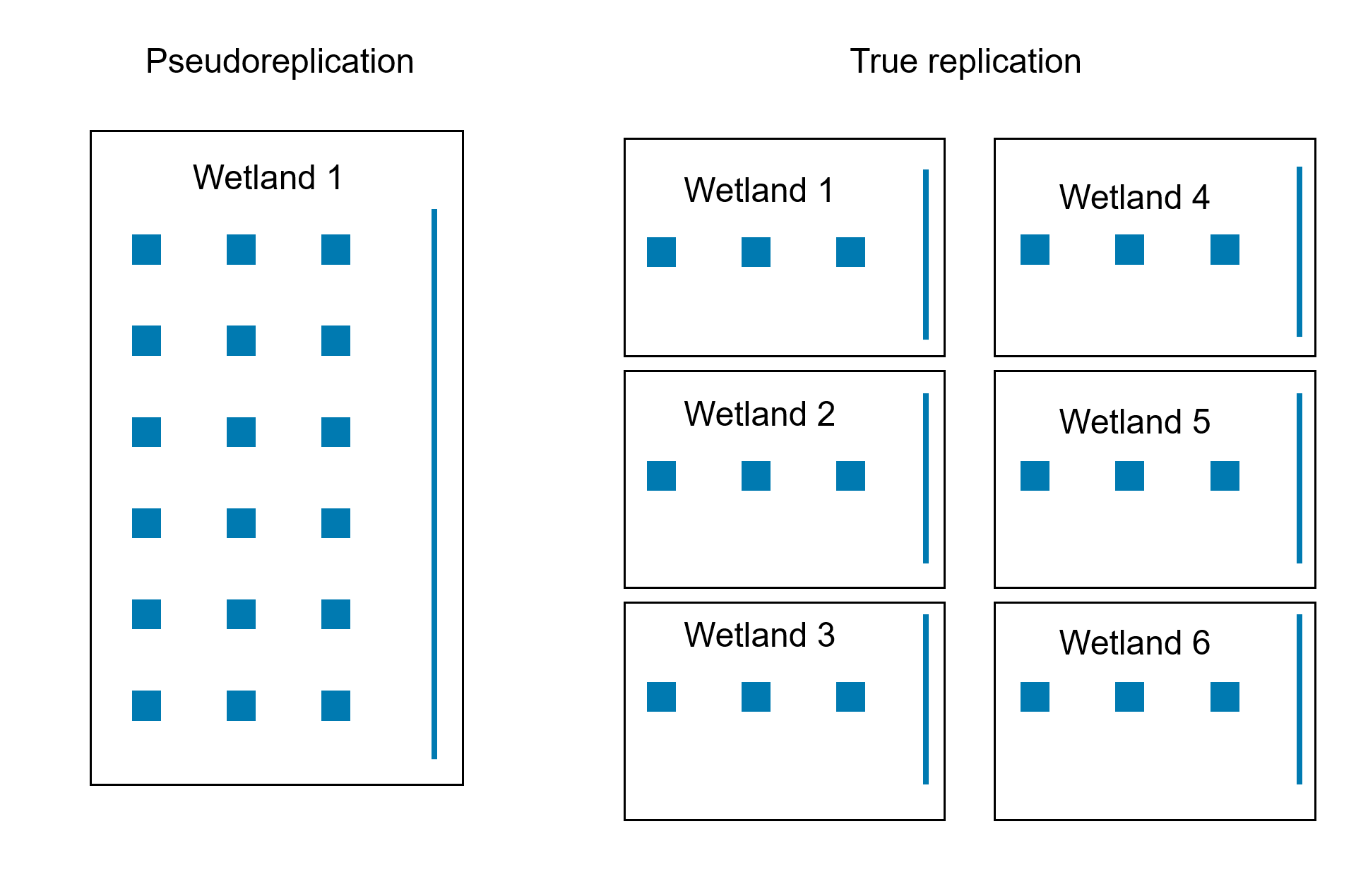


Figure 1. Two examples of study designs. Left: sampling plots are spatially pseudoreplicated (n = 18) at a single ditched site. Right: sampling is truly replicated (n = 6, each with 3 sampling plots) across multiple independently ditched sites.

In our analysis, we judged the degree of separation at the intervention level to decide whether the study was truly replicated or only spatially pseudoreplicated. Both pseudoreplicated and truly replicated studies often involve variations in effect modifiers between replicates, which sometimes limited the possibility to investigate moderators. For example, it was common that spatial pseudoreplicates differed in distance to the intervention, or that true replicates from different wetlands varied in some hydrologically important characteristic, such as peat depth, vegetation age, wetland type, or degree of peat decomposition.

Following (Haddaway et al., 2014), we did not accept temporal pseudoreplication as the basis of variability in meta-analysis. This is because such replication only samples the variability over sampling occasions, that is, due to varying weather conditions over multiple years or seasons.

## Handling of replication for groundwater level changes at different distances

In the meta-analysis of changes in groundwater level at different distances from the intervention, the effect sizes were the slope and the intercept parameters of the relationship, as described in the main text. The *N* for each study was the number of sampling points used to estimate the relationship (a minimum of three were required to fit a logarithmic equation). The standard error for each effect size was the standard error of the estimate of the corresponding parameter, as provided by the LINEST function in Excel.

In most cases, sampling was carried out at different distances along a single transect. In some cases, data were reported as averages of multiple transects. Both these types of studies were handled in the same way, with *N* always representing the number of sampling distances used to fit the regression. Actual changes in groundwater levels, i.e., the difference between treatment and control, were calculated for each sampling distance, and the curve was fit to these values and the logarithm of the corresponding distances (see example in Figure 2).


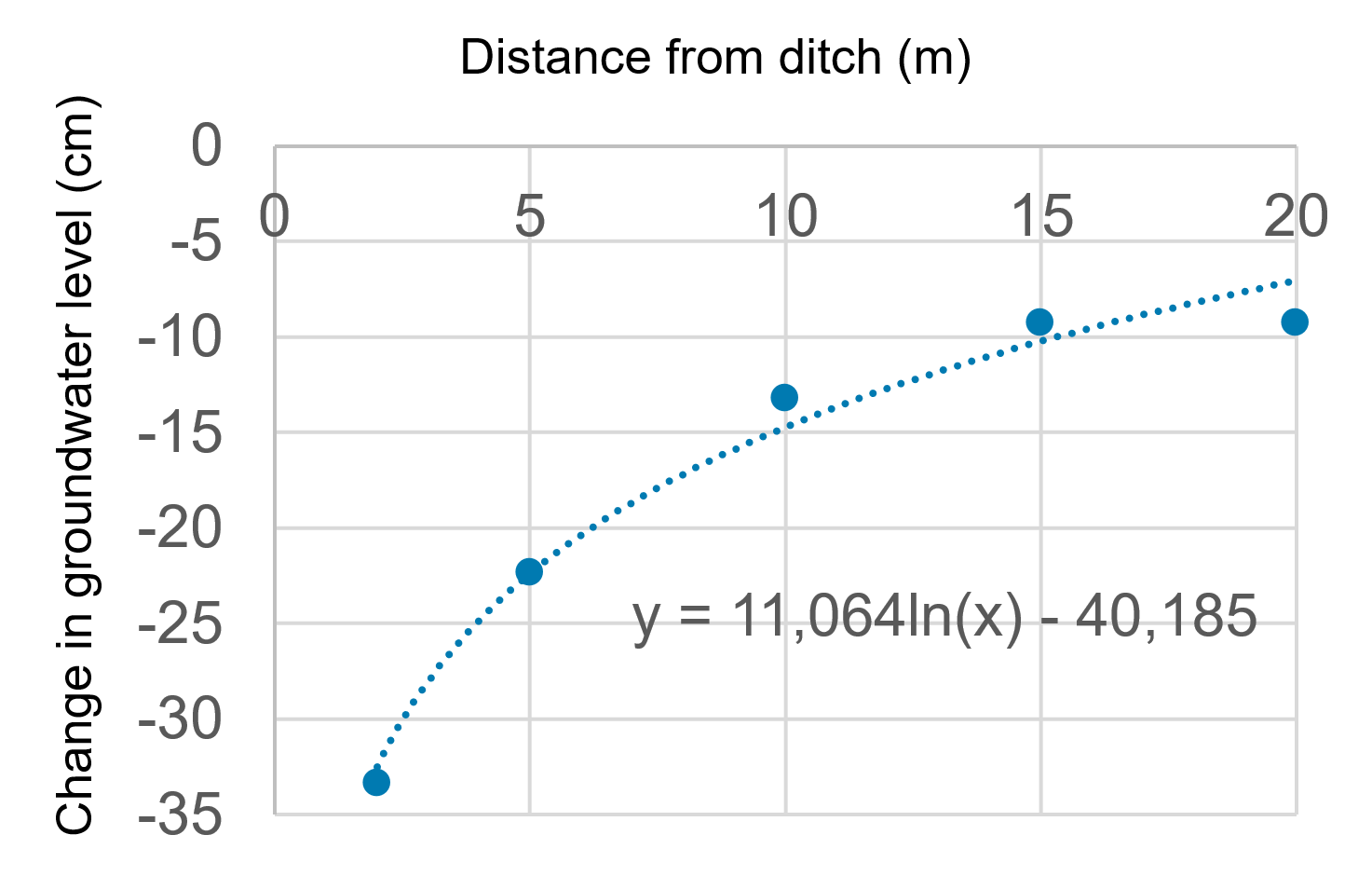


*Figure 2. Example of fitting the function y = mx + b to data from a drainage study. The N used from this type of study is the number of sampling distances, in this case 5.*

## Data extraction procedure

For overall wetland effect sizes, we extracted data according to the following procedure. If effect sizes (average change in groundwater levels) were available in tables for separate wetlands, or distinctly separated parts of a wetland complex, these values were used to calculate a mean change and standard error across the *N* true replications in the study. If there was no true replication in the study (i.e., a study of a single wetland), spatial pseudoreplication was accepted for *N* separate sampling plots, ditch spacing areas, vegetation types, microtopography units, or similar for single wetlands. If both true replication across different wetlands and spatial pseudoreplication within wetlands was possible, we calculated the true replicate effect across wetlands.

If control and intervention sites were pairwise matched, differences were calculated per pair and then averaged. The standard error for the study was then simply the standard error of the set of differences.

If control and intervention sites were not paired, the groupwise average difference

$$D=X_{1}-X_{2}$$

was calculated, where *X_1_* and *X_2_* are the means of the intervention and the control group, respectively. The standard error of the difference, *SE_D_*, was calculated as

$${SE}_{D}= \sqrt{V_{D}}$$

where

$$V_{D}=\frac{n_{1}+n_{2}}{n_{1}n_{2}}S_{pooled}^{2}$$

with *S_pooled_* the pooled standard deviations of the groups, and *n_1_* and *n_2_* the number of observations in groups 1 and 2. *S_pooled_* is calculated from the corresponding group standard deviations *S_1_* and *S_2_* as

$$S_{pooled}= \sqrt{\frac{\left( n_{1}-1 \right)S_{1}^{2}+\left( n_{2}-1 \right)S_{2}^{2}}{n_{1}+n_{2}-2}}.$$

Multiple studies could occur in the same article. If data were reported for clearly distinct investigations, such as site types (e.g., organic and mineral soils) or treatments (e.g., groups of wetlands restored five or ten years earlier), they were coded as separate studies in the meta-analysis table. This required that we judged the investigated areas to be physically separate enough for them to be hydrologically independent. The differing factor between multiple studies in the same article is indicated in the treatment code, which is listed in the columns “Raw mean difference Treatment ID” and “Regression Treatment ID”, respectively, in Additional File 5. If multiple wetlands were reported separately as control areas, they were used as a common control group, unless it was clear that they served as distinct controls for separate intervention sites.

If data were presented for distinct points in time, for example as monthly data, effects were first calculated separately for each time and then averaged, if sampling was simultaneous for control and intervention. If sampling was not simultaneous, effects were calculated for averages over each year and then averaged across years. We included only the part of the year that was common to all years. However, when data were presented for similar periods (e.g., the snow-free season), we included all data even if there were slight variations in sampling times between years. The average across time steps (a weighted average if time steps were of different length) was used as the effect size for each replication, and the average across replications as the effect size for the study, with *N* and the standard error based on the number of replications and the variability between them.

For Before-After studies, the measurement periods had to be the same part of the year to be included in the meta-analysis. That is, we have excluded studies that compare pre-treatment summer data with post-treatment fall data from the meta-analysis. Since studies from very short samples of the summer season, e.g., with data from a single day or a few storms, were judged to be less representative, we required studies to include at least one data point from each of the months June, July and August (December, January and February in the Southern hemisphere) to be eligible for meta-analysis.

If data were available in tables or in the main body of the article, we extracted the data directly from text. If data were available in figures, we digitized them using the online tool WebPlotDigitizer v4.5 (Rohatgi, 2021) and extracted data from the resulting csv output. In the main metadata table (Additional File 5), the column titled “Data extraction method” shows which alternative was used for each study. It was rare that studies published directly usable information together with information on *N* and variability in tables. Therefore, for most studies, we calculated the means and standard errors across the reported replicates as described above.

## References

Haddaway, N.R., Burden, A., Evans, C.D., Healey, J.R., Jones, D.L., Dalrymple, S.E., Pullin, A.S., 2014. Evaluating effects of land management on greenhouse gas fluxes and carbon balances in boreo-temperate lowland peatland systems. Environ. Evid. 3, 5. https://doi.org/10.1186/2047-2382-3-5

Rohatgi, A., 2021. WebPlotDigitizer. Version 4.5. Available at https://automeris.io/WebPlotDigitizer/.
