# Supplementary material for: Effects on groundwater storage of restoring, constructing or draining wetlands in temperate and boreal climates: a systematic review
Source: Environ Evid. 2022 Dec 8;11:38. doi: 10.1186/s13750-022-00289-5 (PMC11378806; doi:10.1186/s13750-022-00289-5)
Supplement: Supplementary file 8 — Additional file 8. Discussion of moderator tests and sensitivity analyses. [file 13750_2022_289_MOESM8_ESM.docx]

Additional file 8. Discussion of selected moderator tests and sensitivity analyses

In this file we discuss the results of some of the tests of moderators and sensitivity tests. Results are reported separately for restoration and drainage, and for effects at different distances versus overall effects. The full results of all statistical tests, for the complete set of moderators and sensitivity analyses, are available in Additional file 9.

# Restoration effects at different distances

For studies that reported the restoration effect at different distances (n = 8), results were so few that it is hard to draw any conclusions about the influence of the restoration intervention magnitude (Figure 1 and Figure 2).


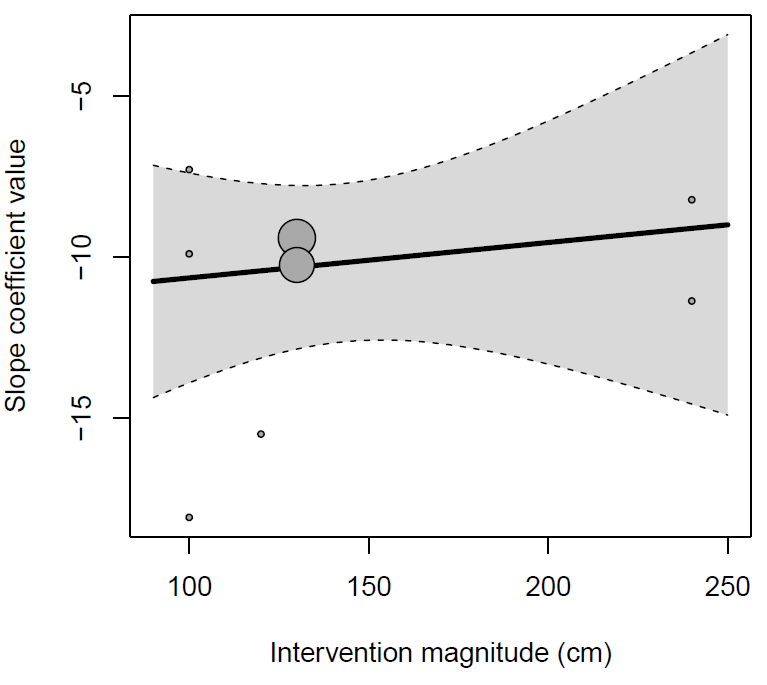


Figure 1. Meta-regression of the slope of the effect size vs distance relationship against the restoration intervention magnitude (n = 8). Size of symbols represent the study weight in the meta-analysis (proportional to the number of wetlands in the original study).


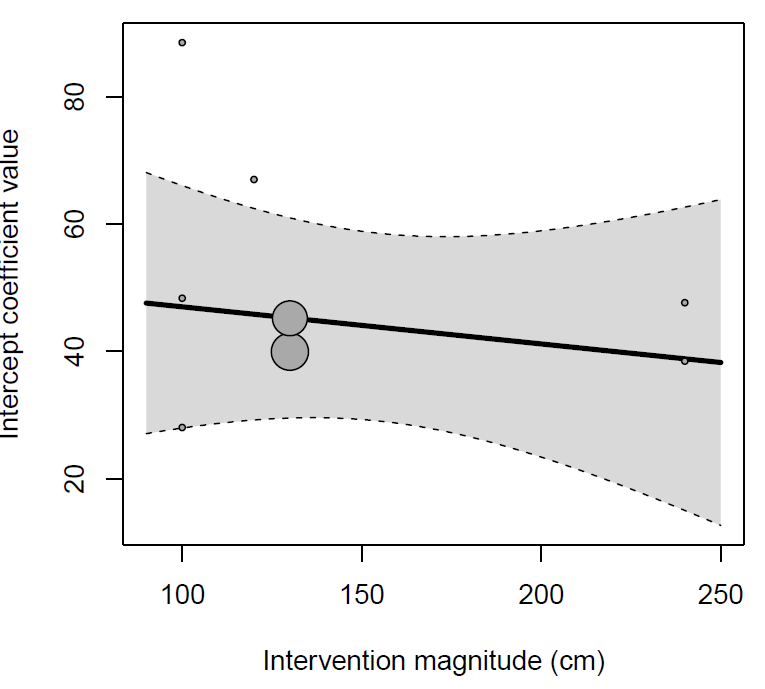


Figure 2. Meta-regression of the intercept of the effect size vs distance relationship against the restoration intervention magnitude (n = 8). Size of symbols represent the study weight in the meta-analysis (proportional to the number of wetlands in the original study).

# Overall restoration effects

The intervention magnitude is not a significant moderator (n = 26), even if removing outliers at 240 cm (n = 24 remaining) and if considering only peatlands without blanket peatlands (n = 20 remaining).


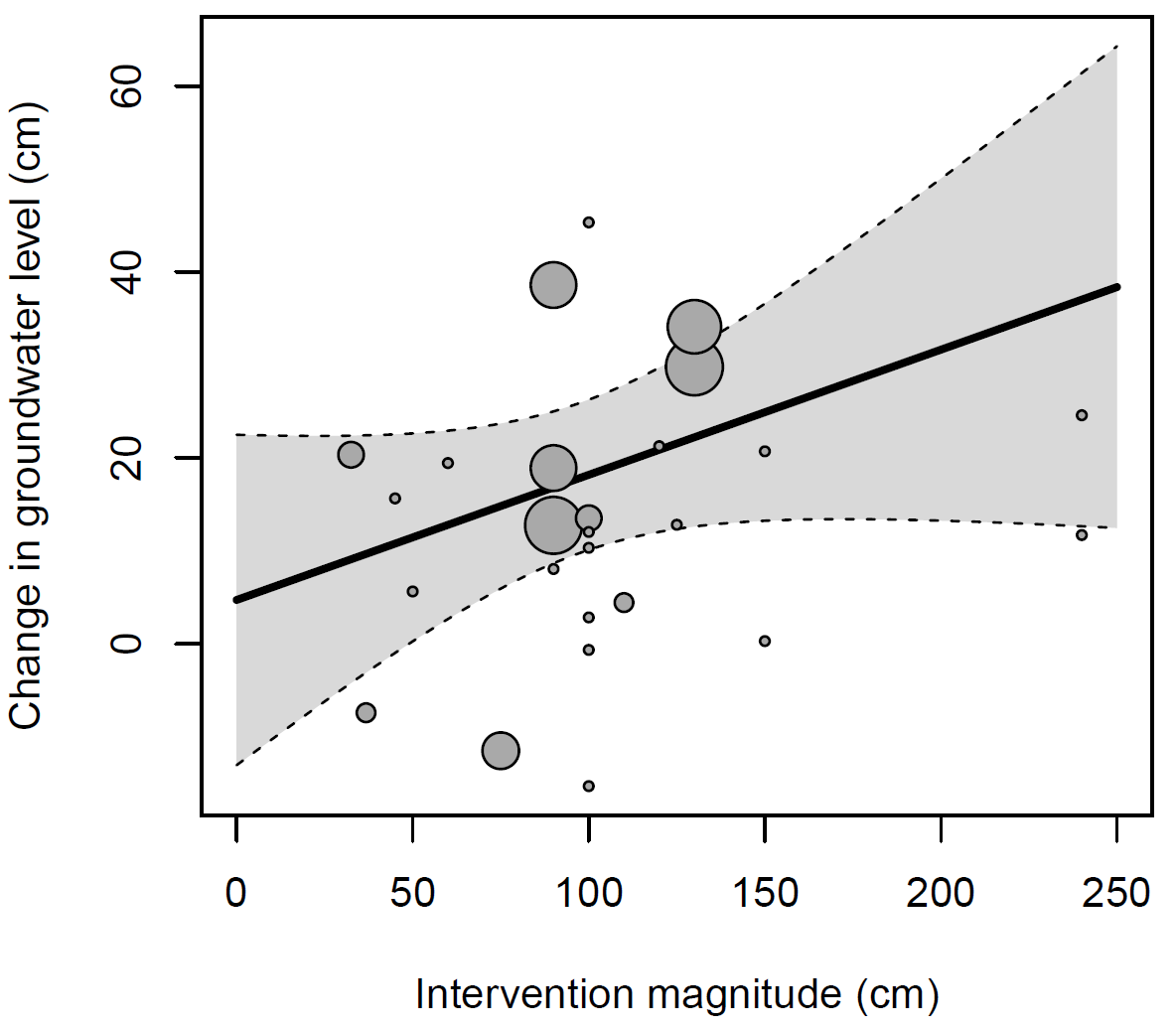


Figure 3. Meta-regression of the effect size against the restoration intervention magnitude for all studies (n = 26). Size of symbols represent the study weight in the meta-analysis (proportional to the number of wetlands in the original study).


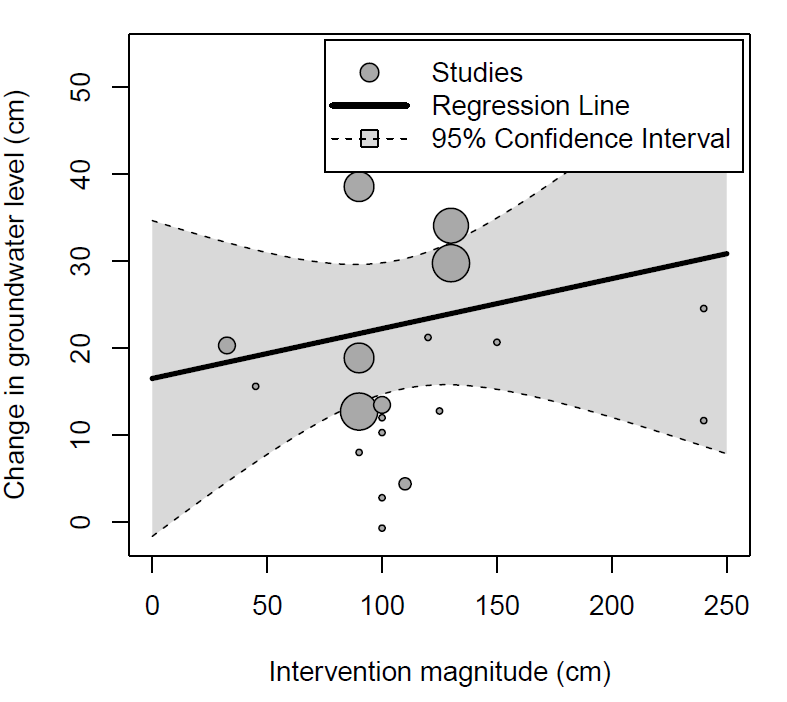


Figure 4. Meta-regression of the effect size against the restoration intervention magnitude for peatland studies that are not blanket peatlands (n = 20). Size of symbols represent the study weight in the meta-analysis (proportional to the number of wetlands in the original study).

As noted in the main text, the maximum distance of groundwater level sampling is a significant moderator (p = 0.02), for all studies that are not blanket peatlands (n = 10, Figure 5).


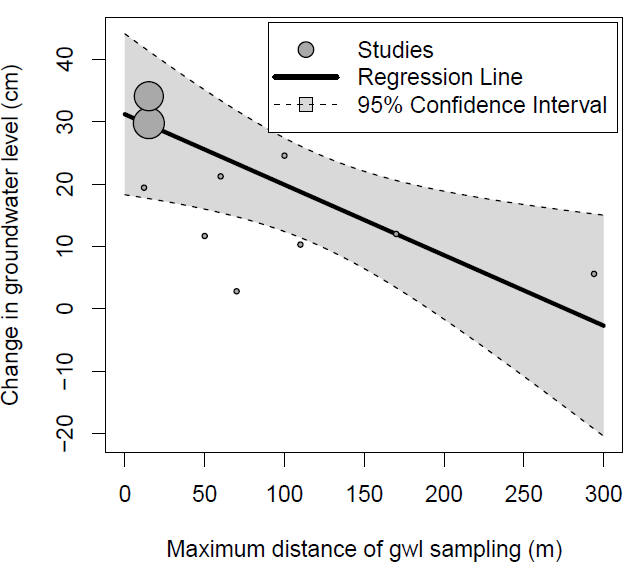


Figure 5. Meta-regression of the effect size against the maximum distance of groundwater sampling for all restoration studies that are not blanket peatlands (n = 10). Size of symbols represent the study weight in the meta-analysis (proportional to the number of wetlands in the original study).

However, if reducing the test to only peatlands except blanket peatlands (n = 8), the effect is no longer significant (p = 0.07, Figure 6).


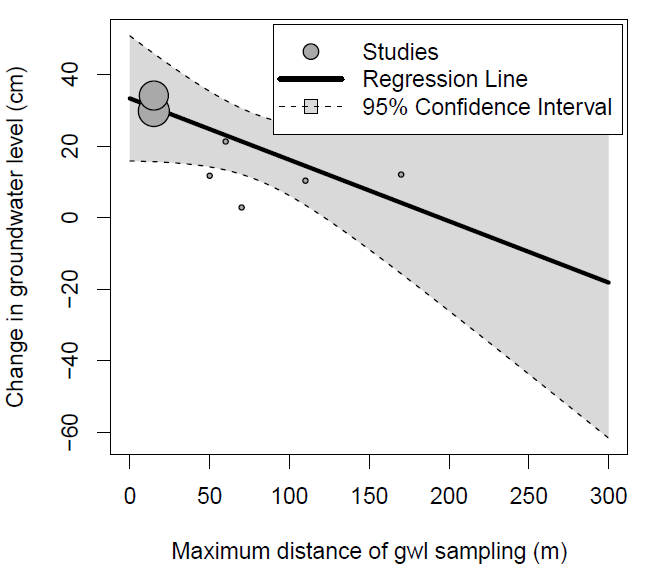


Figure 6. Meta-regression of the effect size against the maximum distance of groundwater sampling for all peatland restoration studies that are not blanket peatlands (n = 8). Size of symbols represent the study weight in the meta-analysis (proportional to the number of wetlands in the original study).

Moderators related to the study design (not significant, Figure 7 and Figure 8):


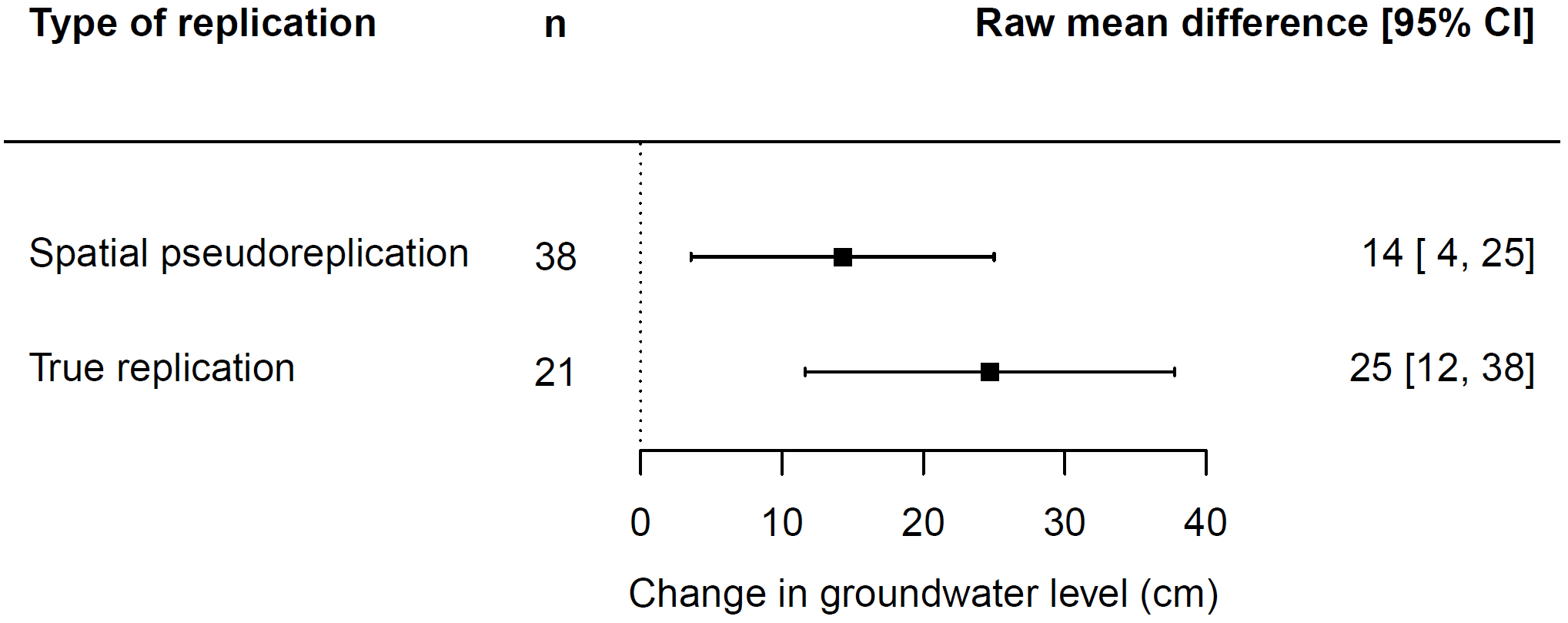


Figure 7. Average effect sizes and confidence intervals for different types of replication, for overall effects in the wetland in the vicinity of the restoration (n = 59).


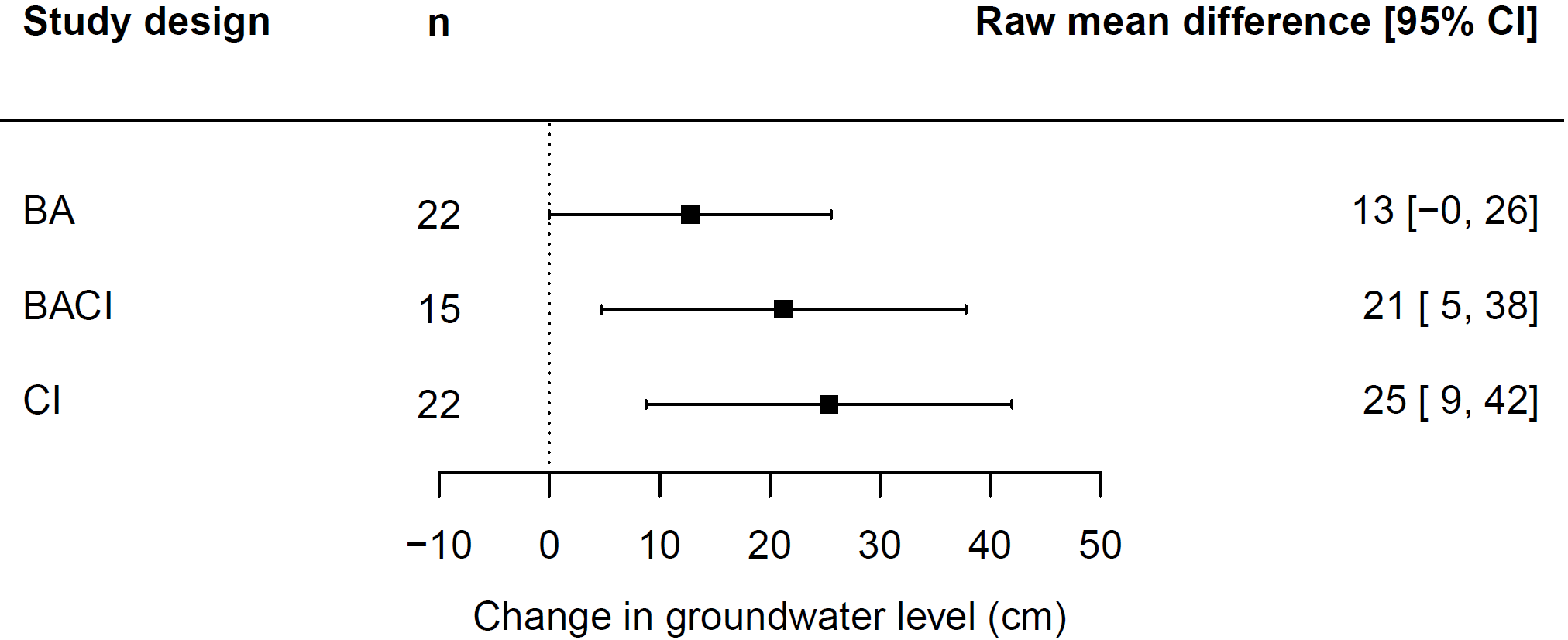


Figure 8. Average effect sizes and confidence intervals for different types of study designs, for overall effects in the wetland in the vicinity of the restoration (n = 59).

Cumulative meta-analysis effect size calculated from adding one restoration study at a time in chronological order (Figure 9). This analysis shows that the point estimate does not change much with the addition of the last third of studies, but there is some change in the width of the confidence interval which becomes slightly narrower with time.


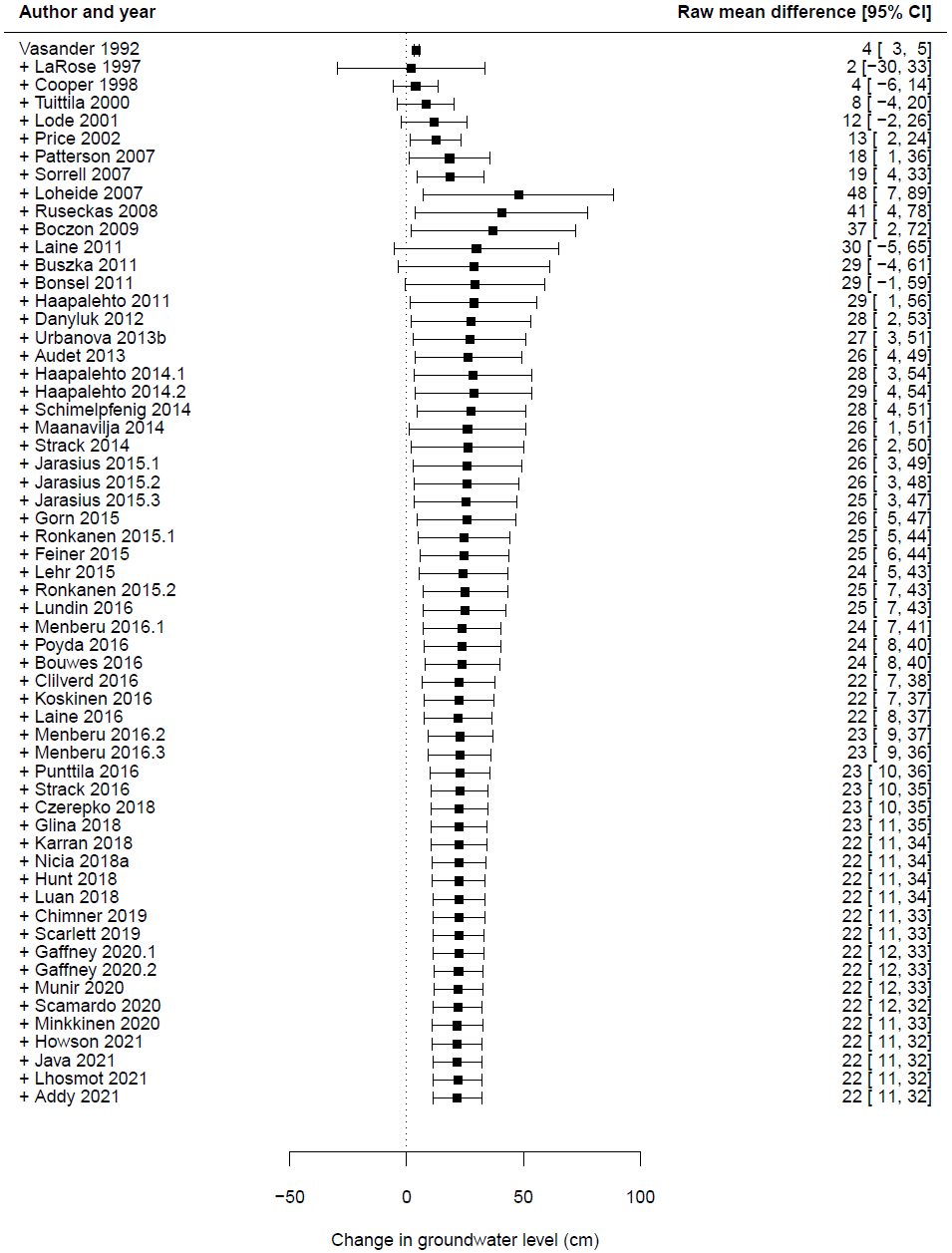


Figure 9. Average effect sizes and confidence intervals based on an incremental addition of each study in chronological order, for overall effects in the wetland in the vicinity of the restoration (n = 59).

# Drainage effects at different distances

The depth of the drainage intervention was not a significant moderator in the studies of restoration effects at different distances (n = 33, Figure 10 and Figure 11).
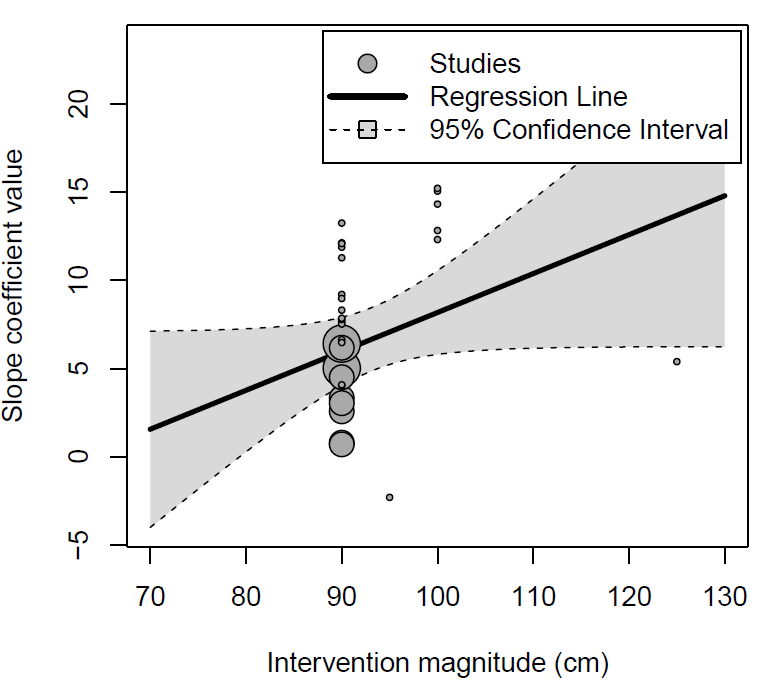


Figure 10. Meta-regression of the slope of the effect size vs distance relationship against the drainage intervention magnitude (n = 33). Size of symbols represent the study weight in the meta-analysis (proportional to the number of wetlands in the original study).


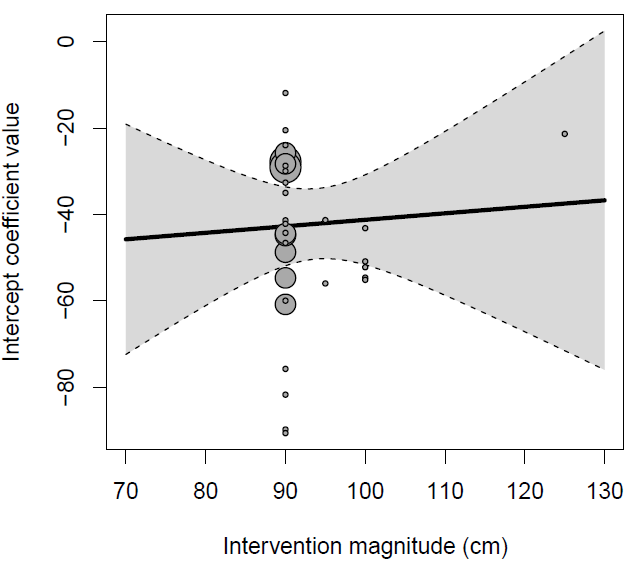


Figure 11. Meta-regression of the intercept of the effect size vs distance relationship against the drainage intervention magnitude (n = 33). Size of symbols represent the study weight in the meta-analysis (proportional to the number of wetlands in the original study).

Leaving out the outlier at 125 cm depth yields a highly significant moderator test for the slope coefficient (p < 0.001, Figure 12) but not the intercept. However, as most studies are clustered at 90 cm, there is a limited range of values for the moderator.


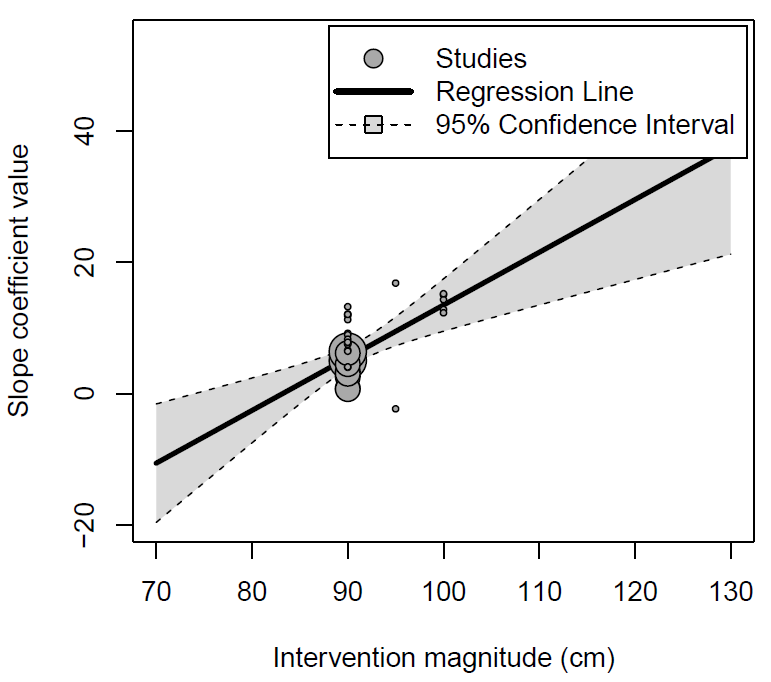


Figure 12. Meta-regression of the slope of the groundwater level change vs distance relationship against the drainage intervention magnitude, excluding the outlier at 125 cm depth (n = 32). Size of symbols represent the study weight in the meta-analysis (proportional to the number of wetlands in the original study).

We did see a significant difference between different types of references for the groundwater level sampling (n = 35). Effects tended to be larger when referenced against a fixed point or common datum, compared to when referenced against the ground surface (Figure 13). This is in line with the ground surface subsiding in peatlands, so that effects would tend to be increasing more rapidly with distance (i.e., a greater value of the slope coefficient) as seen here when evaluated against the original ground surface level or fixed point. However, for other meta-analyses, this moderator was not significant.


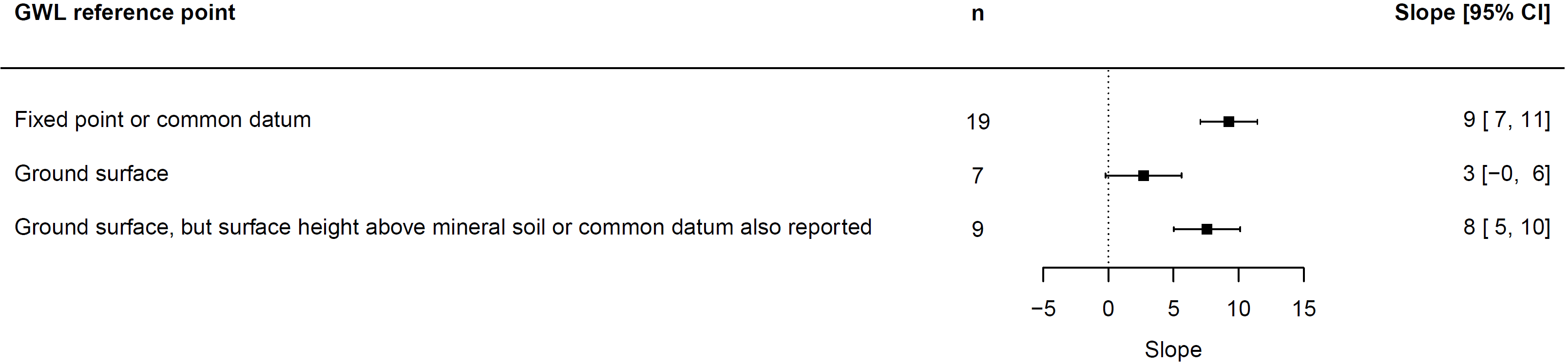


Figure 13. Average effect sizes and confidence intervals for different types of references for the groundwater level sampling (n = 35), for the slope of the groundwater level change vs distance relationship.

The moderator test for transect type for the intercept parameter was significant (p = 0.04, n = 35), indicating that open-ended transects are associated with groundwater drawdown that is on average 19 cm smaller in magnitude than drawdown at closed transects between ditches. This direction is expected as confined transects should be exposed to more intense drainage. However, confidence intervals still overlap (Figure 14).


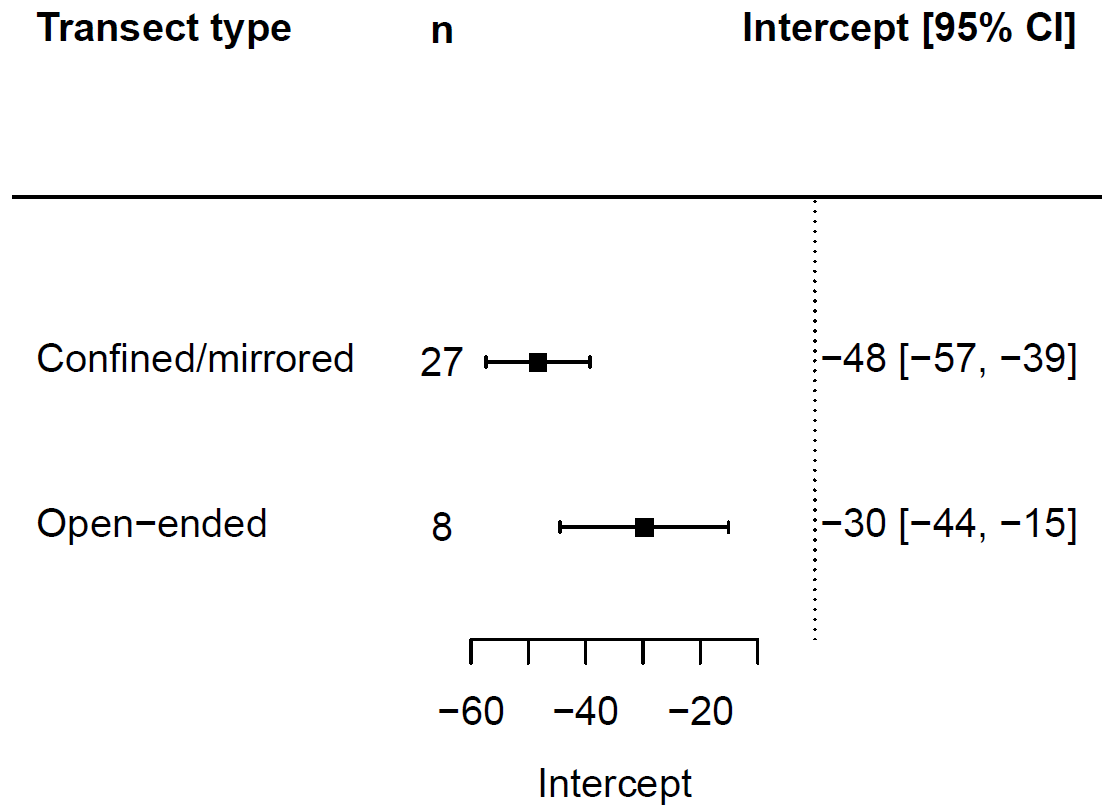


*Figure 14. Average effect sizes and confidence intervals for different types of transects, for the intercept of the groundwater level change vs distance relationship (n = 35).*

# Overall drainage effects

Study design was not a significant moderator (Figure 15).


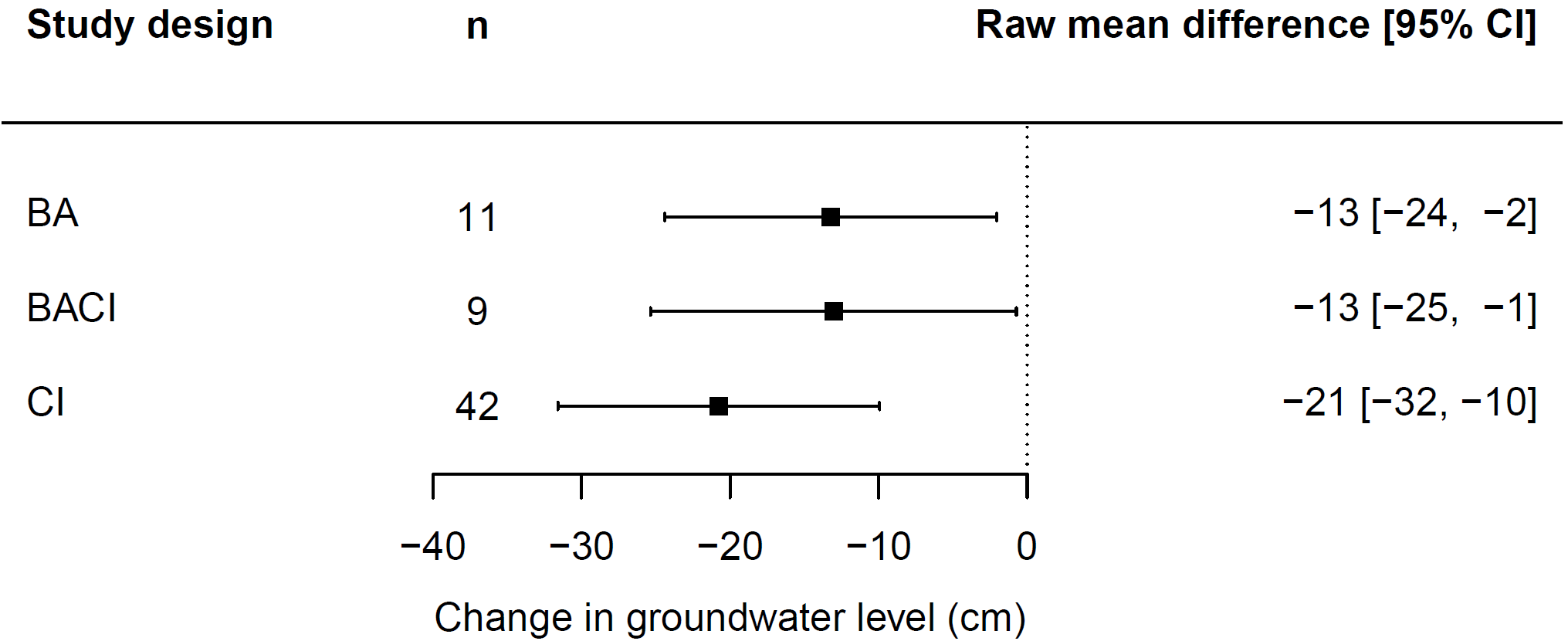


Figure 15. Average effect sizes and confidence intervals for different types of study designs, for overall effects in the wetland in the vicinity of the drainage (n =62).


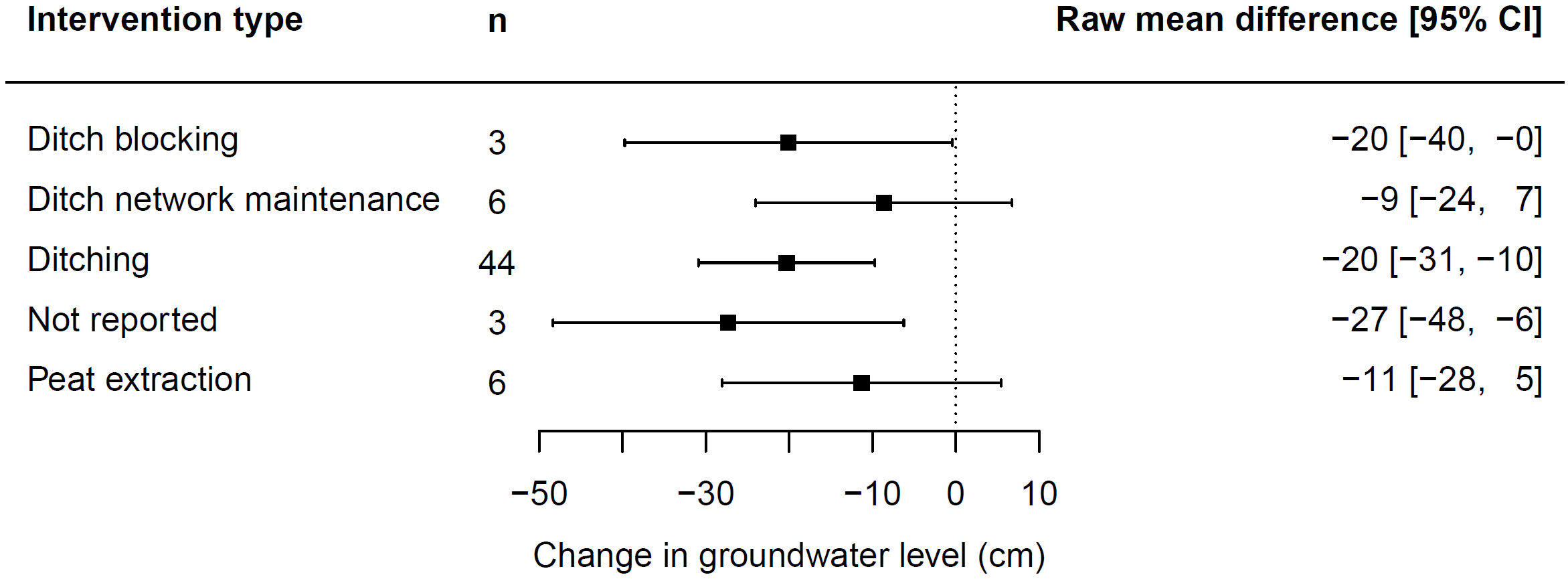


Figure 16. Average effect sizes and confidence intervals for different intervention types, for overall effects in the wetland in the vicinity of the drainage (n = 62).

The depth of the drainage intervention was not a significant moderator in the studies of restoration effects at different distances (n = 26). The relationship is not significant even when removing outliers at 150 cm.


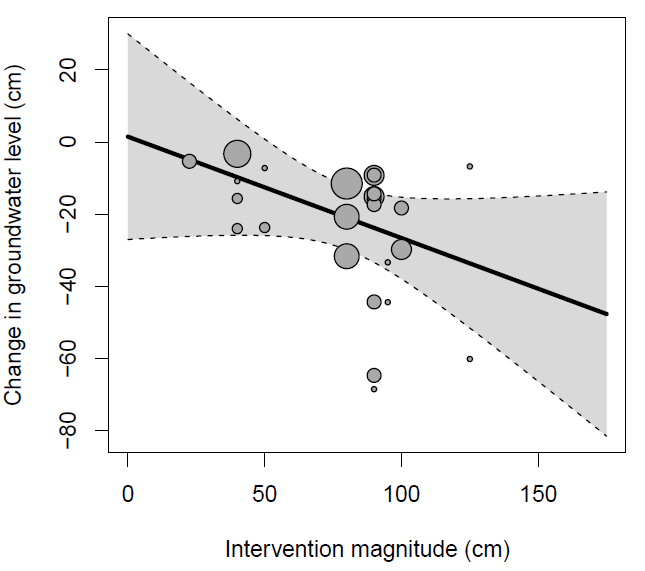


Figure 17. Meta-regression of the effect size against the drainage intervention magnitude for all studies that are not blanket peatlands (n = 26). Size of symbols represent the study weight in the meta-analysis (proportional to the number of wetlands in the original study).

Cumulative meta-analysis effect size calculated from adding one drainage study at a time in chronological order is shown in Figure 18. As for restoration, the analysis shows that the point estimate does not change much with the addition of the last third of studies, but there is some change in the width of the confidence interval which generally becomes slightly narrower with time. One visible exception occurs at the addition of Minkkinen 2020 which widens the confidence interval. This is because the Minkkinen study is so large (59 wetlands, carrying an overall weight of 20% in the entire meta-analysis). Even though the point estimate in Minkkinen is close to what the point estimate was before the addition (-18.4 cm vs the earlier estimate of -19.1 cm), the large weight and slight difference in point estimate is visible in the widening of the overall confidence interval (Figure 18).


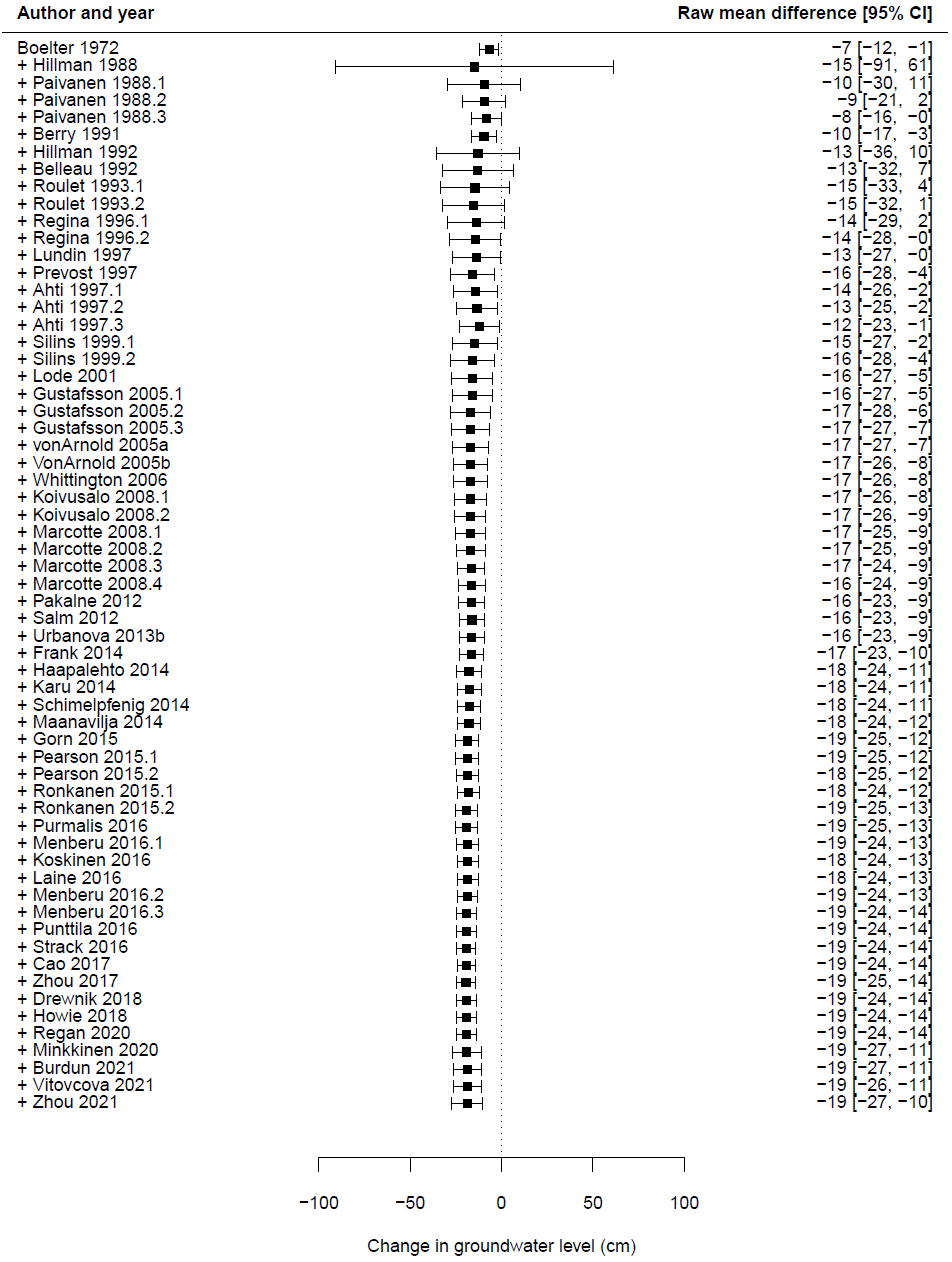


Figure 18. Average effect sizes and confidence intervals based on an incremental addition of each study in chronological order, for overall effects in the wetland in the vicinity of the restoration (n = 62).
