# Supplementary material for: Effects on groundwater storage of restoring, constructing or draining wetlands in temperate and boreal climates: a systematic review
Source: Environ Evid. 2022 Dec 8;11:38. doi: 10.1186/s13750-022-00289-5 (PMC11378806; doi:10.1186/s13750-022-00289-5)
Supplement: Supplementary file 9 — Additional file 9. Funnel plots. [file 13750_2022_289_MOESM9_ESM.docx]

Additional file 9. Funnel plots

In this file we present and discuss the results of analyses for publication bias through funnel plots. Although our main specification in the meta-analysis used sample size as study weights, the publication bias analysis presented here shows the study standard error. This is because sample size cannot be easily interpreted against the effect size in a traditional funnel plot.

# Restoration effects at different distances

There are relatively few studies (n = 8) of how the restoration effect varies with distance (Figure 1), and it is therefore difficult from the funnel plots to determine any clear indication of presence of publication bias. The trim and fill method (Duval and Tweedie, 2000a, 2000b) did not indicate any missing studies. Egger’s tests were not significant (p = 0.38 for slope and p = 0.64 for intercept, full statistics in Additional file 8).


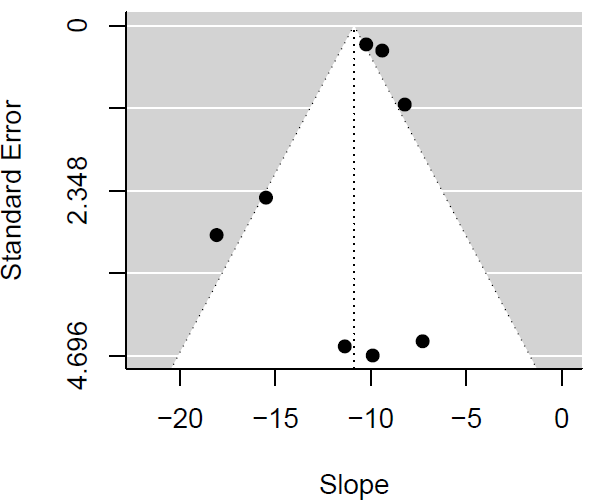

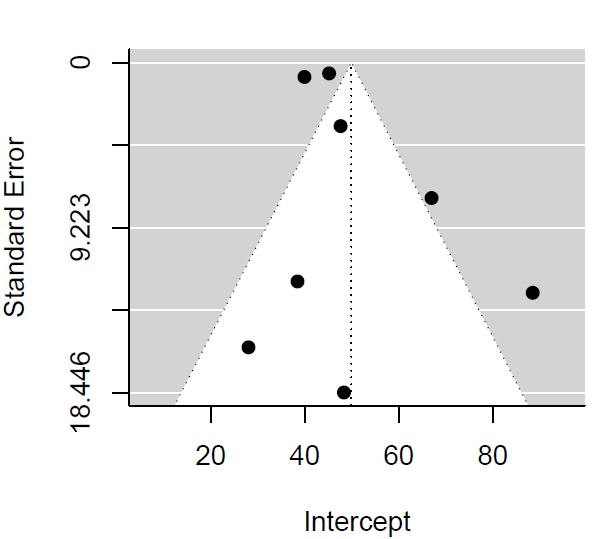


Figure 1. Funnel plots of the standard error of the effect (vertical axis) against the study effect size (horizontal axis) for studies that investigate restoration effects at different distances. Each dot corresponds to a study (n = 8).

# Overall restoration effects

The funnel plot of restoration studies (n = 47) has a few outliers (Figure 2). Results are therefore shown also without these studies (Figure 3). Although there may be a tendency for values with lower precision (larger standard errors) to show larger effects, the largest effect sizes have relatively small errors, and funnel plots do not show any clear indication of presence of publication bias. Still, Egger’s test was significant (p = 0.008), but the trim and fill method (Duval and Tweedie, 2000a, 2000b) did not indicate any missing studies and therefore no adjustment to the estimated effect size.


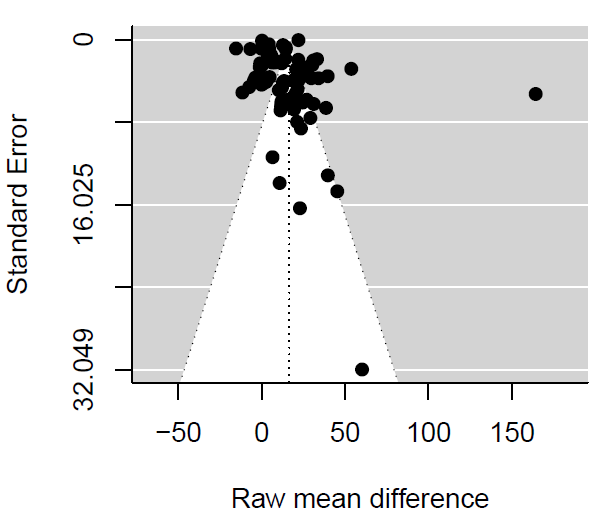


Figure 2. Funnel plots of the standard error of the effect (vertical axis) against the study effect size (horizontal axis) for studies that investigate overall restoration effects. Each dot corresponds to a study (n = 47).
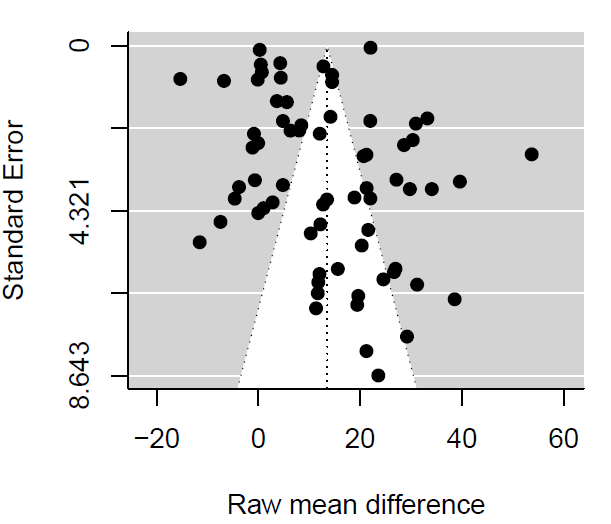


Figure 3. Funnel plots of the standard error of the effect (vertical axis) against the study effect size (horizontal axis) for studies that investigate overall restoration effects, excluding outliers. Each dot corresponds to a study (n = 40).

# Drainage effects at different distances

The funnel plot of studies that investigate drainage at different distances (n = 35) has a few outliers (Figure 4). Results are therefore shown also without these outlier studies (Figure 5). Funnel plots do not show any clear indication of presence of publication bias. The trim and fill method (Duval and Tweedie, 2000a, 2000b) added an estimated single missing study to the left side of the plot (Figure 6). However, the resulting adjusted meta-analysis point estimate and confidence interval only shifted marginally, by 0.1 cm or less. Egger’s tests were not significant (p = 0.14 for slope and p = 0.35 for intercept).


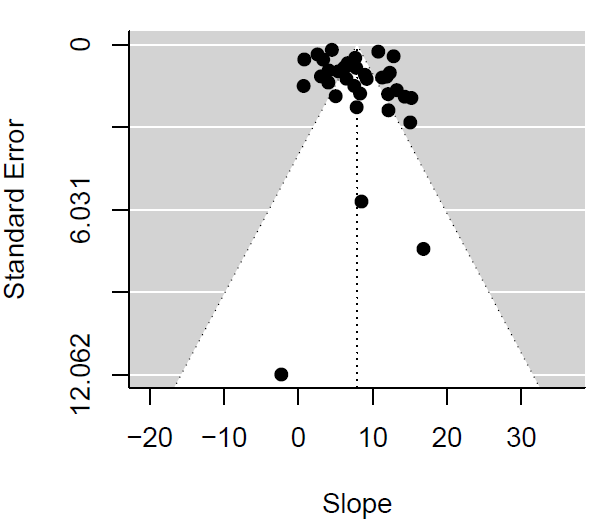

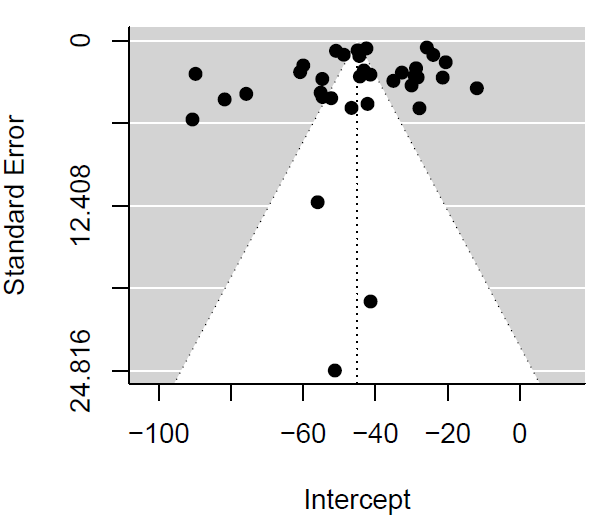


Figure 4. Funnel plots of the standard error of the effect (vertical axis) against the study effect size (horizontal axis) for studies that investigate drainage effects at different distances. Each dot corresponds to a study (n = 35).


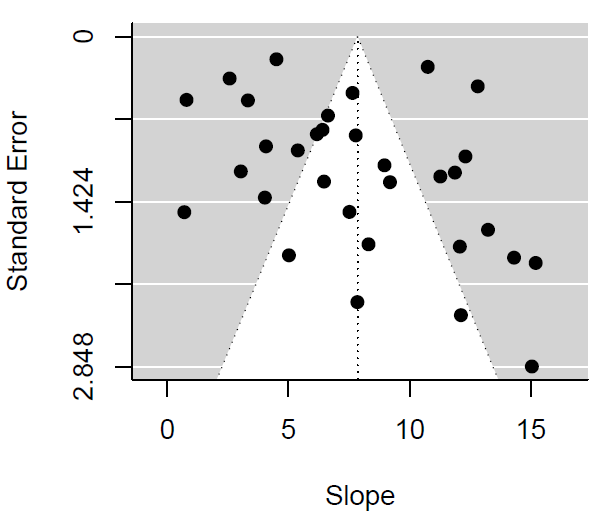

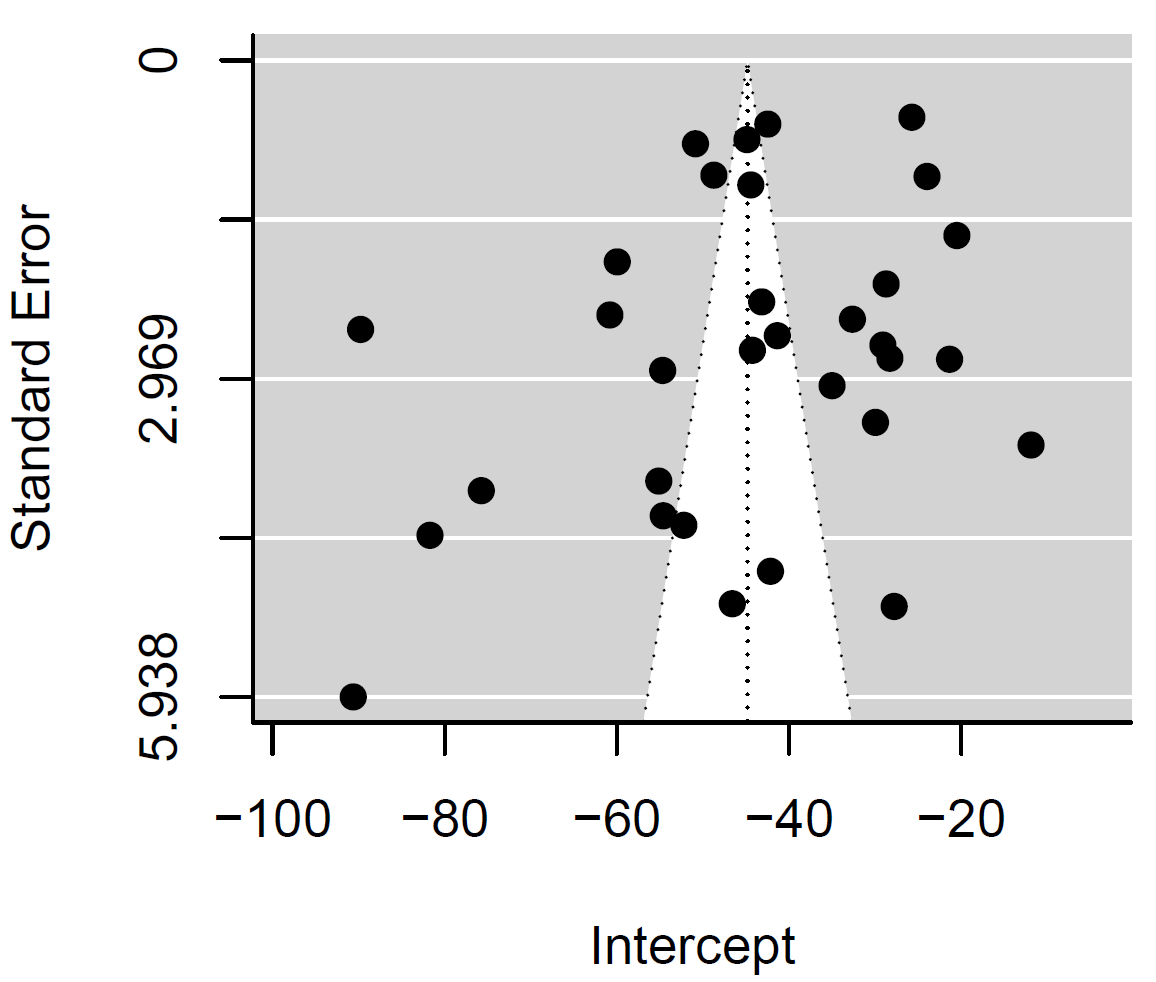


Figure 5. Funnel plots of the standard error of the effect (vertical axis) against the study effect size (horizontal axis) for studies that investigate drainage effects at different distances, excluding outliers. Each dot corresponds to a study (n = 32).


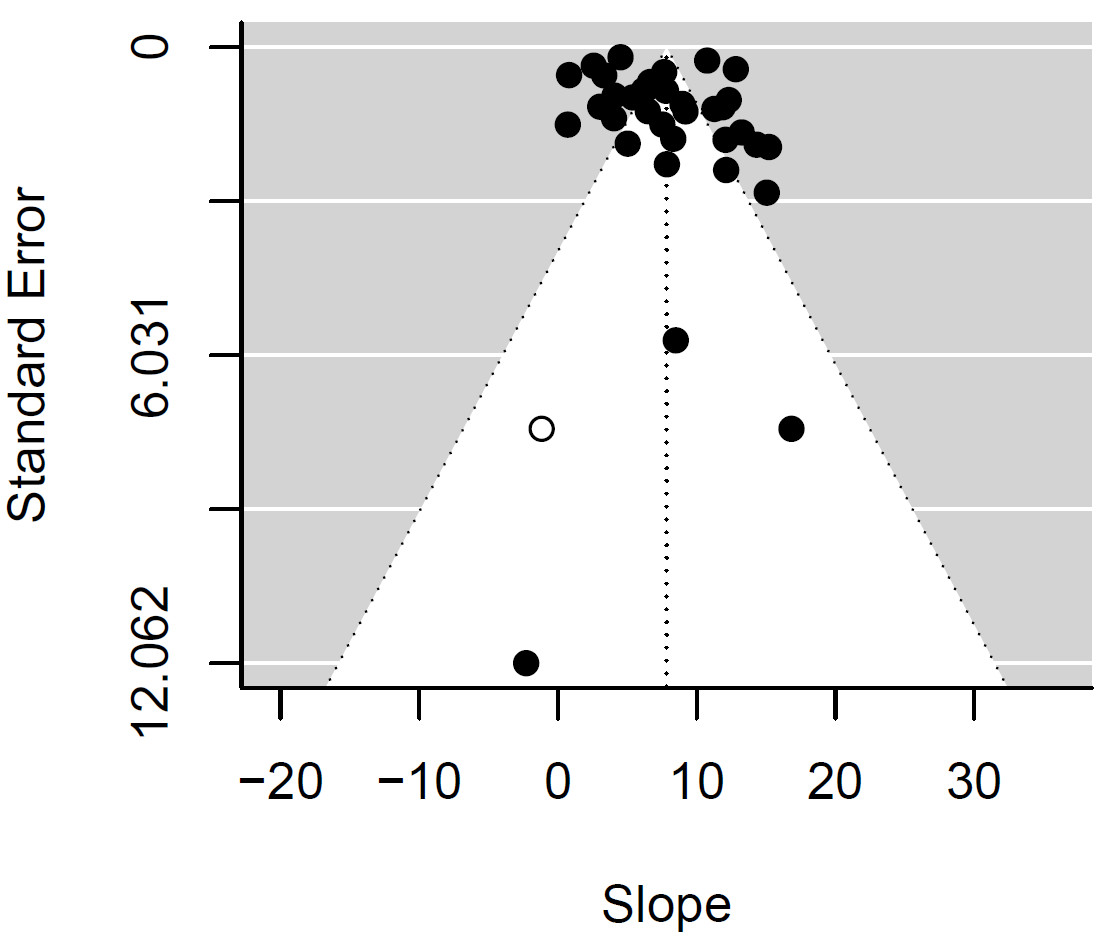


Figure 6. In-filled funnel plot, using the trim and fill method, of the standard error of the effect (vertical axis) against the study effect size (horizontal axis) for studies that investigate drainage effects at different distances. Each dot corresponds to a study. Solid dots are included studies from the literature (n = 35), the empty circle is an infilled study (n = 1).

# Overall drainage effects

The funnel plot of restoration studies (n = 62) has a few outliers (Figure 7). Results are therefore shown also without these studies (Figure 8). Funnel plots do not show any clear indication of presence of publication bias. Still, Egger’s test was significant (p = 0.008), but the trim and fill method (Duval and Tweedie, 2000a, 2000b) did not indicate any missing studies and therefore no adjustment to the estimated effect size.


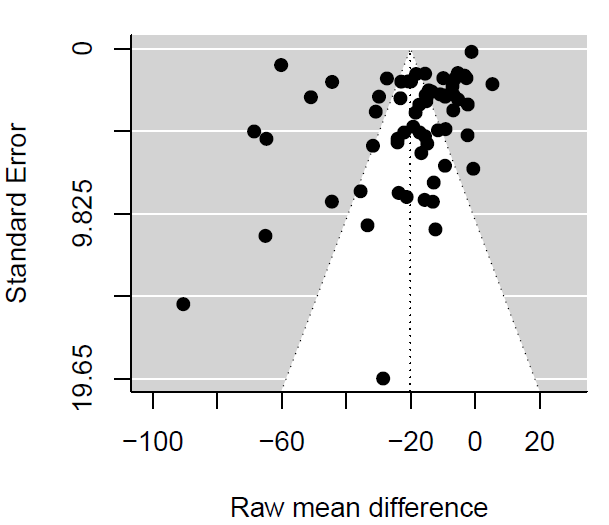


Figure 7. Funnel plots of the standard error of the effect (vertical axis) against the study effect size (horizontal axis) for studies that investigate overall drainage effects. Each dot corresponds to a study (n = 62).


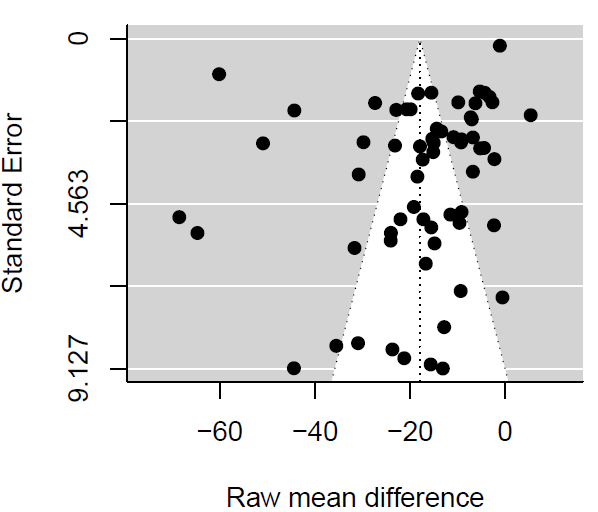


Figure 8. Funnel plots of the standard error of the effect (vertical axis) against the study effect size (horizontal axis) for studies that investigate overall drainage effects, excluding outliers. Each dot corresponds to a study (n = 57).
